# Androgen aggravates aortic aneurysms via suppression of PD-1 in mice

**DOI:** 10.1172/JCI169085

**Published:** 2024-06-20

**Authors:** Xufang Mu, Shu Liu, Zhuoran Wang, Kai Jiang, Tim McClintock, Arnold J. Stromberg, Alejandro V. Tezanos, Eugene S. Lee, John A. Curci, Ming C. Gong, Zhenheng Guo

**Affiliations:** 1 Departments of Pharmacology and Nutritional Sciences,; 2 Physiology, and; 3 Statistics, University of Kentucky, Lexington, Kentucky, USA.; 4 Department of Research, Sacramento Veterans Affairs Medical Center, Mather, California, USA.; 5 Department of Vascular Surgery, Vanderbilt University, Nashville, Tennessee, USA.; 6 Saha Cardiovascular Research Center, University of Kentucky, Lexington, Kentucky, USA.; 7 Department of Research, Lexington Veterans Affairs Medical Center, Lexington, Kentucky, USA.

**Keywords:** Inflammation, Vascular biology, Sex hormones, T cells, Vasculitis

## Abstract

Androgen has long been recognized for its pivotal role in the sexual dimorphism of cardiovascular diseases, including aortic aneurysms (AAs), a devastating vascular disease with a higher prevalence and fatality rate in men than in women. However, the mechanism by which androgen mediates AAs is largely unknown. Here, we found that male, not female, mice developed AAs when exposed to aldosterone and high salt (Aldo-salt). We revealed that androgen and androgen receptors (ARs) were crucial for this sexually dimorphic response to Aldo-salt. We identified programmed cell death protein 1 (PD-1), an immune checkpoint, as a key link between androgen and AAs. Furthermore, we demonstrated that administration of anti–PD-1 Ab and adoptive PD-1–deficient T cell transfer reinstated Aldo-salt–induced AAs in orchiectomized mice and that genetic deletion of PD-1 exacerbated AAs induced by a high-fat diet and angiotensin II (Ang II) in nonorchiectomized mice. Mechanistically, we discovered that the AR bound to the PD-1 promoter to suppress the expression of PD-1 in the spleen. Thus, our study unveils a mechanism by which androgen aggravates AAs by suppressing PD-1 expression in T cells. Moreover, our study suggests that some patients with cancer might benefit from screenings for AAs during immune checkpoint therapy.

## Abstract 2

Androgen aggravates aortic aneurysms via suppression of programmed cell death protein 1 (PD-1) expression in splenic T cells.

## Graphical abstract

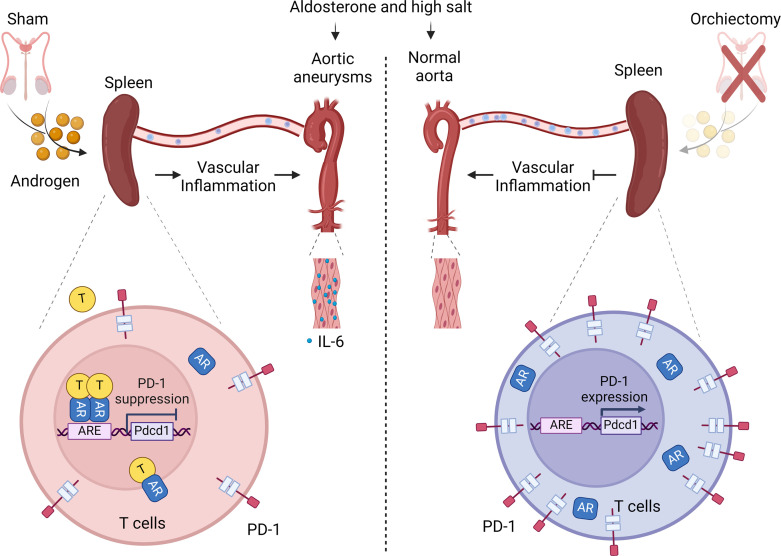

## Introduction

Aortic aneurysms (AAs) are defined as a permanent localized dilation of the aorta and can be classified as thoracic aortic aneurysms (TAAs) and abdominal aortic aneurysms (AAAs) (1). AAs are usually asymptomatic until they rupture and are often lethal, resulting in over 85% mortality (2). Currently, no medication except for surgery is approved to treat this devastating vascular disease.

Epidemiologic studies reveal aging, male sex, smoking, atherosclerosis, and hypertension as the risk factors for AAs (3). In particular, male sex is considered the most potent nonmodified risk factor for the sexual dimorphism of AAs, with a 4:1 male/female ratio (3). While the etiology of the sex difference in human AAs remains to be elucidated, accumulated evidence from animal studies demonstrates that both sex chromosomes and hormones contribute to the development of AAs (4). In particular, it is well documented that gonadal androgen but not estrogen deprivation protects against angiotensin II– (Ang II–) or elastase-induced AAs (5–7), indicating that androgen likely plays a predominant role in the sexual dimorphism of AAs. However, the mechanism by which androgen aggravates Ang II– or elastase-induced AAs remains largely unknown.

Accumulated clinical evidence demonstrates that elevated plasma concentrations of aldosterone (Aldo), an essential component of the renin-angiotensin-aldosterone system, and excessive dietary sodium intake are associated with an increased risk for hypertension, stroke, coronary heart disease, heart failure, and renal disease (8). Consistent with these human studies, we developed a mouse model of AAs in which we administered Aldo and high salt (Aldo-salt) to 10-month-old male C57BL/6J mice (9, 10). Importantly, we demonstrated that Aldo-salt–induced AAs depended on age and mineralocorticoid receptor (MR; also known as the Aldo receptor) but not the Ang II receptor (9, 10). However, whether Aldo-salt–induced AAs have a sexual dimorphism has not, to our knowledge, been investigated.

In this study, we report that Aldo-salt–induced AAs mimicked human AAs, exhibiting a strong sexual dimorphism. To delve into the role of androgen in this sexual dimorphism, we conducted a series of animal experiments, including gonadal androgen deprivation via orchiectomy, restoration of androgen in orchiectomized mice through dihydrotestosterone (DHT) pellet implantation, and downregulation of androgen receptors (ARs) by ASC-J9 or inhibition of the AR by flutamide. Our results consistently underscored the critical involvement of androgen and the AR in Aldo-salt–induced AAs. To investigate the mechanism by which androgen mediates Aldo-salt–induced AAs, we found that Aldo-salt–induced IL-6 expression was selectively abolished in the aorta by orchiectomy. Subsequent inhibition of IL-6 signaling by LMT-28 illustrated that IL-6 is implicated in Aldo-salt–induced AAs. Moreover, through RNA-Seq and flow cytometric analysis of aortas, we identified T cell receptor (TCR) and programmed cell death protein 1 (PD-1), an immune checkpoint (11), as a pivotal link between androgen and Aldo-salt–induced AAs. Splenectomy augmented PD-1^+^ T and B cells in the aorta and mitigated Aldo-salt–induced AAs. Mechanistically, we discovered that the AR bound to the PD-1 promoter and suppressed its mRNA and protein expression in the spleen in mice administered Aldo-salt. To define the role of PD-1 in the pathogenesis of AAs, we demonstrated that immune checkpoint blockade with anti–PD-1 Ab and adoptive PD-1–deficient T cell transfer restored Aldo-salt–induced aortopathies in orchiectomized mice. Finally, we showed that genetic deletion of PD-1 exacerbated high-fat diet– (HFD-) and Ang II–induced aortopathy in nonorchiectomized mice. Collectively, our results provide mechanistic insight into the role of androgen in the pathogenesis of AAs and suggest a potential risk of AA development in patients with cancer undergoing immune checkpoint therapy.

## Results

### Sexual dimorphism in Aldo-salt–induced AAs.

**Figure 1:**
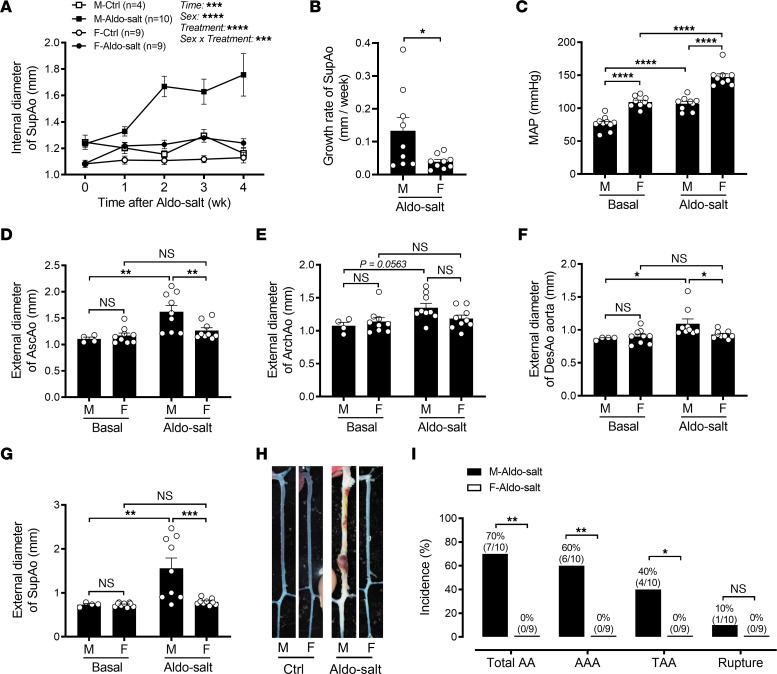
Sexual dimorphism in Aldo-salt–induced AAs. (**A** and **B**) Maximal internal diameters (**A**) and growth rates (**B**) of the SupAo were measured in vivo by ultrasound weekly in 10-month-old male (M) and female (F) C57BL/6J mice administered Aldo-salt. Time 0 represents measurements 1 week before Aldo-salt administration (*n* = 4–10/group). (**C**) MAP was measured by tail cuff in mice 1 week before (basal) and 3 weeks after Aldo-salt administration (*n* = 9–10/group). (**D**−**G**) Maximal external diameters of the AscAo, ArchAo, DesAo, and SupAo were measured ex vivo by microscopy 4 weeks after Aldo-salt administration (*n* = 4–9/group). (**H**) Representative photographs of aortas with and without AAs. (**I**) Incidence of total AAs, AAAs, TAAs, and aortic ruptures (mice with AAs/total male or female mice administered Aldo-salt). M-Aldo-salt, male mice administered Aldo-salt; F-Aldo-salt, female mice administered Aldo-salt. Data are expressed as the mean ± SEM and were analyzed by 3-way ANOVA (**A**), 2-tailed, unpaired *t* test (**B**), 2-way ANOVA with multiple-comparison test (**C**−**G**), and 2-sided χ^2^ test (**I**). *P < 0.05, **P < 0.01, ***P < 0.001, and ****P < 0.0001. Ctrl, control.

To investigate sexual dimorphism in Aldo-salt–induced AAs, 10-month-old male and female C57BL/6J mice were subjected to Aldo-salt administration for 4 weeks to induce AAs (9, 10). Suprarenal aortic dilations induced by Aldo-salt were monitored weekly by ultrasound (9, 10). Although Aldo-salt induced suprarenal aortic dilation in both male and female mice in a time-dependent manner, the suprarenal aortic dilation induced by Aldo-salt was much larger in male mice than in female mice (Figure 1A). Utilizing the ultrasound data, we calculated the growth rate of the suprarenal aortic diameters, an important clinical index for appraisal of AA progression and rupture in patients (4). Consistently, we found that the suprarenal aortic growth rate was significantly more accelerated in male mice than in female mice (Figure 1B).

To define the role of hypertension in the sexual dimorphism of Aldo-salt–induced AAs, we assessed mean arterial pressure (MAP) in male and female mice by tail cuff 1 week before and 3 weeks after Aldo-salt administration. Both male and female mice displayed a hypertensive response to Aldo-salt. Surprisingly, the female mice had higher MAP levels than did the male mice before and after Aldo-salt administration (Figure 1C), suggesting that the greater increase in suprarenal aortic dilation induced by Aldo-salt was not attributable to hypertension.

Four weeks after Aldo-salt administration, we harvested the aortas from the male and female mice for morphometric analysis. Maximal external diameters of the ascending aorta (AscAo), aortic arch (ArchAo), descending aorta (DesAo), and suprarenal aorta (SupAo) were measured (Supplemental Figure 1; supplemental material available online with this article; https://doi.org/10.1172/JCI169085DS1). Although no differences were observed at baseline, we noted a significant increase in the external diameters of the AscAo, DesAo, and SupAo in response to Aldo-salt administration in male mice compared with female mice (Figure 1, D−G). Isolated aortas were also subjected to pathological analysis to assess the incidence of the total AAs (total AAs = AAAs + TAAs + aortic ruptures), AAAs, TAAs, and aortic ruptures (Figure 1H) (9, 10, 12). Remarkably, none of the female mice developed AAs, whereas 70% of male mice had AAs, 60% of which were AAAs, 40% were TAAs, and 10% were aortic ruptures (Figure 1I).

### Gonadal androgen deprivation protects mice from Aldo-salt–induced AAs.

**Figure 2:**
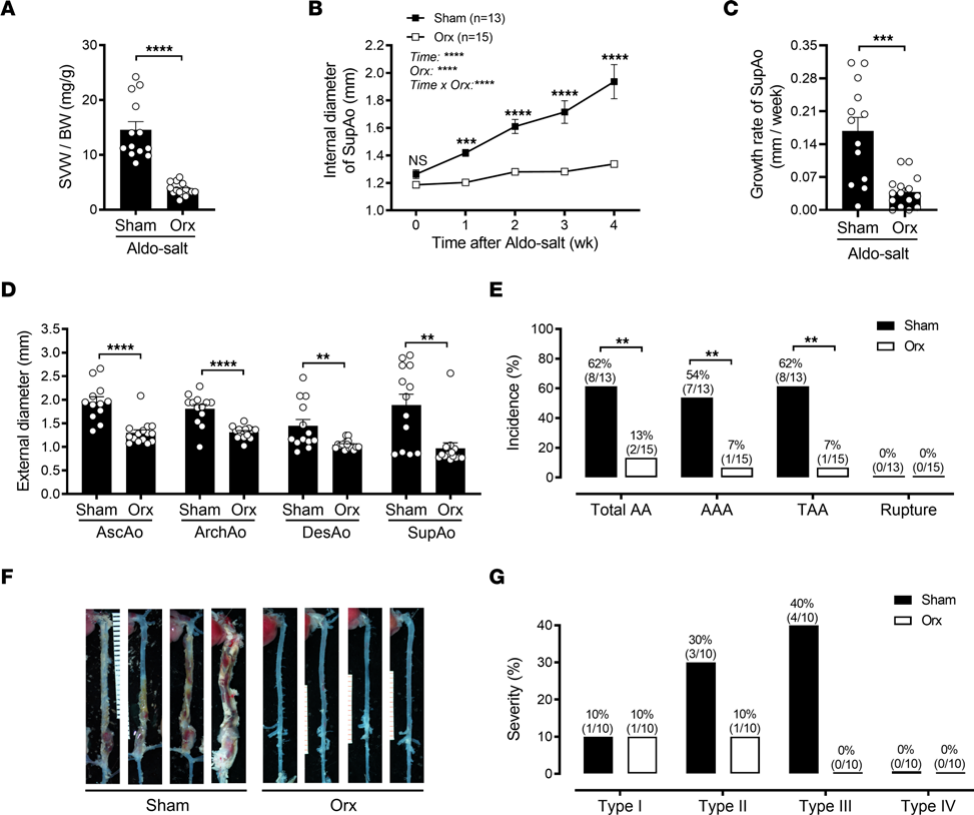
Orchiectomy protects mice from Aldo-salt–induced aortic dilation, progression, and aneurysm formation. (**A**) The SVW/BW ratio was determined in orchiectomized (Orx) and sham-operated 10-month-old male C57BL/6J mice 4 weeks after Aldo-salt administration (*n* = 13–15/group). (**B** and **C**) Maximal internal diameters and growth rate of the SupAo (*n* = 13–15/group). ***P < 0.001 and ****P < 0.0001, vs. Orx at 1, 2, 3, and 4 week, respectively. (**D**) Maximal external diameters of the AscAo, ArchAo, DesAo, and SupAo (*n* = 12–15/group). (**E**) Incidence of total AAs, AAAs, TAAs, and aortic ruptures. (**F**) Representative images of aortas with and without AAs. (**G**) Severity of AAs (see also Supplemental Figure 3). Data are expressed as the mean ± SEM and were analyzed by 2-tailed, unpaired *t* test (**A**, **C**, and **D**), 2-way ANOVA with multiple-comparison test (**B**), and 2-sided χ^2^ test (**E**). **P < 0.01, ***P < 0.001, and ****P < 0.0001.

To explore the role of androgen in the sexual dimorphism of Aldo-salt–induced AAs, 10-month-old male C57BL/6J mice underwent either orchiectomy or sham operation and 2 weeks later were administered Aldo-salt for 4 weeks. The ratio of seminal vesicle weight (SVW) to BW was evaluated 4 weeks after Aldo-salt administration to confirm the success of orchiectomy. We observed significantly reduced SVW/BW ratios in orchiectomized mice compared with their sham-operated counterparts (Figure 2A and Supplemental Figure 2, A and B). Additionally, serum testosterone levels were significantly lower in orchiectomized mice than in sham-operated mice (Supplemental Figure 2C). Importantly, compared with the sham operation, orchiectomy markedly suppressed Aldo-salt–induced suprarenal aortic dilation and progression (Figure 2, B and C), growth of the external diameter of aortas, including in the AscAo, ArchAo, DesAo, and SupAo (Figure 2D), and the incidence of AAAs and TAAs (Figure 2E).

The severity of Aldo-salt–induced AAs resembles human AAs (13) and varies significantly (9, 10). Similar to Ang II–induced AAs (12), the severity of Aldo-salt–induced AAs could be categorized into types I, II, III, and IV (Supplemental Figure 3). Compared with sham operation, we found that orchiectomy reduced the percentage of types II and III but not type I AAs (Figure 2, F and G), indicating that androgen may mainly affect the progression of Aldo-salt–induced AAs.

To investigate whether androgen augments Aldo-salt–induced AAs through salt retention, we assessed 24-hour sodium retention in orchiectomized and sham-operated mice by subtracting their 24-hour sodium excretion (via urine) from their sodium intake (via food and water intake) (14) 1 week before and 3 weeks after Aldo-salt administration. Interestingly, orchiectomy increased both 24-hour sodium intake and 24-hour urinary sodium excretion compared with the sham operation (Supplemental Figure 4, A−E). As a net balance, orchiectomy did not significantly alter Aldo-salt–induced sodium retention (Supplemental Figure 4F). Consistent with these findings, we observed no significant difference in serum sodium levels between the orchiectomized and sham-operated mice, and we found no correlation between the serum sodium level and the internal diameter of the SupAo 4 weeks after Aldo-salt administration (Supplemental Figure 4, G and H). Additionally, serum sodium levels did not differ between the orchiectomized and sham-operated mice, regardless of whether the mice developed AAs (Supplemental Figure 4I).

To determine whether androgen augments Aldo-salt–induced AAs through hypertension, we evaluated the effect of orchiectomy on MAP by tail-cuff measurement 1 week before and 3 weeks after Aldo-salt administration. In line with its minimal effect on sodium retention, we found that orchiectomy did not affect MAP before or after Aldo-salt administration (Supplemental Figure 4J). Moreover, no correlation was observed between MAP and the internal diameter of the SupAo 3 weeks after Aldo-salt administration (Supplemental Figure 4K), and no significant disparities in MAP were noted between orchiectomized and sham-operated mice, regardless of whether AAs developed (Supplemental Figure 4L). Similar findings were also noted with regard to systolic and diastolic blood pressure (our unpublished observations).

### Restoration of androgen in orchiectomized mice reinstates Aldo-salt–induced AAs.

**Figure 3:**
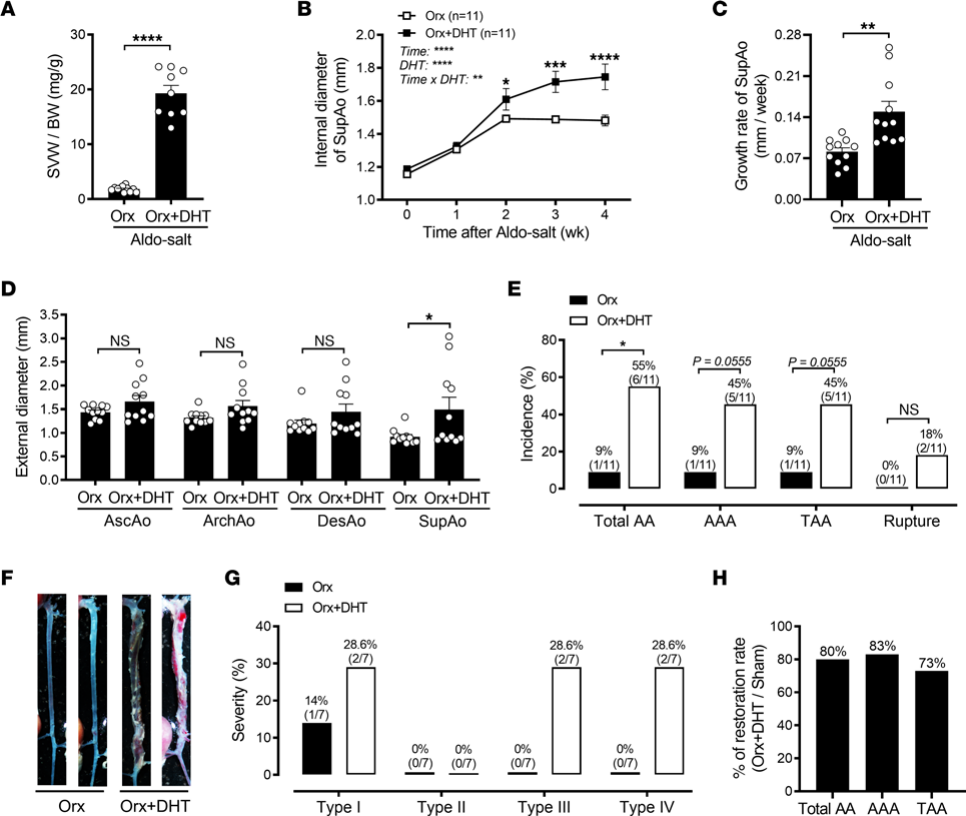
Exogenous DHT administration to orchiectomized male mice restores Aldo-salt–induced AAs. (**A**) The SVW/BW ratio was determined in 10-month-old orchiectomized C57BL/6J mice 4 weeks after Aldo-salt administration with and without DHT pellet implantation (*n* = 9–11/group). (**B** and **C**) Maximal intraluminal diameters and growth rate of the SupAo (*n* = 11/group). *P <0.05, ***P < 0.001, and ****P < 0.0001, vs. Orx at 2, 3, and 4 weeks, respectively. (**D**) Maximal external diameters of the AscAo, ArchAo, DesAo, and SupAo (*n* = 11/group). (**E**) Incidence of total AAs, AAAs, TAAs, and aortic ruptures. (**F**) Representative photographs of aortas with and without AAs. (**G**) Severity of AAs. (**H**) Restoration rates of AAs = mice with orchiectomy and DHT and AAs/mice with sham operation, with and without AAs. Data are expressed as the mean ± SEM and were analyzed by 2-tailed, unpaired *t* test (**A**, **C**, and **D**), 2-way ANOVA with multiple-comparison test (**B**), and 2-sided χ^2^ test (**E**). *P <0.05, **P < 0.01, ***P < 0.001, and ****P < 0.0001.

To further elucidate the role of androgen in Aldo-salt–induced AAs, 10-month-old male C57BL/6J mice underwent orchiectomy, and 2 weeks later, they were administered Aldo-salt with or without DHT pellet implantation (10 mg, 60-day release (6)) for 4 weeks. DHT was chosen over testosterone because of its greater potency and inability to be converted to estrogens by aromatase (15), ensuring a more straightforward interpretation of the results. DHT effectively restored the SVW/BW ratio in orchiectomized mice to levels comparable to those in sham-operated mice, 4 weeks after Aldo-salt administration (Figure 3A, Supplemental Figure 2, A and B, and Supplemental Figure 5), indicating the functionality of the implanted DHT pellets in mice. Importantly, compared with orchiectomized mice without DHT, those given DHT exhibited a trend or significant increase in the internal diameter and growth rate of the SupAo in response to Aldo-salt (Figure 3, B and C), the external diameters of the AscAo, ArchAo, DesAo, and SupAo (Figure 3D), and the incidence of AAs, including AAAs, TAAs, and aortic ruptures (Figure 3E).

The aortopathy in orchiectomized mice administered Aldo-salt with DHT appeared more pronounced than in sham-operated mice (compare Figure 3, F and G with Figure 2, F and G). To quantitatively assess the extent to which DHT restores Aldo-salt–induced AAs, we calculated the percentage of AA restoration rates by normalizing the incidence of AAs in orchiectomized mice with DHT (Figure 3E) to that in sham-operated mice (Figure 2E). As a result, 80%, 83%, and 73% of the AA restoration rates occurred in orchiectomized mice administered Aldo-salt with DHT for total AAs, AAAs, and TAAs, respectively (Figure 3H).

Surprisingly, DHT significantly suppressed 24-hour sodium intake and 24-hour urinary sodium excretion 3 weeks after Aldo-salt administration (Supplemental Figure 6, A−E). However, DHT did not significantly affect Aldo-salt–induced sodium retention, serum sodium levels, or hypertension (Supplemental Figure 6, F, G, and J). We found no significant correlation between the internal diameter of the SupAo and serum sodium levels or MAP in orchiectomized mice administered Aldo-salt with or without DHT (Supplemental Figure 6, H and K). Furthermore, there was no significant difference in serum sodium or MAP levels in orchiectomized mice administered Aldo-salt with or without DHT, regardless of whether they developed AAs (Supplemental Figure 6, I and L). Thus, DHT is unlikely to restore Aldo-salt–induced AAs through sodium retention and hypertension.

### Downregulation of the AR ameliorates Aldo-salt–induced AAs.

**Figure 4:**
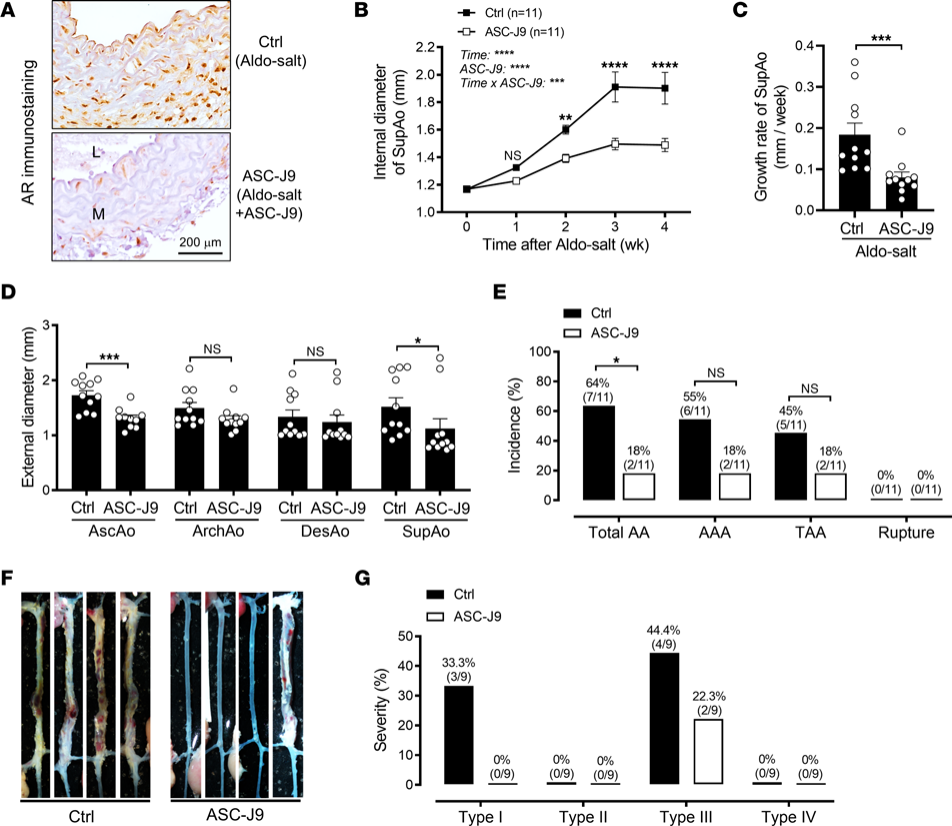
Downregulation of the AR by ASC-J9 in mice inhibits Aldo-salt–induced AA. (**A**) Representative immunostaining for the AR in SupAos from 10-month-old male C57BL/6J mice 4 weeks after Aldo-salt with and without ASC-J9 administration (*n* = 3/group). L, lumen; M, media. Scale bar: 200 μm. (**B** and **C**) Maximal internal diameters and growth rate of the SupAo (*n* = 11/group). **P < 0.01 and ***P < 0.001, vs. ASC-J9 at 2, 3, and 4 weeks, respectively. (**D**) Maximal external diameters of the AscAo, ArchAo, DesAo, and SupAo (*n* = 11/group). (**E**) Incidence of total AAs, AAAs, TAAs, and aortic ruptures. (**F**) Representative photographs of the aortas with and without AAs. (**G**) Severity of AAs. Data are expressed as the mean ± SEM and were analyzed by 2-way ANOVA with multiple-comparison test (**B**), 2-tailed, unpaired *t* test (**C** and **D**), and 2-sided χ^2^ test (**E**). *P < 0.05, **P < 0.01, ***P < 0.001, and ****P < 0.0001.

To explore androgen targeting as a potential therapy for AA, 10-month-old male C57BL/6J mice were administered Aldo-salt with or without ASC-J9 (50 mg/kg, i.p. injection, once a day) for 4 weeks (16). ASC-J9, a recently developed AR degradation enhancer, has been shown to selectively degrade the AR without affecting other nuclear receptors (17). The efficacy of ASC-J9 in promoting AR protein degradation was confirmed via IHC in the SupAo of mice 4 weeks after Aldo-salt administration, with or without ASC-J9 (Figure 4A). Importantly, similar to the effect of orchiectomy (Figure 2), ASC-J9 effectively mitigated Aldo-salt–induced suprarenal aortic dilation and progression (Figure 4, B and C), the external diameters of the AscAo and SupAo (Figure 4D), and the incidence and severity of AAs (Figures 4, E−G).

Intriguingly, ASC-J9 suppressed Aldo-salt–induced 24-hour sodium intake and urinary sodium excretion (Supplemental Figure 7, A−E). However, similar to the effect of orchiectomy and DHT (Supplemental Figures 4 and 6), ASC-J9 also did not affect Aldo-salt–induced sodium retention, hypernatremia, or hypertension (Supplemental Figure 7, F, G, and J). We found no significant correlation between the internal diameters of the SupAo and serum sodium levels or MAP in mice administered Aldo-salt with or without ASC-J9 (Supplemental Figure 7, H and K). Furthermore, there was no significant difference in serum sodium levels or MAP between mice with and without ASC-J9, regardless of whether they developed AAs (Supplemental Figure 7, I and L). Thus, ASC-J9 is unlikely to protect mice from Aldo-salt–induced AAs through sodium retention and hypertension.

ASC-J9 was reported to exert its effects through AR-dependent and -independent mechanisms (18). To verify whether ASC-J9 protects mice from Aldo-salt–induced AAs via the AR, we treated 9- to 10-month-old male C57BL/6J mice with Aldo-salt and flutamide (50 mg/kg/day, i.p. injection, once a day) or vehicle for 4 weeks (19). Flutamide, a selective AR antagonist, has been used clinically to treat patients with prostate cancer (19). In line with the effects of ASC-J9 (Figure 4), flutamide reduced the SVW (Supplemental Figure 8, A−C) and, more important, protected mice from Aldo-salt–induced suprarenal aortic dilation and progression and the incidence of AAA (Supplemental Figure 8, D−G). Interestingly, flutamide did not affect basal MAP but moderately boosted Aldo-salt–induced hypertension (Supplemental Figure 8H).

### IL-6 is implicated in Aldo-salt–induced and androgen-mediated AAs.

To investigate the molecular mechanism by which androgen mediates Aldo-salt–induced AAs, we performed real-time PCR to analyze mRNA expression levels in the aortas of 10-month-old orchiectomized and sham-operated C57BL/6J mice that were treated with or without Aldo-salt for 10 days. We opted to isolate the aortas 10 days rather than 4 weeks after Aldo-salt administration because we sought to identify androgen-targeting genes that result in, rather than result from, Aldo-salt–induced AAs.

On the basis of the literature (9, 10, 16, 20–22), we focused on a list of genes implicated in AAs, including *Ar*, *Nr3c2*, *Sgk1*, *Scnn1a*, *Scnn1b*, *Scnn1g*, *Bmal1*, *Tgfb2*, *Mmp2*, *Il1b*, *Il6*, *Il6ra*, *Il6st*, *Ccl2*, *Ccl4*, and *Tnf*. Of the 16 genes examined, 12 genes (*Ar*, *Nr3C2*, *Sgk1*, *Scnn1a*, *Scnn1b*, *Scnn1g*, *Bmal1*, *Il1b*, *Il6*, *Il6ra*, *Ccl2*, and *Ccl4*) responded to Aldo-salt: 9 of these genes (*Ar*, *Nr3C2*, *Sgk1*, *Scnn1a*, *Scnn1b*, *Scnn1g*, *Il1b*, *Il6ra*, and *Ccl4*) were downregulated, whereas 3 of them (*Bmal1*, *Il6*, and *Ccl2*) were upregulated (Supplemental Figure 9). Interestingly, 5 genes (*Nr3C2*, *Scnn1a*, *Scnn1b*, *Il6*, and *Ccl2*) also responded to orchiectomy after Aldo-salt administration: 3 of them (*Nr3C2*, *Scnn1a*, *Scnn1b*) were upregulated, whereas 2 of them (*Il6*, and *Ccl2*) were downregulated (Supplemental Figure 9, B, D, E, K, and N). Of particular note, *Il6* was the most dramatically upregulated by Aldo-salt (i.e., up to 58-fold), and this was completely abolished by orchiectomy (Supplemental Figure 9K).

IL-6 is implicated in human AAs (22). Therefore, we focused on IL-6 and further investigated whether its protein expression is regulated by Aldo-salt and/or androgen in the SupAo of 10-month-old orchiectomized and sham-operated C57BL/6J mice with and without 10-day Aldo-salt administration. In sham-operated mice, basal IL-6 protein expression was barely detectable in the SupAo, whereas IL-6 protein was markedly upregulated by Aldo-salt in the SupAo. Intriguingly, orchiectomy increased basal IL-6 protein expression but decreased Aldo-salt–induced IL-6 protein upregulation in the SupAo (Supplemental Figure 10, A and B). In contrast to IL-6 protein, MR protein levels were not affected by Aldo-salt and/or orchiectomy in the SupAo (Supplemental Figure 10, C and D).

While the elevated basal IL-6 protein levels in the SupAo induced by orchiectomy may be attributed to the loss of androgen-induced immunosuppression (15), the abrogation of Aldo-salt–induced IL-6 protein upregulation elicited by orchiectomy might be due to a blockade of Aldo-salt–induced inflammatory cell infiltration in the aorta (9, 10), specifically macrophages, which are known for their pivotal role in IL-6 production (8). To explore this possibility, we investigated whether orchiectomy affects Aldo-salt–induced macrophage infiltration by IHC in the SupAo of 10-month-old male C57BL/6J mice with and without 10-day Aldo-salt administration. Interestingly, we found that orchiectomy abolished Aldo-salt–induced immunostaining of F4/80, a macrophage marker, in the SupAo (Supplemental Figure 11, A and B).

**Figure 5:**
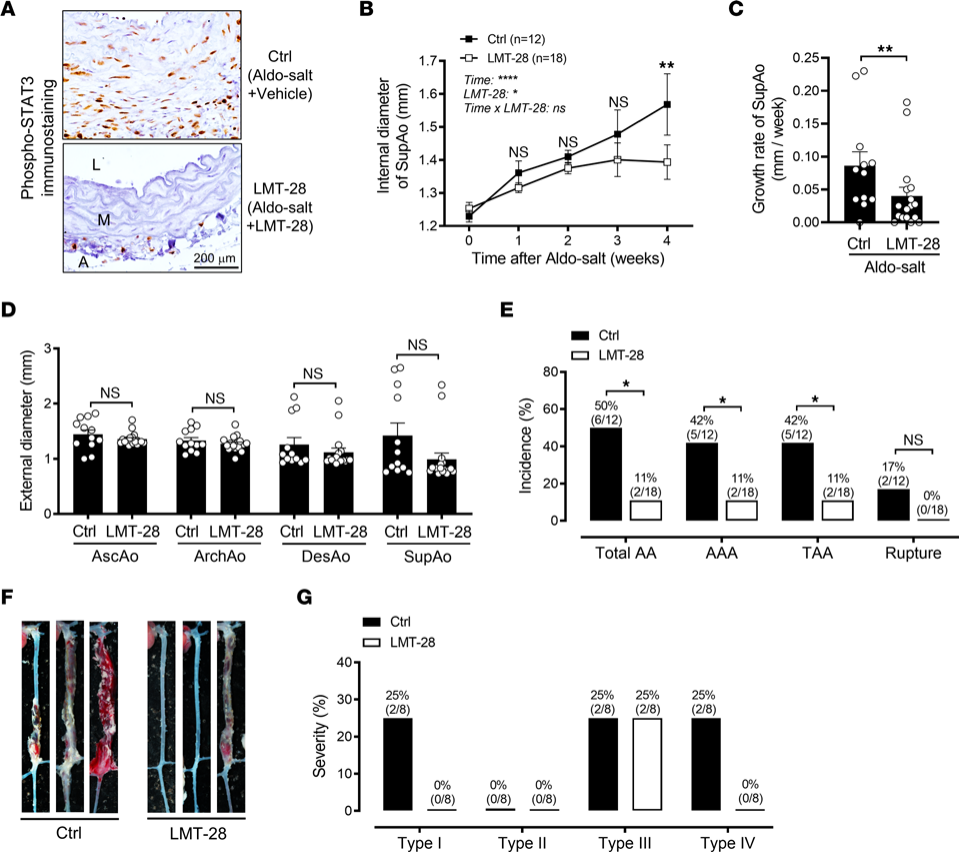
Inhibition of IL-6 signaling by LMT-28 ameliorates Aldo-salt–induced AAs. (**A**) Representative immunostaining for STAT3 phosphorylation at Tyr705 in SupAos from 10-month-old male C57BL/6J mice 4 weeks after Aldo-salt administration with LTM-28 or vehicle control (Ctrl) (*n* = 3/group). Scale bar: 200 μm. A, adventitia. (**B** and **C**) Maximal internal diameters and growth rate of the SupAo (*n* = 12–18/group). **P < 0.01, vs. LMT-28 at 4 weeks. (**D**) Maximal external diameters of the AscAo, ArchAo, DesAo, and SupAo (*n* = 12–17/group). (**E**) Incidence of total AAs, AAAs, TAAs, and aortic ruptures. (**F**) Representative photographs of aortas with and without AAs. (**G**) Severity of AAs. Data are expressed as the mean ± SEM and were analyzed by 2-way ANOVA with multiple-comparison test (**B**), 2-tailed, unpaired *t* test (**C** and **D**), and 2-sided χ^2^ test (**E**). *P < 0.05, **P < 0.01, and ****P < 0.0001.

To investigate the potential role of IL-6 in Aldo-salt–induced AAs, 10-month-old male C57BL/6J mice were given Aldo-salt with LMT-28 (0.25 mg/kg, oral gavage, once a day) or vehicle for 4 weeks (23). LMT-28, a recently developed small-molecule inhibitor, has been shown to specifically target IL-6Rβ to disrupt its interaction with IL-6Rα, thus inhibiting IL-6 signaling (23). The efficacy of LMT-28 in inhibiting IL-6 signaling was confirmed by immunostaining for phosphorylated STAT3 (phospho-STAT3), an index of IL-6 signaling activation (23), in SupAos from mice, 4 weeks after Aldo-salt with LMT-28 or vehicle administration (Figure 5A). Importantly, compared with vehicle treatment, LMT-28 protected mice from Aldo-salt–induced suprarenal aortic dilation and progression (Figure 5, B and C), as well as from the development and severity of AAs (Figure 5, E−G). It is noteworthy, however, that LMT-28 did not affect the external diameter of the aorta (Figure 5D).

Interestingly, LMT-28 increased Aldo-salt–induced salt retention but did not affect serum sodium levels or MAP before or after Aldo-salt administration (Supplemental Figure 12, A−D, and Supplemental Figure 12G). Notably, we found no significant correlation between the internal diameter and serum sodium levels and MAP (Supplemental Figure 12, E and H). Additionally, there was no significant difference in serum sodium levels or MAP between mice treated or not with LMT-28, irrespective of whether they developed AAs (Supplemental Figure 12, F and I).

Aldo-salt may induce AAs through AR and androgen synthesis pathways. To explore this possibility, we determined AR protein expression by IHC and androgen synthesis (*Cyp17a1*, *Hsd3b2*, and *Hsd17b3*) (24) mRNA expression by real-time PCR in aortas, testes, and adrenal glands from 10-month-old male C57BL/6J mice, 10 days after Aldo-salt administration. The results revealed that Aldo-salt neither affected AR protein expression in the SupAo (Supplemental Figure 13, A and B) nor *Cyp17a1*, *Hsd3b2*, and *Hsd17b3* mRNA expression in the testis (Supplemental Figure 13, C−E). Interestingly, Aldo-salt moderately inhibited *Cyp17a1* and *Hsd17b3*, but not *Hsd3b2*, mRNA expression in the adrenal gland (Supplemental Figure 13, F−H). However, we observed no significant difference in plasma testosterone levels between mice that received Aldo-salt and those that did not (Supplemental Figure 13I).

### Identification of TCR and PD-1 as a link between androgen and Aldo-salt–induced AAs.

Since LMT-28 completely inhibited IL-6 signaling but only partially blocked Aldo-salt–induced AAs (Figure 5), we hypothesized that additional signaling pathways regulated by androgen might be involved in Aldo-salt–induced AAs. To identify these putative signaling ways in an unbiased way, 10-month-old male C57BL/6J mice were randomly divided into 3 treatment groups: (a) Aldo-salt; (b) orchiectomy followed by Aldo-salt; and (c) orchiectomy followed by Aldo-salt with DHT. Whole aortas were harvested 1 week after Aldo-salt administration. Subsequently, these samples were subjected to RNA-Seq for comprehensive gene expression analysis.

**Figure 6:**
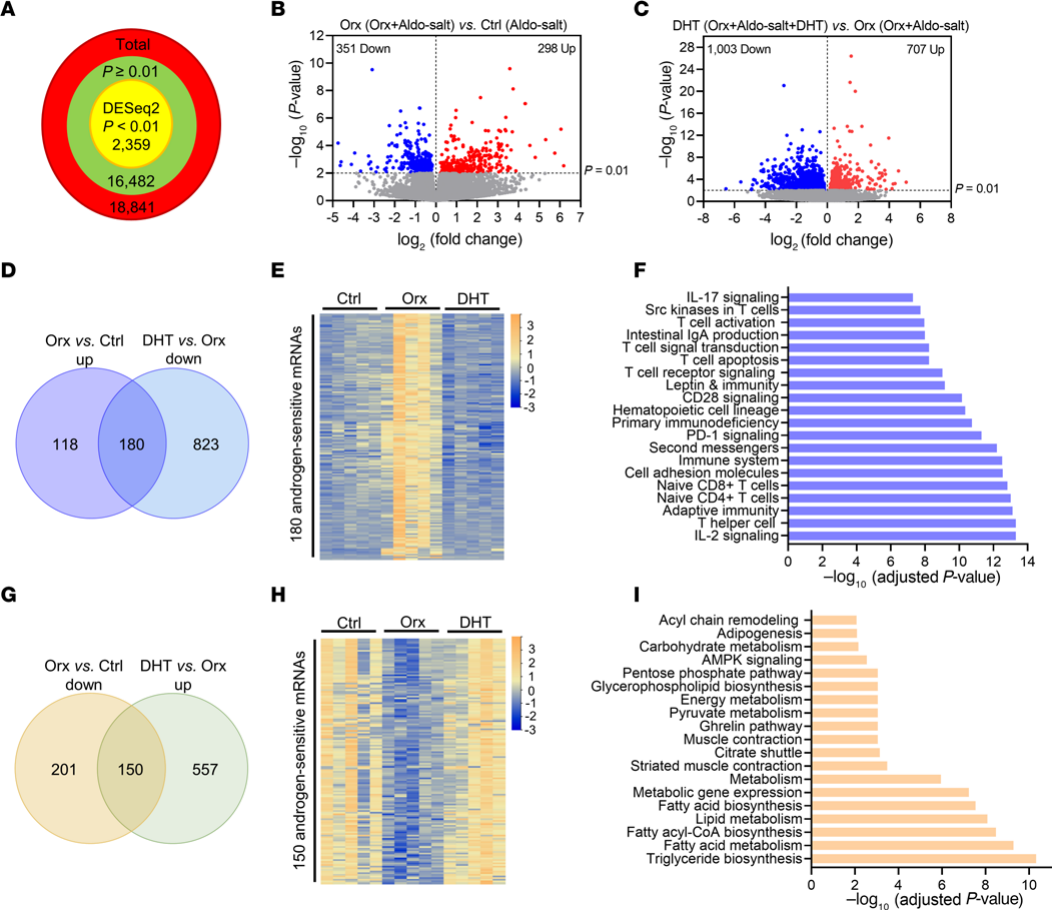
Profiling of aortic transcriptomes reveals TCR signaling as a link between the AR and Aldo-salt–induced AAs. (**A**) Total numbers of genes whose mRNAs were detected by RNA-Seq and determined by DESeq2 to be differentially abundant among whole aortas from 10-month-old C57BL/6J mice with and without orchiectomy followed by 1 week of Aldo-salt administration with and without DHT pellet implantation (*n* = 5/group). (**B** and **C**) Volcano plots of the number of genes whose mRNAs were determined by DESeq2 to be statistically significant (*y* axis) versus effect size (fold change, *x* axis) in the experiment. (**D** and **G**) Venn diagrams identify 180 genes whose mRNAs were upregulated (up) by orchiectomy but downregulated (down) by DHT and 150 genes whose mRNAs were downregulated by orchiectomy but upregulated by DHT, respectively. (**E** and **H**) Heatmaps of the 180 genes and 150 genes regulated by androgen. (**F** and **I**) Pathway enrichment analysis using Enrichr shows the top 20 pathways among mRNAs that were upregulated by orchiectomy but downregulated by DHT and the 19 pathways among the mRNAs that were downregulated by orchiectomy but upregulated by DHT, respectively.

Of a total of 18,841 mRNAs detected by RNA-Seq, DESeq2 (25) identified 2,359 that were significantly and differentially abundant (*P* < 0.01) among aortas from the 3 groups of mice (Figure 6A). Orchiectomy caused the upregulation of 298 mRNAs and the downregulation of 351 mRNAs (Figure 6B). Conversely, administration of DHT to orchiectomized mice resulted in the upregulation of 707 mRNAs and the downregulation of 1,003 mRNAs (Figure 6C). Importantly, the rescue of androgen deprivation by DHT in orchiectomized mice identified 180 androgen-sensitive mRNAs that were upregulated by orchiectomy but downregulated by DHT (Figure 6, D and E, and Supplemental Table 1) and 150 androgen-sensitive mRNAs that were downregulated by orchiectomy but upregulated by DHT (Figure 6, G and H, and Supplemental Table 2).

To gain mechanistic insight into the androgen-sensitive mRNAs identified by the RNA-Seq, we used Enrichr, a widely used search engine for comprehensive pathway enrichment analysis (26), to unveil the signaling pathways responsive to androgen and potentially implicated in Aldo-salt–induced AAs. On the basis of the 180 androgen-sensitive mRNAs upregulated by orchiectomy but downregulated by DHT (Figure 6, D and E), Enrichr analysis revealed 65 overrepresented functional annotations (Figure 6F and Supplemental Table 3). Surprisingly, most of these annotations were associated with adaptive immunity, particularly TCR signaling pathways, including PD-1 (Figure 6F and Supplemental Table 3). In parallel with this finding, the 150 androgen-sensitive mRNAs downregulated by orchiectomy but upregulated by DHT (Figure 6, G and H) were pinpointed to 19 overrepresented functional annotations, most of which were associated with triglyceride, fatty acid, and lipid biosynthesis or metabolism (Figure 6I and Supplemental Table 4).

Among the TCR signaling pathways revealed by RNA-Seq analysis, PD-1 is particularly interesting for several reasons. First, there is little information regarding the regulation of PD-1 by androgens and its role in AAs. Second, PD-1 is well recognized for its pivotal role as an immune checkpoint in regulating T cells, immunity, and immune-based cancer therapy (11, 27, 28). Third, the effects of PD-1 immune checkpoint therapy have been shown to differ between the sexes (29) and are associated with serious immune-related cardiovascular adverse events, including autoimmune myocarditis, pericarditis, and vasculitis (11, 27, 28). Consequently, it is conceivable that PD-1 may be involved in Aldo-salt–induced and androgen-mediated aortopathy. Therefore, our subsequent studies have been directed toward investigating PD-1 in greater depth.

RNA-Seq revealed 180 androgen-response genes associated with TCR and PD-1 signaling pathways. However, it is plausible that the findings may arise from the differential composition of aortic cells rather than the specific activation of TCR and PD-1 signaling pathways. To discern these possibilities, we conducted flow cytometric analysis of T cell subset signatures in aortas from 3 groups of 10-month-old male WT C57BL/6J mice 10 days after Aldo-salt and (a) sham operation, (b) orchiectomy, or (c) orchiectomy with DHT. As depicted in Supplemental Figures 14 and 15 for the gating strategy of flow cytometric analysis, single aortic cells were first gated on CD45 versus live/dead cell staining to select viable leukocytes and then gated on CD3, CD4, and CD8 to identify total T cells, CD4^+^ T cells, and CD8^+^ T cells, respectively. CD4^+^ and CD8^+^ T cells were further gated on CD44, CD62L, and CD127 to distinguish central memory T (Tcm) cells (CD44^+^CD62L^+^), naive T cells (CD44^–^CD62L^+^), effector T (Teff) cells (CD44^+^CD62L^–^CD127^–^), and effector memory T (Tem) cells (CD44^+^CD62L^–^CD127^+^), respectively. We selected these T cell subsets on the basis of our RNA-Seq pathway enrichment analysis (Figure 6F). These T cell subsets were also gated on PD-1 to pinpoint PD-1^+^ T cell subsets. As a control, a single-cell suspension from the spleen was analyzed by flow cytometry with fluorescence minus one (FMO) to define gating boundaries and ensure the specificity of the Abs.

**Figure 7:**
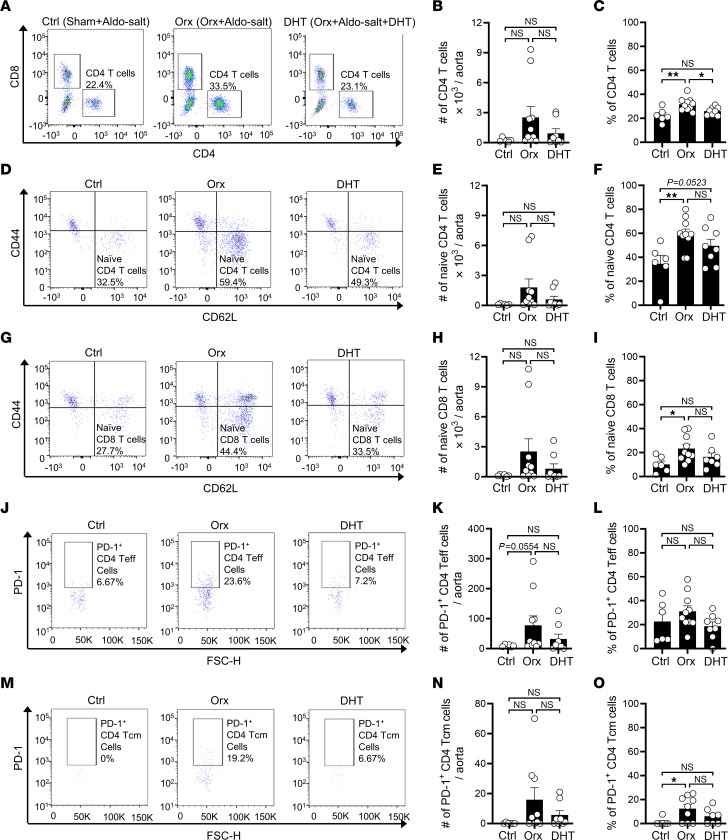
Flow cytometric analysis of T cell subsets in the aortas of orchiectomized and sham-operated mice 10 days after Aldo-salt, with and without DHT. Representative pseudocolor plots and quantitative data for the flow cytometric analysis of the total number and percentage of CD4^+^ T cells (CD45^+^CD3^+^CD4^+^; percentage of total T cells; **A**−**C**); naive CD4^+^ T cells (CD45^+^CD3^+^CD4^+^CD44^–^CD62L^+^; percentage of total CD4^+^ T cells; **D**−**F**); naive CD8^+^ T cells (CD45^+^CD3^+^CD8^+^CD44^–^CD62L^+^; percentage of total CD8^+^ T cells; **G**−**I**); PD-1^+^ effector CD4^+^ T cells (PD-1^+^CD4^+^ Teff cells; CD45^+^CD3^+^CD4^+^CD44^+^CD62L^–^CD127^–^PD-1^+^; percentage of total CD4^+^ Teff cells; **J**−**L**); and PD-1^+^ central memory CD4^+^ T cells (PD-1^+^CD4^+^ Tcm; CD45^+^CD3^+^CD4^+^CD44^+^CD62L^+^PD-1^+^; percentage of total CD4^+^ Tcm cells; **M**−**O**) in whole aortas of 9- to 10-month-old male C57BL/6J mice with orchiectomy or sham operation (Ctrl), 10 days after Aldo-salt administration with and without DHT pellet implantation (*n* = 6–10/group). Data are expressed as the mean ± SEM and were analyzed by 1-way ANOVA with multiple-comparison test. *P < 0.05 and **P < 0.01.

Concurrently with TCR and PD-1 signaling pathways identified by RNA-Seq analysis (Figure 6, D−F), the total numbers of all examined T cell subsets revealed a similar trend in response to orchiectomy and DHT: an increase with orchiectomy and a decrease with DHT (Supplemental Table 5). Consistent with these findings, several T cell subsets, including CD4^+^ T cells, naive CD4^+^ T cells, naive CD8^+^ T cells, PD-1^+^ CD4^+^ Teff cells, and PD-1^+^ CD4^+^ Tcm cells, displayed a significant or trending percentage increase induced by orchiectomy and/or a percentage decrease elicited by DHT (Figure 7 and Supplemental Table 5). Intriguingly, in contrast to the total T cell number response to orchiectomy and DHT, several other T cell subsets exhibited a significant or trending percentage decrease induced by orchiectomy and/or a percentage increase elicited by DHT (Supplemental Table 5).

To trace the origins of T cell subsets in the aorta, we analyzed T cell subsets by flow cytometry in spleens from the same 3 groups of mice. Interestingly, we found that the effect of androgen on T cell subsets in the spleens was similar to that in the aortas, although the total number, but not the percentage, of T cell subsets was mostly affected (Supplemental Figure 16 and Supplemental Table 6), indicating that alterations in T cell subsets within the spleen, induced by orchiectomy and/or DHT, contributed to the changes observed in T cell subsets in the aorta.

### Splenectomy mitigates Aldo-salt–induced AAs and augments PD-1^+^ T cells and PD-1^+^ B cells in the aorta.

**Figure 8:**
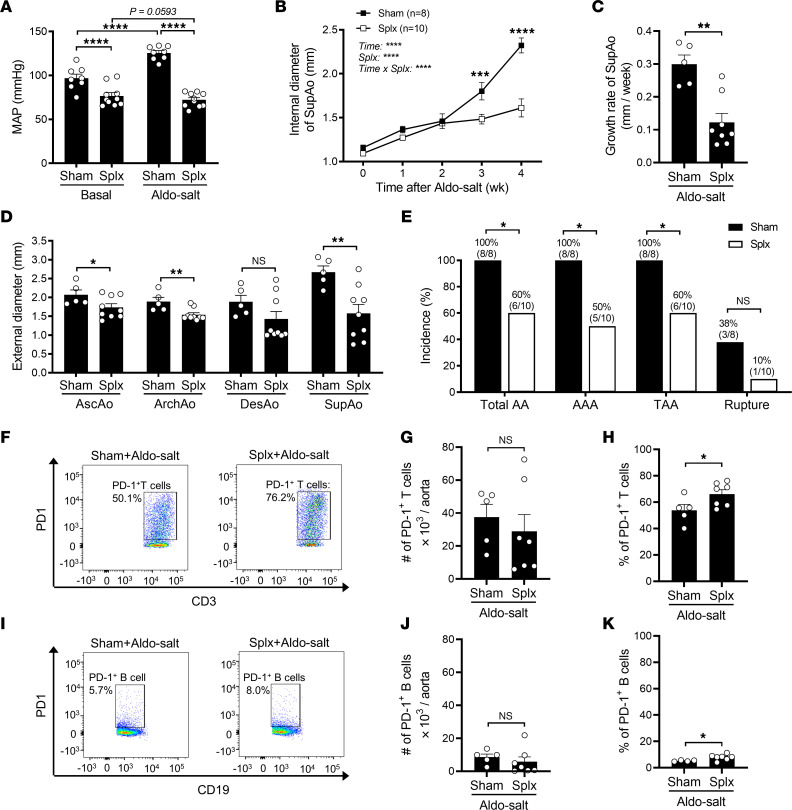
Splenectomy enriches PD-1^+^ T cells and B cells in the aorta and mitigates Aldo-salt–induced AAs. (**A**) MAP was measured by tail cuff in 11- to 13-month-old male C57BL/6J mice with splenectomy (Splx) or sham operation 1 week before (basal) and 3 weeks after Aldo-salt administration (*n* = 8–10/group). (**B** and **C**) Maximal internal diameters and growth rate of the SupAo (*n* = 8–10/group). ***P < 0.001 and ****P < 0.0001, vs. Splx at 3 and 4 weeks, respectively. (**D**) Maximal external diameters of the AscAo, ArchAo, DesAo, and SupAo (*n* = 5–9/group). (**E**) Incidence of total AAs, AAAs, TAAs, and aortic ruptures. (**F**−**K**) Representative pseudocolor plots and quantitative data for the flow cytometric analysis of the total numbers and percentages of PD-1^+^ T cells (CD45^+^CD3^+^PD-1^+^; percentage of total T cells) and PD-1^+^ B cells (CD45^+^CD19^+^PD-1^+^; percentage of total B cells) in whole aortas of mice with splenectomy or sham operation 4 weeks after Aldo-salt administration (*n* = 5–7/group). Data are expressed as the mean ± SEM and were analyzed by 2-way ANOVA with multiple-comparison test (**A** and **B**), 2-tailed, unpaired *t* test (**C**, **D**, **G**, **H**, **J**, and **K**), and 2-sided χ^2^ test (**E**). *P < 0.05, **P < 0.01, ***P < 0.001, and ****P < 0.0001.

To explore the potential involvement of T cells in Aldo-salt–induced AAs, 11- to 13-month-old male C57BL/6J mice were subjected to splenectomy or sham operation, and 4 weeks later, they were given Aldo-salt for an additional 4 weeks. In line with previous findings (30), splenectomy lowered the MAP before and after Aldo-salt administration (Figure 8A), indicating the effectiveness of splenectomy. Compared with sham operations, splenectomy suppressed Aldo-salt–induced suprarenal aortic dilation and progression (Figure 8, B and C), the external diameters of the AscAo, ArchAo, and SupAo (Figure 8D), and the incidence of AAAs, TAAs, and aortic ruptures (Figure 8E). Interestingly, we noted a significant correlation between MAP and the internal diameters of the SupAo of mice 3 weeks after Aldo-salt administration (Supplemental Figure 17A). However, there was no significant difference in MAP between splenectomized mice with and without AAs (Supplemental Figure 17B).

To investigate whether PD-1^+^ T cells are implicated in the effect of splenectomy on Aldo-salt–induced AAs, we conducted flow cytometric analysis of the aortas from splenectomized and sham-operated mice 4 weeks after Aldo-salt administration. As delineated in Supplemental Figure 18, single aortic cells were first gated on CD45 to sort leukocytes and then gated on CD3, CD19, F4/80, and Ly6G to identify T cells, B cells, macrophages, and neutrophils, respectively. These cells were further gated on PD-1 to identify PD-1^+^ T cells, PD-1^+^ B cells, PD-1^+^ macrophages, and PD-1^+^ neutrophils. Compared with sham operation, splenectomy did not affect the total number or percentage of leukocytes, T cells, B cells, macrophages, or neutrophils in the aorta of mice administered Aldo-salt (Supplemental Figure 19). However, splenectomy notably increased the percentage, albeit not the total number, of PD-1^+^ T cells and PD-1^+^ B cells in the aorta of mice administered Aldo-salt (Figure 8, F−K). Conversely, splenectomy significantly decreased the percentage and total number of PD-1^+^ neutrophils, but not PD-1^+^ macrophages, in the aorta of mice administered Aldo-salt (Supplemental Figure 20, A−F).

To trace the origins of PD-1^+^ T cell subsets in the aorta of splenectomized mice, we performed flow cytometric analysis of the blood and periaortic lymph nodes in 11- to 13-month-old splenectomized and sham-operated mice 4 weeks after Aldo-salt administration. Interestingly, we found that splenectomy did not alter the total number or percentage of leukocytes, T cells, B cells, PD-1^+^ T cells, or PD-1^+^ B cells in the blood compared with sham operation (Supplemental Figure 21). In contrast, splenectomy led to a notable increase in the total number, although not the percentage, of leukocytes, T cells, B cells, PD-1^+^ T cells, and PD-1^+^ B cells in the periaortic lymph nodes relative to sham operation (Supplemental Figure 22). These findings suggest that splenectomy may enrich PD-1^+^ T cells and PD-1^+^ B cells in the aortas via the periaortic lymph nodes.

### The AR binds to the PD-1 promoter and suppresses PD-1 mRNA and protein expression in the spleen.

**Figure 9:**
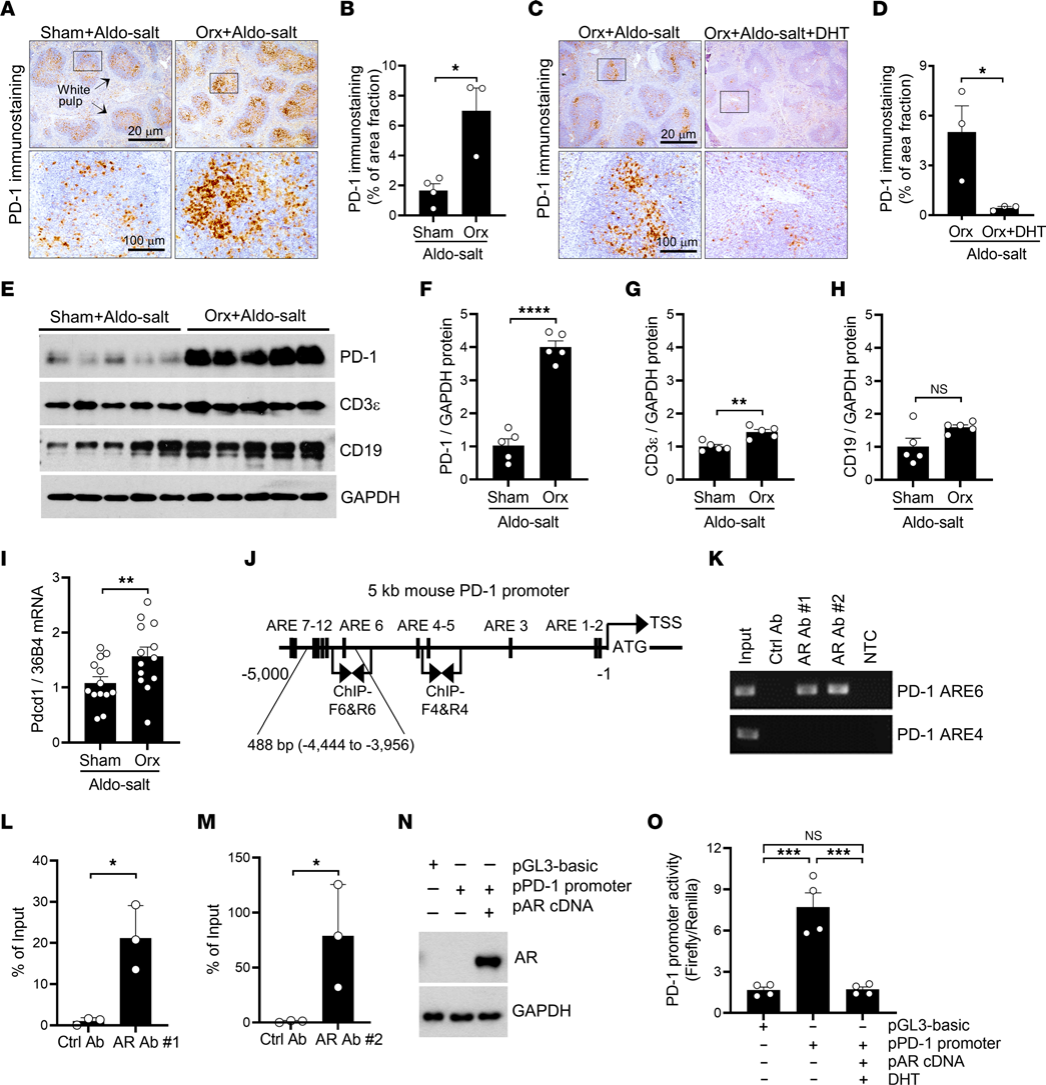
Androgen suppresses PD-1 mRNA and protein expression in the spleen in mice administered Aldo-salt. (**A**−**D**) Representative immunostainings and quantitative data for PD-1 protein expression in spleens from 10-month-old male C57BL/6J mice with orchiectomy or sham operation 10 days after Aldo-salt administration (*n* = 3–4/group) or from orchiectomized mice 4 weeks after Aldo-salt administration with and without DHT pellet implantation (*n* = 3/group). Percentage of areas fraction = (PD-1^+^ area/area of fields of view) × 100%. The data were calculated from 5 fields of view randomly photographed per splenic section per mouse. (**E**−**H**) Representative Western blots and quantitative data for PD-1, CD3ε, CD19, and GAPDH protein expression in spleens from 10-month-old male mice with orchiectomy or sham operation 10 days after Aldo-salt administration (*n* = 5/group). (**I**) *Pdcd1* (the gene encodes PD-1) mRNA expression was normalized to 36B4 (a housekeeping gene, also called Rplp0 [ribosomal protein lateral stalk subunit P0]) in spleens from 10-month-old male mice with orchiectomy or sham operation 10 days after Aldo-salt administration (*n* = 13/group). (**J**) Schematic diagram of the 12 AREs in the 5 kb mouse PD-1 promoter. ATG, translation start codon. ChIP-F, ChIP PCR forward primers; ChIP-R, ChIP PCR reverse primers. (**K**−**M**) Representative and quantitative ChIP-PCR data for the control Ab, anti-AR Ab 1, and anti-AR Ab 2 in the spleen (*n* = 3/group). NTC, no template control. (**N** and **O**) AR expression in HEK293 cells suppressed PD-1 promoter activity (*n* = 4/group). Data are expressed as the mean ± SEM and were analyzed by 2-tailed, unpaired *t* test (**B**, **D**, **F**−**H**, **I**, **L**, and **M**) and 1-way ANOVA with multiple-comparison test (**O**). *P < 0.05, **P < 0.01, ***P < 0.001, and ****P < 0.0001. Scale bars: 20 μm and 100 μm (enlarged insets).

We conducted a series of experiments to determine whether PD-1 is regulated by androgen in the spleen. First, we examined PD-1 protein expression by IHC in spleens from 10-month-old male C57BL/6J mice that underwent orchiectomy or sham operation 10 days after Aldo-salt administration. PD-1 protein was predominantly observed in the white pulp of the spleen (Figure 9, A and B), a region primarily composed of T cells and B cells (31). Importantly, PD-1 protein expression was notably elevated by orchiectomy in the spleen relative to sham operation (Figure 9, A and B). Consistent with these findings, DHT administration to orchiectomized mice abolished PD-1 protein upregulation in the spleen 4 weeks after Aldo-salt administration (Figure 9, C and D).

Next, we quantified PD-1 protein expression by Western blotting in the spleens of 10-month-old orchiectomized or sham-operated C57BL/6J mice 10 days after Aldo-salt administration. We found that PD-1 protein expression was markedly upregulated by up to 4-fold following orchiectomy in the spleen compared with sham operation (Figure 9, E and F). To discern whether orchiectomy-induced PD-1 protein upregulation in the spleen was attributable to T cells or B cells, we examined protein expression of CD3ε, a T cell marker, and CD19, a B cell marker, in the same spleen lysate. Interestingly, expression of both CD3ε and CD19 proteins was moderately increased in the spleens of orchiectomized mice compared with sham-operated mice, but only CD3ε protein upregulation was statistically significant (Figure 9, E, G, and H).

We also investigated the effects of orchiectomy on PD-1, CD3ε, and CD19 protein expression in the spleens of 10-month-old orchiectomized and sham-operated C57BL/6J mice without Aldo-salt administration. An increasing trend in basal protein expression of PD-1, but not CD3ε or CD19, was observed in the spleens (Supplemental Figure 23). However, the level of PD-1 upregulation induced by orchiectomy in the spleens of mice without Aldo-salt treatment was notably lower than in mice with Aldo-salt (compared Supplemental Figure 23 with Figure 9, E and F).

We then conducted flow cytometric analysis of spleens from 10-month-old orchiectomized and sham-operated C57BL/6J mice 10 days after Aldo-salt administration to discern the upregulation of PD-1 protein, as detected by IHC and Western blotting, in splenic T cells and B cells. Interestingly, we observed that orchiectomy significantly increased the total number, but not the percentage, of splenic PD-1^+^ T cells, but not splenic PD-1^+^ B cells, compared with sham operation 10 days after Aldo-salt administration (Supplemental Figure 24). These findings suggest that orchiectomy-induced PD-1 protein upregulation in the spleen mainly resulted from splenic PD-1^+^ T cells, rather than splenic PD-1^+^ B cells, in mice administered Aldo-salt.

To investigate whether splenic PD-1 is regulated by androgen at the transcription level, we determined *Pdcd1* (the gene that codes PD-1) mRNA expression by real-time PCR in the spleens of 10-month-old male C57BL/6J mice with orchiectomy or sham operation 10 days after Aldo-salt administration. We found that *Pdcd*1 mRNA was significantly upregulated by orchiectomy in the spleen compared with sham operation (Figure 9I).

Next, to investigate the mechanism by which androgen suppresses *Pdcd1* mRNA expression in the spleen in mice administered Aldo-salt, we examined whether the AR binds to the PD-1 promoter to suppress PD-1 transcription. We analyzed a 5 kb mouse PD-1 promoter DNA sequence to identify androgen response elements (AREs) containing AGAACA or TGTTCT hexamers, which are known to bind the AR effectively (32). We found 12 putative AREs in the 5 kb mouse PD-1 promoter (Figure 9J). To determine whether the AR can bind to these putative AREs in the spleen, we performed a ChIP assay using mouse spleen samples with 2 commercially available ChIP-grade anti-AR Abs with distinct epitopes, along with 2 sets of ChIP-PCR primers specific for amplifying ARE4 and ARE6 in the mouse PD-1 promoter (Figure 9J). Both anti-AR Abs, but not the control Ab, successfully pulled down the chromatin fragments containing ARE6 but not ARE4 (Figure 9, K−M), indicating that the AR can bind to ARE6 but not ARE4 in the mouse PD-1 promoter in the spleen.

As a next step, to investigate whether binding of the AR to the PD-1 promoter inhibits its transcriptional activity, we subcloned a 488 bp PD-1 mouse promoter (–4,444 to –3,956 bp relative to the transcription start site [TSS]) containing ARE6 to ARE10 (Figure 9J) into a pGL3-basic firefly luciferase report vector. The pGL3-basic–PD-1 promoter construct was cotransfected with the pRL-TK control vector and a pcDNA Flag-M4-AR construct (33) into HEK293 cells. Dual luciferase assays revealed that the 488 bp PD-1 promoter had a 4.6-fold higher luciferase activity than did the pGL3-basic vector (Figure 9O), indicating that the subcloned 488 bp PD-1 promoter can drive PD-1 transcription. Importantly, cotransfection of the PD-1 promoter–luciferase constructs with the human AR cDNA construct completely abolished PD-1 promoter activity in the presence of DHT (Figure 9, N and O).

Next, to investigate the link between orchiectomy-induced PD-1 upregulation in the spleen and Aldo-salt–induced AAs, we conducted flow cytometric analysis of the blood of 10-month-old orchiectomized and sham-operated C57BL/6J mice 10 days after Aldo-salt administration. In line with its effect on PD-1^+^ T cells and PD-1^+^ B cells in the spleen (Supplemental Figure 24), orchiectomy amplified both the total number and percentage of PD-1^+^ T cells in the blood, although it did not affect PD-1^+^ B cells (Supplemental Figure 25).

To investigate whether orchiectomy-induced PD-1 upregulation also occurs in other immune organs, we examined PD-1 protein expression by IHC and Western blot analysis in the periaortic lymph nodes of 10-month-old orchiectomized and sham-operated C57BL/6J mice 10 days after Aldo-salt administration. IHC analysis showed a discernible increasing trend in PD-1 immunostaining in the periaortic lymph nodes of orchiectomized mice compared with sham-operated mice (Supplemental Figure 26, A and B). This observation was further supported by Western blot analysis (Supplemental Figure 26, C and D).

Finally, to ascertain whether PD-1 regulates IL-6 in T cells, we assessed IL-6 mRNA and protein expression in the spleens of 4-month-old male global PD-1–KO (34) and WT C57BL/6J mice that had received 8 weeks of HFD feeding and 4 weeks of Ang II infusion (35). We found no significant differences in IL-6 mRNA or protein expression in the spleen between PD-1–KO and WT mice (Supplemental Figure 27, A−E). Consistent with these findings, there was no significant difference in serum IL-6 protein levels between PD-1–KO and WT mice (Supplemental Figure 27F).

### Blockade of the immune checkpoint with anti–PD-1 Ab reinstates Aldo-salt–induced AAs in orchiectomized mice.

**Figure 10:**
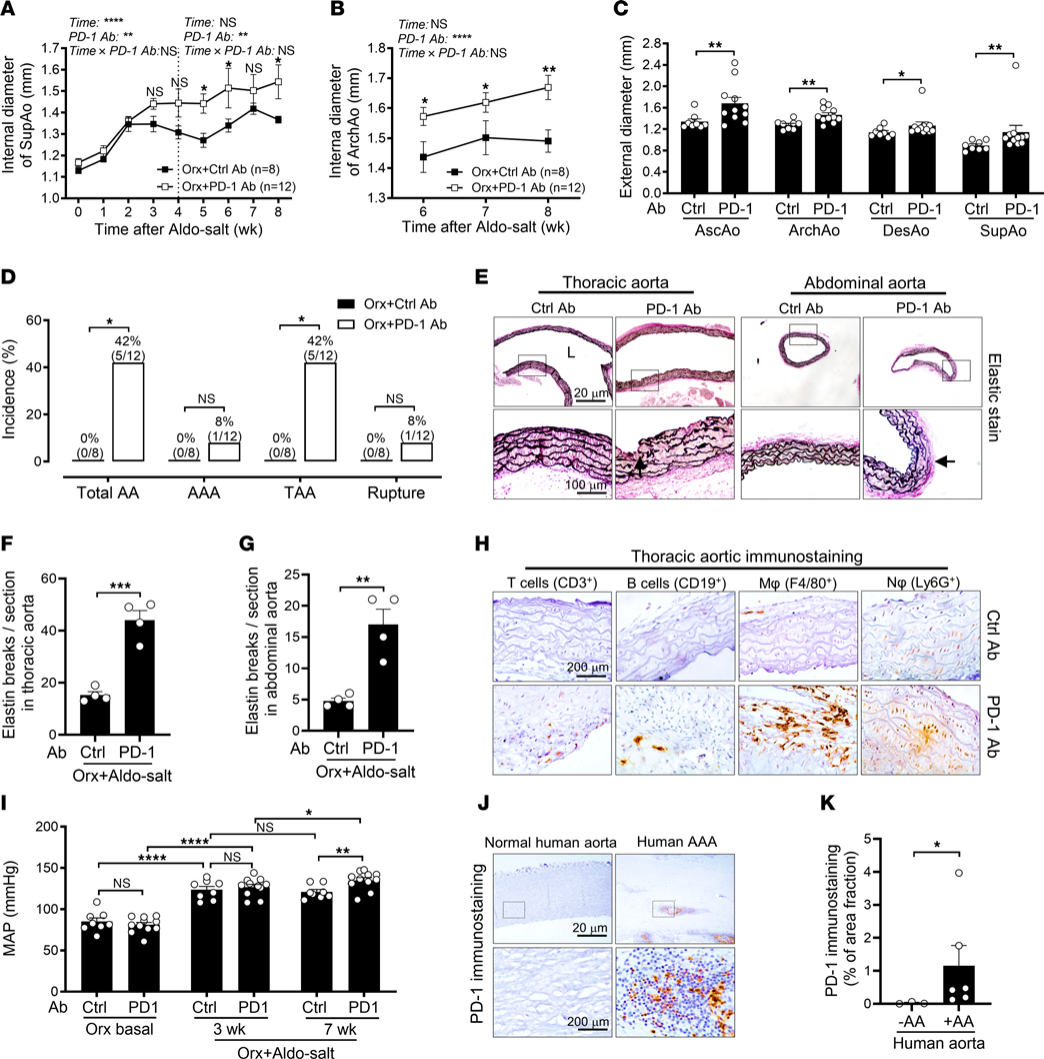
Intraperitoneal injection of anti–PD-1 Ab reinstates Aldo-salt–induced aortopathy in orchiectomized mice. (**A** and **B**) Maximal internal diameters of the SupAo and ArchAo of 10-month-old male orchiectomized C57BL/6J mice before and after Aldo-salt with anti–PD-1 or control Ab injection (*n* = 8–12/group). *P < 0.05 and **P < 0.01, vs. Orx+Ctrl Ab at 5, 6, 7, and 8 weeks, respectively. (**C**) Maximal external diameters of the AscAo, ArchAo, DesAo, and SupAo (*n* = 8–11/group). (**D**) Incidence of total AAs, AAAs, TAAs, and aortic ruptures. (**E**−**G**) Representative and quantitative Verhoeff-Van Gieson staining of elastin in longitudinal sections of the thoracic aortas and cross-sections of the abdominal aortas in orchiectomized mice with anti–PD-1 or control Ab 8 weeks after Aldo-salt administration (*n* = 4/group). Arrow indicates elastic breakage. Scale bars: 20 μm and 100 μm (enlarged insets). (**H**) Representative immunostainings of T cells, B cells, macrophages, and neutrophils in the thoracic aortas in orchiectomized mice 8 weeks after Aldo-salt with anti–PD-1 or control Ab administration (*n* = 3). Scale bar: 200 μm. (**I**) MAP of orchiectomized mice 1 week before (basal) and 3 weeks and 7 weeks after Aldo-salt with anti–PD-1 or control Ab administration (*n* = 8–11/group). *P < 0.05 and **P < 0.01, vs. Orx+Ctrl Ab at 5, 6, 7, and 8 weeks, respectively. (**J** and **K**) Representative immunostainings and quantitative data for PD-1 protein expression in human aortas with and without AAs (*n* = 3–6/group). Scale bars: 20 μm and 200 μm (enlarged insets). Data are expressed as the mean ± SEM and were analyzed by 2-way ANOVA with multiple-comparison test (**A**, **B**, and **I**), 2-tailed, unpaired *t* test (**C**, **F**, **G**, and **K**), and 2-sided χ^2^ test (**D**). *P < 0.05, **P < 0.01, ***P < 0.001, and ****P < 0.0001.

To explore the potential role of PD-1 in Aldo-salt–induced and androgen-mediated AAs, 10-month-old C57BL/6J male mice were orchiectomized and then administered Aldo-salt with a specific rat anti–mouse PD-1 Ab or an isotype control Ab (200 mg/mice, i.p. injection, twice a week) for 8 weeks (36). Compared with the control Ab, anti–PD-1 Ab significantly enhanced suprarenal aortic dilation from week 4 to week 8 (Figure 10A). A similar but more potent effect of anti–PD-1 Ab was found on Aldo-salt–induced ArchAo dilation from week 6 to week 8 after Aldo-salt with anti–PD-1 or control Ab administration (Figure 10B). Additionally, anti–PD-1 Ab significantly increased the external diameters of the AscAo, ArchAo, DesAo, and SupAo relative to the control Ab (Figure 10C). Moreover, of the 12 mice that received anti–PD-1 Ab, 5 developed AAs (45%), including 1 AAA (8%), 5 TAAs (45%), and 1 aortic ruptures (8%). In contrast, none of 8 mice with the control Ab developed AAs (Figure 10D).

The aortas were harvested from orchiectomized mice 8 weeks after Aldo-salt with anti–PD-1 or control Ab administration and then subjected to Verhoeff-Van Gieson staining (9, 10) to assess the effect of anti–PD-1 Ab on Aldo-salt–induced aortic elastin fiber fragmentation. A noticeable increase in the breakage of thoracic and abdominal aortic elastin fiber was evident in orchiectomized mice with AAs induced by anti–PD-1 Ab, but not in mice without AAs that were administered the control Ab (Figure 10, E−G). IHC was performed on the same thoracic aortas using anti-CD3ε, anti-CD19, anti-F4/80, and anti-Ly6G Abs to identify T cells, B cells, macrophages, and neutrophils, respectively. As depicted in Figure 10H, T cells, B cells, macrophages, and neutrophils were prominently present in the thoracic aorta of mice with TAA induced by anti–PD-1 Ab, but they were barely detectable in mice without a TAA administered the control Ab.

Interestingly, anti–PD-1 Ab did not affect MAP before or 3 weeks after Aldo-salt administration, but it exacerbated Aldo-salt–induced hypertension 7 weeks after Aldo-salt administration (Figure 10I). We observed a significant correlation between the internal diameters of the ArchAo and MAP seven weeks after Aldo-salt administration (Supplemental Figure 28A). However, there was no significant difference in MAP between orchiectomized mice treated with the anti–PD-1 Ab, regardless of whether they developed AAs (Supplemental Figure 28B).

To explore the potential involvement of PD-1 in human AAs, we examined PD-1 protein expression by IHC in the abdominal aorta specimens from patients with or without AAAs. We found that PD-1 protein was scarcely detectable in normal abdominal aortas but was readily found in human AAAs (Figure 10, J and K).

### Adoptive PD-1–deficient T cell transfer resumes Aldo-salt–induced aortopathy in orchiectomized mice.

To further define the role of PD-1 in the pathogenesis of Aldo-salt–induced AAs, PD-1–deficient T cells and WT T cells were isolated by microbeads conjugated with a monoclonal anti–mouse CD90.2 Ab from the spleens of 4- to 5-month-old male PD-1–KO and WT C57BL6J donor mice and then adoptively transferred into 9- to 10-month-old orchiectomized C57BL/6J recipient mice via retro-orbital sinus injection 2 days before and 8 and 18 days after Aldo-salt administration. Pilot experiments confirmed the presence of adoptively transferred T cells preloaded with a red fluorescent cell tracker in the recipients’ spleens and aortas (our unpublished observation).

**Figure 11:**
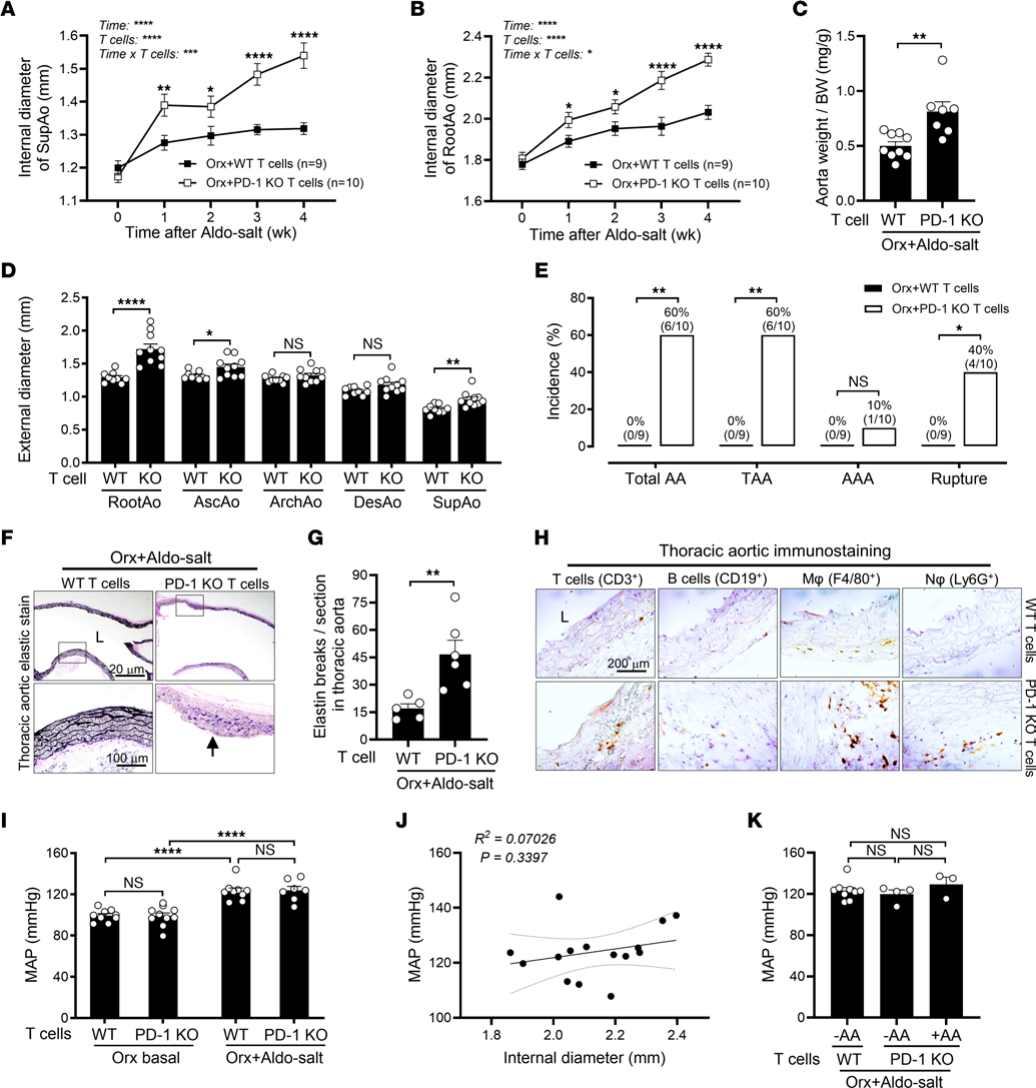
Adoptive PD-1–deficient T cell transfer restores Aldo-salt–induced aortopathy in orchiectomized mice. (**A** and **B**) Maximal internal diameters of the SupAo and aortic root (RootAo) in 9- to 10-month-old orchiectomized male C57BL/6J mice with adoptive PD-1–KO and WT T cell transfer via retro-orbital sinus injection 2 days before and 8 days and 18 days after Aldo-salt administration (*n* = 9–10/group). PD-1–KO and WT T cells were isolated from the spleens of 4-month-old male PD-1–KO and WT C57BL6J mice via anti-CD90.2 magnetic beads. *P < 0.05, **P < 0.01, and ****P < 0.0001, vs. Orx+WT T cells at 1, 2, 3, and 4 weeks, respectively. (**C**) Aorta weight/BW ratio (*n* = 7–9/group). (**D**) Maximal external diameters of the RootAo, AscAo, ArchAo, DesAo, and SupAo (*n* = 9–10/group). (**E**) Incidence of total AAs, AAAs, TAAs, and aortic ruptures. (**F** and **G**) Representative elastin staining images and quantitative data for longitudinal sections of thoracic aortas (*n* = 5–6/group). Arrow indicates elastic breakage. Scale bars: 20 μm and 100 μm (enlarged insets). (**H**) Representative immunostaining of T cells, B cells, macrophages (Mφ), and neutrophils (Nφ) in thoracic aortas (*n* = 3). Scale bar: 200 μm. (**I**) MAP was measured by tail cuff 1 week before (basal) and 3 weeks after Aldo-salt administration (*n* = 8–10/group). *P < 0.05, **P < 0.01, and ****P < 0.0001, vs. Orx+WT T cells at 1, 2, 3, and 4 weeks, respectively. (**J**) Correlation analysis of the internal diameter of the RootAo and MAP 3 weeks after Aldo-salt administration (*n* = 15/group). (**K**) MAP in mice with (+) and without (–) Aldo-salt–induced AAs (*n* = 3–9/group). Data are expressed as the mean ± SEM and were analyzed by 2-way ANOVA with multiple-comparison test (**A**, **B**, and **I**), 2-tailed, unpaired *t* test (**C**, **D**, and **G**), 2-sided χ^2^ test (**E**), simple linear regression analysis (**J**), and 1-way ANOVA for multiple comparisons (**K**). *P < 0.05, **P < 0.01, ***P < 0.001, and ****P < 0.0001.

Compared with mice that received WT T cells, those with adoptive PD-1–deficient T cell transfer displayed significantly greater suprarenal aortic and aortic root (RootAo) dilation following Aldo-salt administration (Figure 11, A and B). Consistent with these findings, the aorta weight/BW ratio, an index of AA severity (5), but not the spleen weight/BW ratio, was also significantly increased in mice with adoptive PD-1–deficient T cell transfer compared with those with adoptive WT T cell transfer (Figure 11C and Supplemental Figure 29). Additionally, adoptive PD-1–deficient T cell transfer, relative to adoptive WT T cell transfer, led to a significant increase in the external diameters of the RootAo, AscAo, and SupAo 4 weeks after Aldo-salt administration (Figure 11D), along with a 60% incidence of TAAs, 10% of AAAs, and 40% of aortic ruptures (Figure 11E). In contrast, none of the mice that received adoptive WT T cell transfer developed TAAs, AAAs, or aortic ruptures.

Mice that received PD-1–deficient T cells developed TAAs, characterized by evident elastin fiber breakages (Figure 11, F and G) and prominent infiltration of T cells, macrophages, and neutrophils, but not B cells, into the thoracic aorta compared with those with WT T cell transfer without TAAs (Figure 11H). However, adoptive PD-1–deficient T cell transfer did not affect MAP before or after Aldo-salt administration (Figure 11I). We found no significant correlation between MAP and aortic root dilation in mice receiving PD-1–deficient or WT T cell transfer 3 weeks after Aldo-salt administration (Figure 11J). Furthermore, there was no significant difference in MAP between mice with PD-1–deficient T cell transfer and those with WT T cell transfer, regardless of whether these mice had AAs (Figure 11K).

### Genetic deletion of PD-1 exacerbates HFD- and Ang II–induced AAA in nonorchiectomized mice.

**Figure 12:**
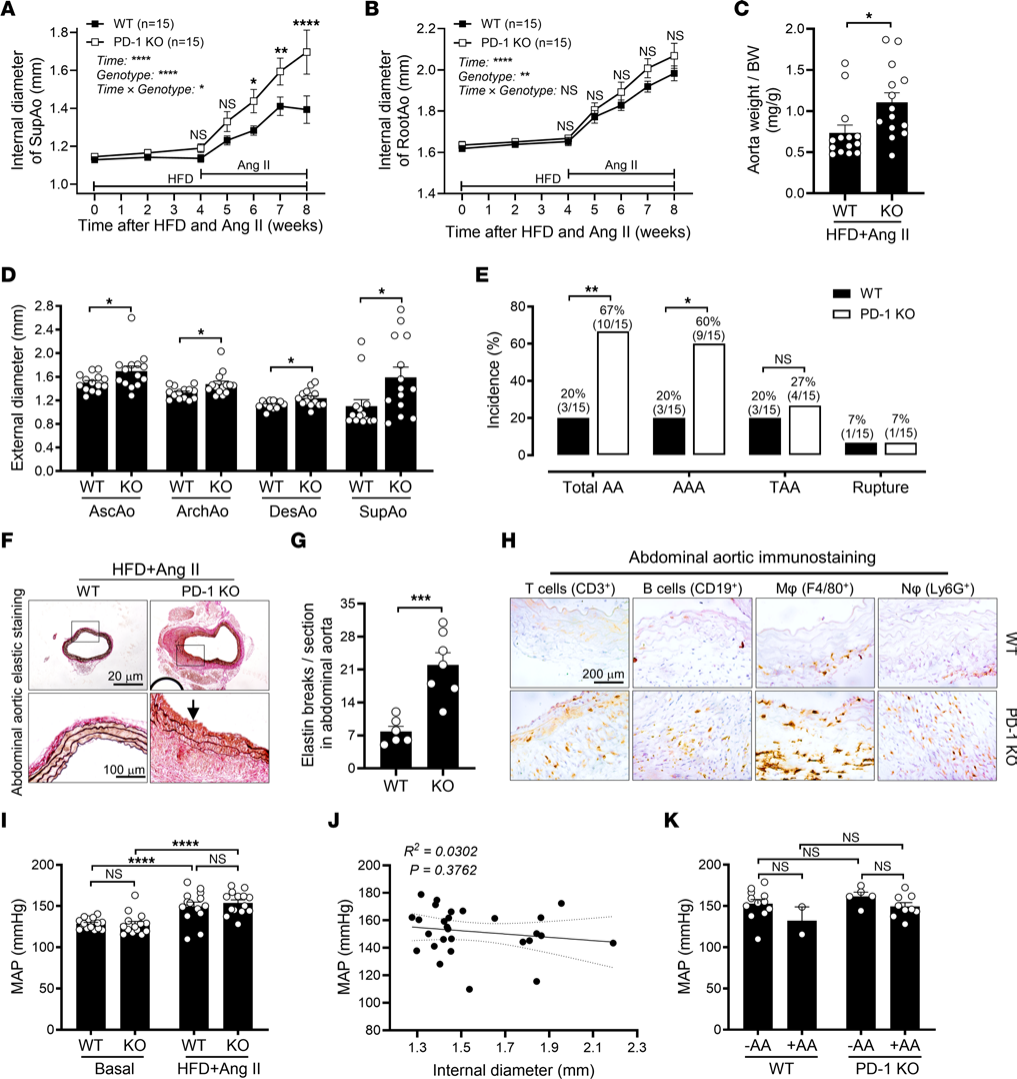
Genetic deletion of PD-1 exacerbates HFD- and Ang II–induced aortopathy. (**A** and **B**) Maximal internal diameters of the SupAo and aortic root in 2-month-old male PD-1–KO and WT C57BL/6J mice with 8-week HFD feeding and 4-week Ang II infusion (*n* = 15/group). *P < 0.05, **P < 0.01, and ****P < 0.0001, vs. WT at 6, 7, and 8 weeks, respectively. (**C**) Aortic weight/BW ratio (*n* = 14/group). (**D**) Maximal external diameters of the AscAo, ArchAo, DesAo, and SupAo (*n* = 14/group). (**E**) Incidence of total AAs, AAAs, TAAs, and aortic ruptures. (**F** and **G**) Representative Verhoeff-Van Gieson elastin staining of abdominal aortas and quantitative data. Arrow indicates elastic breakage (*n* = 6–7/group). Scale bars: 20 μm and 100 μm (enlarged insets). (**H**) Representative immunostaining of T cells, B cells, macrophages, and neutrophils in the abdominal aorta (*n* = 3). (**I**) MAP was measured by tail cuff 1 week before (basal) and 3 weeks after HFD and Ang II administration (*n* = 14–15/group). Scale bar: 200 μm. (**J**) Correlation analysis of the internal diameter of the SupAo and MAP in PD-1–KO and WT mice 3 weeks after HFD and Ang II administration (*n* = 28/group). (**K**) MAP with (+) and without (–) AAs (*n* =2–12/group). Data are expressed as the mean ± SEM and were analyzed by 2-way ANOVA with multiple-comparison test (**A**, **B**, **I**, and **K**), 2-tailed, unpaired *t* test (**C**, **D**, and **G**), 2-sided χ^2^ test (**E**), and simple linear regression analysis (**J**). *P < 0.05, **P < 0.01, ***P < 0.001, and ****P < 0.0001.

To investigate whether PD-1 is implicated in other AA animal models, 2-month-old male PD-1–KO and WT C57BL/6J mice (34) were fed a HFD for 1 month and then infused with Ang II in the continued presence of HFD feeding for an additional month to induce AAA (35). A HFD and Ang II, but not a HFD alone, resulted in both abdominal and thoracic aortic dilation, and, importantly, genetic deletion of PD-1 exacerbated HFD- and Ang II–induced aortic dilation, with a more pronounced effect observed in the SupAo than in the RootAo (Figure 12, A and B). In line with these findings, PD-1–KO mice also exhibited a significant increase in the aorta weight/BW ratio (Figure 12C and Supplemental Figure 30A), the external diameters of the AscAo, ArchAo, DesAo, and SupAo (Figure 12D), and the incidence of AAs, mainly AAAs rather than TAAs, which were different from those in orchiectomized mice treated with Aldo-salt (Figure 12E vs. Figure 10D and Figure 11E).

Interestingly, genetic deletion of PD-1 did not affect BW 4 weeks after HFD feeding or HFD feeding plus Ang II infusion (Supplemental Figure 30B). However, genetic deletion of PD-1 significantly increased the spleen weight and the spleen weight/BW ratio, but not the kidney weight or the kidney weight/BW ratio (Supplemental Figure 30, C−F), indicating the involvement of immune cells. Consistent with these findings, genetic deletion of PD-1 amplified HFD- and Ang II–induced elastin fiber fragmentation and infiltration of T cells, B cells, macrophages, and neutrophils in the SupAo compared with WT mice (Figure 12, F−H).

Genetic deletion of PD-1 did not affect MAP before or after HFD feeding and Ang II infusion (Figure 12I). There was no significant correlation between MAP and the internal diameters of the SupAo in PD-1–KO and WT mice 3 weeks after HFD and Ang II administration (Figure 12J). Furthermore, there was no significant difference in MAP between PD-1–KO and WT mice, regardless of whether they developed AAAs (Figure 12K).

## Discussion

It has long been recognized that androgen plays a role in cardiovascular diseases (37). However, whether androgen protects or aggravates AAs remains inconclusive and appears to be animal model specific (5–7, 16, 38). In this study, we report that Aldo-salt–induced AAs mimicked human AAA (3) and mostly occurred in male mice (Figure 1). Consistent with the Ang II and elastase AAA mouse models (5, 7, 16). but not the Ang II plus CaCl_2_ mouse model (38), we demonstrate that Aldo-salt–induced AAs were abolished or ameliorated by global androgen deprivation via orchiectomy (Figure 2), downregulation of the AR with ASC-J9 (Figure 4), and inhibition of the AR with flutamide (Supplemental Figure 8). Importantly, restoration of androgen in orchiectomized mice reinstated Aldo-salt–induced AAs (Figure 3).

One of the most important findings is that androgen aggravated Aldo-salt–induced AAs, at least partially, by suppressing PD-1^+^ T cells in the spleen. Several lines of evidence support this potential mechanism. First, RNA-Seq identified 180 genes upregulated by orchiectomy but downregulated by DHT in the aortas of mice 1 week after Aldo-salt administration (Figure 6, D and E, and Supplemental Table 1). Surprisingly, these 180 androgen-sensitive genes were mostly mapped to TCR signaling, including signaling with PD-1 (Figure 6F and Supplemental Table 3), and, importantly, these findings were largely confirmed by flow cytometry (Figure 7 and Supplemental Table 5). Second, consistent with the potential role of T cells in AAAs (39–41), splenectomy mitigated Aldo-salt–induced AAs (Figure 8, A−E) and was accompanied by the enrichment of PD-1^+^ T cells and PD-1^+^ B cells in the aorta of mice administered Aldo-salt, probably via the periaortic lymph nodes (Figure 8, F−K, and Supplemental Figures 18−22). Third, orchiectomy potently augmented PD-1^+^ T cells, but not PD-1^+^ B cells, in the spleen, blood, and lymph nodes of mice administered Aldo-salt (Figure 9, A−H, and Supplemental Figures 24−26). Finally, immune checkpoint blockade with anti–PD-1 Ab, adoptive PD-1–deficient T cell transfers, and genetic deletion of PD-1 reinstated or exacerbated Aldo-salt–induced or HFD- and Ang II–induced aortopathies, including elastin degradation, vascular inflammation, TAA, and AAA in intact and orchiectomized mice (Figures 10−12). These findings align well with the established role of PD-1 as an immune checkpoint implicated in various diseases, including giant cell arteritis, cancer, and atherosclerosis (28). However, it should be noted that these results contradict a recent study in which humanized PD-1 Ab mitigated rather than aggravated AAA in a CaCl_2_ mouse model and an aortic patch angioplasty rat model (42). The discrepancy between these studies may be attributed to differences in animal models, animal age, and anti–PD-1 Ab. Further studies are needed to investigate these possibilities.

PD-1 functions as an immune checkpoint, inhibiting T cell activation via interaction with its ligand, primarily PD-L1 (28). PD-1 is exclusively expressed in activated immune cells, most importantly in T cells, whereas PD-L1 is broadly expressed in various cells, including antigen-presenting cells (i.e., macrophages), cancer cells, and endothelial cells (28). It is well documented that PD-1 is upregulated by estrogen in Tregs, B cells, macrophages, and DCs (43). However, whether androgen can modulate PD-1 expression is largely unknown. As a result, the mechanism by which androgen suppresses PD-1 expression is completely unknown. One of our findings, which we believe to be novel, is a mechanism by which androgen suppresses PD-1 expression in the spleen. Specifically, we demonstrated that the AR bound to the PD-1 promoter via an ARE, suppressing its transcription and mRNA and protein expression in the spleen (Figure 9 and Supplemental Figure 23).

Given that androgen exerts a pleiotropic effect on various organs and systems through both genomic and nongenomic mechanisms (37), it is conceivable that androgen aggravates Aldo-salt–induced AAs through multiple mechanisms. Consistent with this notion, it has been shown that androgen exacerbates Ang II–induced AAA through the Ang II–type-1A receptor IL-1α and TGF-β1 (6, 16). The current study identified 65 signaling pathways that were downregulated and 19 signaling pathways that were upregulated by androgen in the aorta following Aldo-salt administration (Figure 6 and Supplemental Tables 1−4). While the role of these signaling pathways in Aldo-salt–induced AAs remains to be investigated, we demonstrate that IL-6, a pleiotropic cytokine, along with PD-1, was implicated in Aldo-salt–induced AAs (Figure 5 and Supplemental Figures 9 and 10). It has been shown that IL-6 augments TCR–induced PD-1 expression via STAT3 and STAT4 in splenic T cells (44). However, it is unlikely that PD-1 regulates IL-6 expression in splenic T cells, as genetic deletion of PD-1 did not affect IL-6 expression in the spleen or serum of mice subjected to HFD feeding and Ang II infusion (Supplemental Figure 27). Thus, further investigation is warranted to define how PD-1 and IL-6 coordinate to mediate Aldo-salt–induced and androgen-mediated AAs.

The current studies have several limitations. First, most experiments were conducted in mice given Aldo-salt and lacked a control group without Aldo-salt administration. Consequently, how the Aldo/MR signaling coordinates with androgen/AR signaling in the regulation of PD-1 expression in T cells and AAs remains unclear. Second, the current studies exclusively focused on the TCR and PD-1 signaling pathways resulting from 180 androgen response genes in the aorta, neglecting exploration of the potential role of metabolic pathways enriched from 150 androgen response genes in Aldo-salt–induced AAs. Third, LMT-28 may have potential off targets relative to IL-6 inhibition. Addressing these limitations in future studies will contribute to a more comprehensive understanding of the mechanisms by which androgen aggravates AAs.

Given that immune checkpoint inhibitors (i.e., anti–PD-1 Abs) have been successful in cancer treatment and have revolutionized the cancer research field (11), an increasing number of patients with cancer have undergone immune checkpoint therapy (27). However, immune checkpoint therapy is associated with serious immune-related cardiovascular adverse events, including autoimmune myocarditis, pericarditis, and vasculitis (27). In alignment with these findings, the current studies suggest an increased risk of developing AAs for patients undergoing immune checkpoint inhibitor therapy. Indeed, a recent case report showed that a 57-year-old man with lung adenocarcinoma treated with chemotherapy and immune checkpoint blockade developed an inflammatory TAA (45). Thus, patients with cancer who are predisposed to the risk factors for AAs, such as men, those of older age, and those who smoke, may have an increased likelihood of developing AAs during immune checkpoint therapy. As a precaution, these patients should be advised to undergo ultrasound screening for AAs to increase the life-saving potential of cancer immunotherapy.

## Methods

Details on the methods and materials used are provided in the supplemental material.

### Sex as a biological variable.

Our study examined male and female animals, and sex-dimorphic effects are reported. Human aortic samples were obtained from men.

### Statistics.

All data are expressed as the mean ± SEM. To compare 1 parameter between 2 groups, normality tests were conducted. If data passed the normality test, a parametric, unpaired, 2-tailed *t* test was used. If data did not pass the normality test, a nonparametric, unpaired, 2-tailed *t* test was used. For multiple comparisons of 2 parameters among multiple groups, a 2-way ANOVA was performed with correction for multiple comparisons by controlling the FDR. Similarly, for multiple comparisons of 3 parameters among multiple groups, a 3-way ANOVA was used with correction for multiple comparisons by controlling the FDR. The incidence of AAs between the 2 groups was compared using a 2-sided χ^2^ test. The relationship between 2 quantitative variables was analyzed through simple linear regression. Significant outliers, identified by the outlier calculator (GraphPad), were excluded from the statistical analysis. All statistical analyses were carried out using GraphPad Prism 9 software (GraphPad Software). A *P* value or adjusted *P* value of less than 0.05 was considered significant unless otherwise specified A *P* value of greater than 0.05 was considered not significant.

### Study approval.

All animal procedures were approved by the IACUC of the University of Kentucky. All procedures for the use of human AA specimens in the current study were approved by the IRB of the University of Kentucky.

### Data availability.

RNA-Seq data were deposited in the NCBI’s Gene Expression Omnibus (GEO) database (GEO GSE255682). Values for all data points in graphs are reported in the Supporting Data Values file. Requests for materials should be directed to the corresponding authors and will be fulfilled upon completion of appropriate material transfer agreements.

## Author contributions

XM, SL, ZW, and KJ contributed to designing research studies, conducting experiments, acquiring data, and analyzing data.; TM, AJS, and AVT contributed to analyzing RNA-Seq data. ESL and JAC contributed to providing human AA specimens. MCG and ZG contributed to the conceptualization, supervision, writing, project administration, and funding acquisition.

## Supplementary Material

Supplemental data

Unedited blot and gel images

Supporting data values

## Version 1 06/20/2024

In-Press Preview

## Version 2 08/01/2024

Electronic publication

## References

[1] Isselbacher EM (2005). Thoracic and abdominal aortic aneurysms. Circulation.

[2] Kent KC (2014). Clinical practice. Abdominal aortic aneurysms. N Engl J Med.

[3] Golledge J (2006). Abdominal aortic aneurysm: pathogenesis and implications for management. Arterioscler Thromb Vasc Biol.

[4] Robinet P (2018). Consideration of sex differences in design and reporting of experimental arterial pathology studies-statement from ATVB Council. Arterioscler Thromb Vasc Biol.

[5] Henriques TA (2004). Orchidectomy, but not ovariectomy, regulates angiotensin II–induced vascular diseases in apolipoprotein E-deficient mice. Endocrinology.

[6] Henriques T (2008). Androgen increases AT1a receptor expression in abdominal aortas to promote angiotensin II–induced AAAs in apolipoprotein E-deficient mice. Arterioscler Thromb Vasc Biol.

[7] Cho BS (2009). Differential regulation of aortic growth in male and female rodents is associated with AAA development. J Surg Res.

[8] McCurley A, Jaffe IZ (2012). Mineralocorticoid receptors in vascular function and disease. Mol Cell Endocrinol.

[9] Liu S (2013). Mineralocorticoid receptor agonists induce mouse aortic aneurysm formation and rupture in the presence of high salt. Arterioscler Thromb Vasc Biol.

[10] Lutshumba J (2018). Deletion of BMAL1 in smooth muscle cells protects mice from abdominal aortic aneurysms. Arterioscler Thromb Vasc Biol.

[11] Sharma P, Allison JP (2015). Immune checkpoint targeting in cancer therapy: toward combination strategies with curative potential. Cell.

[12] Daugherty A (2001). Antagonism of AT2 receptors augments angiotensin II–induced abdominal aortic aneurysms and atherosclerosis. Br J Pharmacol.

[13] Chaikof EL (2018). The Society for Vascular Surgery practice guidelines on the care of patients with an abdominal aortic aneurysm. J Vasc Surg.

[14] Lee DL (2006). Angiotensin II hypertension is attenuated in interleukin-6 knockout mice. Am J Physiol Heart Circ Physiol.

[15] Gubbels Bupp MR, Jorgensen TN (2018). Androgen–induced immunosuppression. Front Immunol.

[16] Huang CK (2015). Androgen receptor promotes abdominal aortic aneurysm development via modulating inflammatory interleukin-1α and transforming growth factor-β1 expression. Hypertension.

[17] Yang Z (2007). ASC-J9 ameliorates spinal and bulbar muscular atrophy phenotype via degradation of androgen receptor. Nat Med.

[18] Lin TH (2013). Anti-androgen receptor ASC-J9 versus anti-androgens MDV3100 (Enzalutamide) or Casodex (Bicalutamide) leads to opposite effects on prostate cancer metastasis via differential modulation of macrophage infiltration and STAT3-CCL2 signaling. Cell Death Dis.

[19] Davis JP (2016). Pharmacologic blockade and genetic deletion of androgen receptor attenuates aortic aneurysm formation. J Vasc Surg.

[20] Abbass A (2017). Liddle syndrome in association with aortic dissection. Cureus.

[21] Lindsay ME, Dietz HC (2011). Lessons on the pathogenesis of aneurysm from heritable conditions. Nature.

[22] Harrison SC (2013). Interleukin-6 receptor pathways in abdominal aortic aneurysm. Eur Heart J.

[23] Hong SS (2015). A novel small-molecule inhibitor targeting the IL-6 receptor β subunit, glycoprotein 130. J Immunol.

[24] Naamneh Elzenaty R (2022). Basics of androgen synthesis and action. Best Pract Res Clin Endocrinol Metab.

[25] Love MI (2014). Moderated estimation of fold change and dispersion for RNA-Seq data with DESeq2. Genome Biol.

[26] Chen EY (2013). Enrichr: interactive and collaborative HTML5 gene list enrichment analysis tool. BMC Bioinformatics.

[27] Zhang L (2021). The evolving immunotherapy landscape and the epidemiology, diagnosis, and management of cardiotoxicity: *JACC: CardioOncology* Primer. JACC CardioOncol.

[28] Weyand CM (2018). The immunoinhibitory PD-1/PD-L1 pathway in inflammatory blood vessel disease. J Leukoc Biol.

[29] Conforti F (2021). Sex-based differences in response to anti–PD-1 or PD-L1 treatment in patients with non-small-cell lung cancer expressing high PD-L1 levels. A systematic review and meta-analysis of randomized clinical trials. ESMO Open.

[30] Perrotta M (2018). Deoxycorticosterone acetate-salt hypertension activates placental growth factor in the spleen to couple sympathetic drive and immune system activation. Cardiovasc Res.

[31] Lewis SM (2019). Structure and function of the immune system in the spleen. Sci Immunol.

[32] Massie CE (2007). New androgen receptor genomic targets show an interaction with the ETS1 transcription factor. EMBO Rep.

[33] Chen S (2012). Androgen receptor serine 81 phosphorylation mediates chromatin binding and transcriptional activation. J Biol Chem.

[34] Keir ME (2007). PD-1 regulates self-reactive CD8^+^ T cell responses to antigen in lymph nodes and tissues. J Immunol.

[35] Police SB (2009). Obesity promotes inflammation in periaortic adipose tissue and angiotensin II–induced abdominal aortic aneurysm formation. Arterioscler Thromb Vasc Biol.

[36] Koga N (2004). Blockade of the interaction between PD-1 and PD-L1 accelerates graft arterial disease in cardiac allografts. Arterioscler Thromb Vasc Biol.

[37] Lucas-Herald AK (2017). Genomic and non-genomic effects of androgens in the cardiovascular system: clinical implications. Clin Sci (Lond).

[38] Son BK (2019). Testosterone inhibits aneurysm formation and vascular inflammation in male mice. J Endocrinol.

[39] Xiong W (2004). Key roles of CD4^+^ T cells and IFN-γ in the development of abdominal aortic aneurysms in a murine model. J Immunol.

[40] Sharma AK (2012). Experimental abdominal aortic aneurysm formation is mediated by IL-17 and attenuated by mesenchymal stem cell treatment. Circulation.

[41] Ait-Oufella H (2013). Natural regulatory T cells limit angiotensin II–induced aneurysm formation and rupture in mice. Arterioscler Thromb Vasc Biol.

[42] Sun P (2021). Immune checkpoint programmed death-1 mediates abdominal aortic aneurysm and pseudoaneurysm progression. Biomed Pharmacother.

[43] Dinesh RK (2010). PD-1, gender, and autoimmunity. Autoimmun Rev.

[44] Austin JW (2014). STAT3, STAT4, NFATc1, and CTCF regulate PD-1 through multiple novel regulatory regions in murine T cells. J Immunol.

[45] Ninomiya R (2022). Inflammatory thoracic aortic aneurysm in a patient with advanced lung adenocarcinoma treated with pembrolizumab. Intern Med.

