# Supplementary material for: Androgen aggravates aortic aneurysms via suppression of PD-1 in mice
Source: J Clin Invest. 2024 Jun 20;134(15):e169085. doi: 10.1172/JCI169085 (PMC11290977; doi:10.1172/JCI169085)
Supplement: Supplemental data [file jci-134-169085-s205.pdf]

Supplementary Material

Androgen aggravates aortic aneurysms via suppressing PD-1 in mice

Xufang Mu[1,], Shu Liu[2,], Zhuoran Wang[1], Kai Jiang[2], Tim McClintock[2], Arnold J. Stromberg[3, †], Alejandro V.Tezanos[3], Eugene S Lee[4], John A. Curci[5], Ming C Gong[2,6,*], and Zhenheng Guo[1,6,7,*]

[1]Departments of Pharmacology and Nutritional Sciences, [2]Physiology, [3]Statistics, and [6]Saha Cardiovascular Research Center, University of Kentucky, Lexington, KY; [4]Department of Research, Sacramento VA Medical Center, Mather, CA; [5]Department of Vascular Surgery, Vanderbilt University, Nashville, TN; [7]Department of Research, Lexington Veterans Affairs Medical Center, Lexington, KY, USA

The authors have declared that no conflict of interest exists

[†]Deceased September 17, 2023

[*]To whom correspondence should be addressed:

Ming C. Gong, Ph.D.
Email: ming.gong@uky.edu

Zhenheng Guo, Ph.D.
email: zguo2@uky.edu

This PDF file includes

Supplementary text (Material and Methods)

Supplemental Figures 1 to 30

Supplemental Tables 1 to 8

Supplemental References

Material and Methods

Animals

C57BL/6J male and female mice aged 9-10 months were purchased from the National Institute on Aging (Charles River) or generated by in-house breeding. Global PD-1 knockout mice (strain #: 028276) and WT C57BL/6J mice (Strain #:000664) were purchased from the Jackson Laboratory. PD-1 knockout mice have been bred with C57BL/6J mice for at least 15 generations. All mice were housed in the facilities of the Division of Laboratory Animal Resources at the University of Kentucky. Mice were fed a chow diet with free access to drinking water ad libitum. The sex and age of mice in the current study were explicitly specified in the result and figure legend.

Administration of mice with aldosterone and high salt (Aldo-salt) to induce aortic aneurysms

Aortic aneurysms were induced by subcutaneous implantation of osmotic minipumps (Alzet model 2004; 28-day release; DURECT) containing aldosterone (Aldo; 200 μg/kg/day in 50% DMSO) into C57BL/6J mice with free access to a high salt drinking water (0.9% NaCl and 0.2% KCl) for four or eight weeks as described (1-3).

In vivo aortic ultrasound imaging

The maximal intraluminal diameters of suprarenal abdominal aortas and aortic arches were measured by a high-resolution ultrasound imaging system (Vevo 2100 and Vevo 3100, VisualSonics) in mice one week before (to set up a baseline) and weekly four or eight weeks after Aldo-salt or high-fat diet (HFD) and angiotensin II (Ang II) administration. The procedures for measuring the maximal intraluminal diameters of the suprarenal aortas by ultrasound were described previously (1-3). The procedures for measuring the maximal intraluminal diameters of the aortic root and arch by ultrasound were adapted from Sawada et al. (4). The growth rate of the suprarenal aortic diameters in response to Aldo-salt was calculated from the ultrasound data: growth rate (mm/week) = (the internal

diameters of the suprarenal aorta four weeks after Aldo-salt administration - baseline of the suprarenal aorta) / 4 week.

Blood pressure measurement

Mouse blood pressures were measured by a non-invasive tail-cuff method (Coda 8; Kent Scientific) one week before (to set up a baseline) and three or seven weeks after Aldo-salt or HFD and Ang II administration, as described previously (1-3).

***Ex vivo* morphometric analysis of the aorta**

The aortas were isolated from mice four or eight weeks after Aldo-salt or HFD and Ang II administration. After dissection to remove fat tissues, the aortas were photographed by the Nikon SMZ2800 stereo microscope with a digital camera. The maximal diameters of the ascending aortas, aortic arch, descending aortas, and suprarenal abdominal aortas were analyzed by Nikon NIS-Element software as described previously (1-3).

Definition, classification, and quantification of aortic aneurysms

Aldo-salt- or HFD and Ang II-induced AAA and TAA were defined as having at least a 50% increase in the maximal internal (intraluminal) or external diameters in mice administered Aldo-salt compared with the same region of the aorta in control mice or the appearance of evident aortopathy as described previously (1-3). Aldo-salt-induced aortic aneurysms were classified as Type I (the gross appearance of aortic dilation), Type II (at least a 2-time increase in external diameter of the aorta), Type III (a pronounced bulbous form of Type II with a thrombus), and Type IV (aortic rupture) as adapted from Daugherty et al. (5) and described in Supplemental Figure 3. Aldo-salt- or HFD and Ang II-induced aortic aneurysms were quantified as the percentage of the incidence of total aortic aneurysms, including abdominal aortic aneurysms (AAA), thoracic aortic aneurysms (TAA), and aortic aneurysm rupture, as described previously (1-3).

Orchiectomy

The procedures for orchiectomy and sham operation were adapted from Henriques et al. (6). Briefly, orchiectomy was performed on mice two weeks before Aldo-salt administration. After anesthetization with 2-3% isoflurane, a single midline incision was made at the caudal abdominal aorta. The skin was separated from the muscle layer, and a similar longitudinal incision was made through the muscle. Both testes can be reached through the same incision. A knot was tied under the testes to reduce bleeding. After the testes and epididymis were excised with scissors, the cut end was cauterized and put back under the muscle. The incision was closed in two layers with a suture. Sham operations were subject to a similar surgical procedure without removing testes.

Blood testosterone measurement

Blood was collected by cardiac puncture from orchiectomized and sham-operated mice administered Aldo-salt for four weeks or male mice with and without 10-day Aldo-salt administration. The collected blood was processed into serum or plasma as described previously (1-3). Serum or plasma testosterone levels were measured by a testosterone ELISA kit (Enzo Life Sciences) according to the manufacturer's instructions.

Urinary and serum sodium measurement and salt retention analysis

Mice were individually housed in metabolic cages (Tecniplast) to measure 24-h food and water intake and collect 24-h urine samples one week before (to set up a baseline) and three weeks after Aldo-salt administration. Serum was collected four weeks after Aldo-salt administration. The urinary and serum sodium concentrations [Na] were measured by a dual-channel flame photometer (Cole-Parmer) according to the manufacturer's instructions. The 24-h sodium retention was calculated as described by Lee et al. (7) as 24-h sodium intake – 24-h urinary sodium excretion, where 24-h sodium intake =

(24 h food weight x food [Na]) + (24 h drinking water x drinking water [Na]), and 24-h urinary sodium excretion = 24 h urine volume x urine [Na].

Dihydrotestosterone (DHT) pellet administration to orchiectomized mice

DHT pellets (10 mg, 60-day release) were purchased from Innovative Research of America. Mice were orchiectomized for two weeks (to deprive gonadal androgen) and then administered with Aldo-salt (to induce aortic aneurysms) with or without subcutaneous DHT pellet implantation (to restore androgen) for four weeks. The procedures for administering DHT pellets to orchiectomized mice were adapted from Henriques et al. (8).

Treatment of mice with ASC-J9

ASC-J9, an AR degradation enhancer (9), was purchased from Advanced ChemBlocks. ASC-J9 was dissolved in DMSO (25 mg/120 μl DMSO) and mixed completely until it had a clear yellow color. The mixed solution was then diluted in a pre-warmed (55°C) sesame oil (Millipore Sigma) to 1,200 μl, mixed completely, and then immediately injected into mice (50 mg ASC-J9/kg mouse BW, i.p.; to induce AR degradation), and once a day with Aldo-salt (to induce aortic aneurysms) for four weeks. The procedures for administering ASC-J9 to mice were adapted from Huang et al. (10).

Immunocytochemistry (IHC)

The aorta, spleen, and lymph nodes were isolated from mice ten days, four weeks, and eight weeks after Aldo-salt or HFD and Ang II administration, as indicated in the manuscript and figure legend, respectively. The human aortas with and without aortic aneurysms were obtained from Drs. Eugene Lee (Sacramento Veterans Affairs Medical Center) and John Curci (Vanderbilt University). All tissues were embedded in paraffin and then cut into 5-μm thickness sections.

Aortic elastin breaks were detected by Verhoeff-Van Gieson staining in longitudinal sections of the

thoracic aortas and cross-sections of the abdominal aorta with an Elastic Stain Kit (Fisher Scientific) according to the manufacturer's instructions. Aortic elastin breaks were quantified by the manual count of elastin breaks under a microscope and expressed as total elastin breaks/section/mice as described previously(2, 3, 11).

The aorta, spleen, and lymph node sections were incubated with various primary antibodies (Ab) for immunostaining detection of various protein expressions. The information on the primary antibodies used in the immunohistological staining was described in Supplemental Table 7. Pilot studies with different Ab dilutions were conducted for all primary Ab to obtain their optimal concentrations for immunostaining. The specificity of all antibodies was verified using tissue sections from knockout mice, if available, or a nonspecific IgG (Gong and Guo, unpublished observations). The procedure for histological and immunohistological staining was described previously (2, 3, 11). Immunostaining was quantified by ImageJ (https://imagej.nih.gov/ij/) and expressed as an average percentage of area fractions from three to five random images per section per mouse as described (12).

Treatment of mice with flutamide

Flutamide, a selective antagonist of AR (13), was purchased from Millipore Sigma. Flutamide was dissolved in ethanol and then diluted with corn oil (Millipore Sigma) to 10% (v/v). Mice were administered Aldo-salt (to induce aortic aneurysms) with flutamide (50 mg/kg; to inhibit AR) or vehicle (10% ethanol in corn oil) via intraperitoneal injection once a day for four weeks. The procedures for i.p. flutamide injection to mice were adapted from Davis et al. (13).

Quantitative analysis of mRNA expression

The procedures for RNA purification, reverse transcription, and real-time PCR were described previously (14-19). The sequence of the real-time PCR primers was described in Supplemental Table 8. All PCR primers were designed to cross introns of chromosomes to eliminate potential genomic

DNA contamination and verified by dissociation curve analysis to ensure the specificity of the PCR primers. All mRNA expressions were normalized to the housekeeping gene 36B4 (also known as Rplp0) and quantified by a delta-delta Ct method as described previously (14-19).

RNA sequence (RNA-seq) and bioinformatics analysis

The whole aortas were isolated from three groups of mice (5 mice/group): 1) administered Aldo-salt for one week; 2) orchiectomy followed by one-week Aldo-salt administration; 3) orchiectomy followed by one-week Aldo-salt with DHT pellet administration. After storage and dissection in RNAlater solution (FisherScientific), total RNA was purified using an RNeasy mini-kit (Qiagen) and then sent to the Genomics Core Laboratory at the University of Kentucky to measure the integrity and purity of purified RNA by Agilent Bioanalyzer 2100 using an RNA nano Chip kit (Agilent Technologies). Purified total RNA samples with RNA integrity number (RIN) > 9 were sent to Novogene for Illumina RNA-seq with a sequencing depth of 20 million reads per sample.

RNA-Seq data in the form of compressed Fastq files from Novogene were uploaded to the Illumina BaseSpace Sequencing Hub (Illumina) and were aligned to the mouse genome (build mm10) using the STAR Aligner v2.5.2a. After alignment, differentially abundant mRNAs were identified using DESeq2 (20). The mRNAs with $p < 0.01$ were considered differentially abundant. Log2 (fold change) and -log10 (p-value) were used for the visualization of differences in transcript abundance in the form of volcano plots using Prism 9 (GraphPad). Venn diagrams and heatmaps were generated by TBtools, a Toolkit for Biologists (21). The 180 mRNAs upregulated by orchiectomy but downregulated by DHT and 150 mRNAs downregulated by orchiectomy but upregulated by DHT were subjected to comprehensive pathway enrichment analyses via Enrichr using the Bioplanet 2019 database (https://maayanlab.cloud/Enrichr/) (22). The pathways with adjusted $p < 0.01$ (Benjamini-Hochberg method) were considered significant.

Flow cytometry analysis of immune cells

The procedures for flow cytometry analysis of immune cells in the mouse aorta, spleen, lymph node, and blood were adapted from Galkina et al. (23) and Melak et al. (24). The whole aorta was isolated from mice perfused with PBS containing 2% heparin. Isolated aortas were dissected to remove fatty tissue while keeping the adventitia intact. The aortas were cut into small pieces and then incubated with 450 U/mL collagenase type I, 125 U/mL collagenase type XI, 60 U/mL hyaluronidase type I-s, and 60 U/mL DNase-I in DMEM media at 37 ^{0}C for 1 hour with gentle shaking and vortex or for 15 minutes followed by the dissociation with gentleMACS Dissociator (Miltenyi Biotec) using the presetting program (heart program) with repeating the procedure three times to process the aorta into single cell suspension. All enzymes except collagenase type I (Worthington Biochemicals) were obtained from Millipore Sigma.

The spleen, peri-aortic lymph node, and blood were harvested from mice perfused with PBS containing 2% heparin. A small piece of the spleen was cut to prepare splenic cell suspension. The small pieces of the spleen and the whole spleen were weighed to calculate the total immune cells in the spleen. The bilateral lymph nodes at the bifurcation of the aorta were collected for flow cytometry. A single cell suspension was obtained by using syringe plunges to mash the small piece of the spleen, digested aortas, and pooled lymph nodes through a 70-μm strainer (Fisher Scientific). Blood and splenic cells were incubated with an RBC lysis buffer (eBioscience) to remove erythrocytes.

Single-cell suspensions were first incubated with an anti-mouse CD16/32 Ab (Biolegend) to block the nonspecific binding of the immunoglobulin to Fc receptors and then stained with various primary Ab conjugated with different fluorescence (Supplemental Table 7) for thirty minutes in the dark. After washing, cells were fixed by a stabilizing fixative solution (BD Biosciences) and mixed with Precision Count Beads (Biolegend) to obtain absolute counts of cells by flow cytometry. Immunofluorescence was detected by flow cytometry (BD Symphony 2.0 and Full spectral Aurora Cytek) in the University

of Kentucky Flow Cytometry and Immune Monitoring Core Facility. Cells from the spleen, lymph nodes, and blood were also stained with all the fluorophores minus one of them (FMO) to identify gating boundaries and ensure the specificity of antibodies. Data were analyzed using FlowJo™ v10.

Western blot

The procedures for using the trichloroacetic acid (TCA) method to prepare protein samples from the spleen and peri-aortic lymph nodes for Western blot analysis were described previously (16-18). The information on anti-mouse PD-1, CD3ε, CD19, and GAPDH were described in Supplemental Table 7. Western blots were quantified by ImageJ as described (16-18).

AR ChIP assay

A splenic cell suspension was prepared by mashing one freshly isolated mouse spleen through a 70-μm strainer for the AR ChIP assay. Approximately 4 x 10^6 splenic cells were used for one ChIP assay. SimpleChIP® Enzymatic Chromatin IP Kit (Cell Signaling) was used for the AR ChIP assay. Two ChIP-grade anti-mouse AR antibodies with different epitopes (Supplemental Table 7) were used in the AR ChIP assay to pull down chromatins containing the PD-1 promoter. A normal rabbit IgG (Cell Signaling) was included in the AR ChIP assay as a negative control to ensure the specificity of AR immunoprecipitation. Two sets of ChIP-PCR primers were designed to amplify androgen-response element (ARE)4-5 and ARE 6 in the mouse PD-1 promoter (Figure 9J), respectively. The sequences of ChIP PCR primers were described in Supplemental Figure 8. The binding of AR to the mouse PD-1 promoter was quantified by ImageJ and expressed as the percentage of genomic DNA input as described (18, 19).

AR promoter assay

A 488-bp mouse PD-1 promoter (-4,444 to -3,956 bp relative to TSS) containing putative ARE6-10 (Figure 7J) was synthesized by Integrated DNA Technology (IDT) and then subcloned into the pGL3-

basic firefly luciferase reporter vectors (Promega) in Kpn I and Hind III restriction enzyme sites. The pGL3-basic-PD-1 promoter construct (0.75 μg) was co-transfected with a pcDNA Flag-M4-human AR construct (3 μg), provided by Dr. Steven Balk (Addgene plasmid # 171240) (25), with 1:4 molecular ratio of PD-1 promoter to AR, into AD-HEK 293 cells (Stratagene) by LipoFexin (Lamda Biotech) according to the manufacturer's instructions. A pcDNA3.1 vector (Invitrogen; 2.25 μg) was added to the cells with the pGL3-basic vector or the PD-1 promoter only to maintain the same ratio of DNA to LipoFexin. A Renilla luciferase control reporter vector (pRL-TK control vector; 10 ng; Promega) was included in all samples to control transfection efficiency. After 4-hour transfection, the cell culture medium was changed to a DMEM supplemented with 10% charcoal dextran-stripped fetal bovine serum (VWR) to minimize potential endogenous hormone effects. DHT (100 nM) was added into the cells with the pcDNA Flag-M4-AR construct to activate AR after 24-h transfection. Cells were harvested after 48-h transfection. The PD-1 promoter activity was analyzed by a modified dual luciferase enzyme assay as described previously (17-19).

Treatment of orchiectomized mice with anti-PD-1 antibody

The rat monoclonal anti-mouse PD-1 antibody (clone 29F.1A12) and rat IgG2a isotype control antibody (clone 1-1) were purchased from Leinco Technologies. Mice were subjected to orchiectomy for two weeks and then administered Aldo-salt with the anti-PD1 or control Ab (200 μg/mice; i.p.; twice a week) for eight weeks. The procedures for administering anti-mouse PD-1 antibodies to mice were adapted from Koga et al. (26).

Adoptive T cell transfer

PD-1 KO and WT T cells were isolated from the spleens of 4-month-old male PD-1 KO and WT C57BL6J donor mice. Following the manufacturer's protocol, the spleens were dissociated into single splenic cell suspensions using the gentleMACS™ Dissociator (Miltenyi Biotec). T cells were isolated by MicroBeads conjugated to monoclonal anti-mouse CD90.2 Ab (Miltenyi Biotec) according to the

manufacturer's protocol. The freshly isolated T cells were adoptively transferred into 9-10-month-old orchiectomized C57BL/6J recipient mice via retro-orbital sinus injection (10^7 T cells/100µl/mouse) two days prior to and eight and eighteen days after Aldo-salt administration, as previously described (27).

Administration of mice with HFD feeding and Ang II infusion to induce aortic aneurysms

Two-month-old male global PD-1 knockout and WT mice were fed a high-fat diet (D12492, 60% kcal from fat; Research Diets) for two months to induce obesity. After one month of HFD feeding, the mice were infused with Ang II (1000 ng/kg/min) by osmotic minipumps (Alzet 2004) via subcutaneous implantation for 28 days to induce aortic aneurysms as previously described (28). The information on PCR primers for genotyping PD-1 knockout and WT mice was described in Supplemental Table 8.

IL-6 measurement

Serum was collected by cardiac puncture, and splenic lysates were prepared using the TCA method from PD-1 KO and WT mice four weeks after HFD and Ang II administration. The serum and splenic IL-6 levels were measured by a mouse IL-6 ELISA kit (Biolegend) following the manufacturer's protocol.

Statistical analysis

All data were expressed as mean ± SEM. To compare one parameter between the two groups, normality tests were initially conducted. If the data passed the normality test, a parametric, unpaired, and two-tailed t-test was employed. If the data did not pass the normality test, a nonparametric, unpaired, and two-tailed test was used. For multiple comparisons of two parameters among multiple groups, a two-way ANOVA was performed with correction for multiple comparisons using controlling the false discovery rate. Similarly, for multiple comparisons of three parameters among multiple groups, a three-way ANOVA was used with correction for multiple comparisons by controlling the false discovery rate. The incidence of aortic aneurysms between the two groups was compared using

a two-sided Chi-Square test. The relationship between two quantitative variables was analyzed through simple linear regression. Significant outliers, identified by the outlier calculator (GraphPad), were excluded from the statistical analysis. All statistical analysis was carried out using Prism 9 software (GraphPad). A *P*-value or adjusted *P*-value < 0.05 was considered significant unless specified somewhere. A *P*-value of > 0.05 was considered not significant (ns).

Study approval

All procedures to use mice for the current study were approved by the Institutional Animal Care and Use Committee of the University of Kentucky. All procedures to use human aortic aneurysm specimens for the current study were approved by the Institutional Review Board of the University of Kentucky.

References

1. Liu S, Gong MC, and Guo Z. A New Mouse Model for Introduction of Aortic Aneurysm by Implantation of Deoxycorticosterone Acetate Pellets or Aldosterone Infusion in the Presence of High Salt. *Methods Mol Biol.* 2017;1614:155-63.
2. Liu S, Xie Z, Daugherty A, Cassis LA, Pearson KJ, Gong MC, et al. Mineralocorticoid receptor agonists induce mouse aortic aneurysm formation and rupture in the presence of high salt. *Arterioscler Thromb Vasc Biol.* 2013;33(7):1568-79.
3. Lutshumba J, Liu S, Zhong Y, Hou T, Daugherty A, Lu H, et al. Deletion of BMAL1 in Smooth Muscle Cells Protects Mice From Abdominal Aortic Aneurysms. *Arterioscler Thromb Vasc Biol.* 2018;38(5):1063-75.
4. Sawada H, Chen JZ, Wright BC, Moorleghen JJ, Lu HS, and Daugherty A. Ultrasound Imaging of the Thoracic and Abdominal Aorta in Mice to Determine Aneurysm Dimensions. *J Vis Exp.* 2019(145).
5. Daugherty A, Manning MW, and Cassis LA. Antagonism of AT2 receptors augments angiotensin II-induced abdominal aortic aneurysms and atherosclerosis. *Br J Pharmacol.* 2001;134(4):865-70.
6. Henriques TA, Huang J, D'Souza SS, Daugherty A, and Cassis LA. Orchidectomy, but not ovariectomy, regulates angiotensin II-induced vascular diseases in apolipoprotein E-deficient mice. *Endocrinology.* 2004;145(8):3866-72.
7. Lee DL, Sturgis LC, Labazi H, Osborne JB, Jr., Fleming C, Pollock JS, et al. Angiotensin II hypertension is attenuated in interleukin-6 knockout mice. *Am J Physiol Heart Circ Physiol.* 2006;290(3):H935-40.
8. Henriques T, Zhang X, Yiannikouris FB, Daugherty A, and Cassis LA. Androgen increases AT1a receptor expression in abdominal aortas to promote angiotensin II-induced AAAs in apolipoprotein E-deficient mice. *Arterioscler Thromb Vasc Biol.* 2008;28(7):1251-6.

9. Yang Z, Chang YJ, Yu IC, Yeh S, Wu CC, Miyamoto H, et al. ASC-J9 ameliorates spinal and bulbar muscular atrophy phenotype via degradation of androgen receptor. *Nature medicine.* 2007;13(3):348-53.
10. Huang CK, Luo J, Lai KP, Wang R, Pang H, Chang E, et al. Androgen receptor promotes abdominal aortic aneurysm development via modulating inflammatory interleukin-1alpha and transforming growth factor-beta1 expression. *Hypertension.* 2015;66(4):881-91.
11. Liu S, Xie Z, Zhao Q, Pang H, Turk J, Calderon L, et al. Smooth muscle-specific expression of calcium-independent phospholipase A2beta (iPLA2beta) participates in the initiation and early progression of vascular inflammation and neointima formation. *J Biol Chem.* 2012;287(29):24739-53.
12. Crowe AR, and Yue W. Semi-quantitative Determination of Protein Expression using Immunohistochemistry Staining and Analysis: An Integrated Protocol. *Bio Protoc.* 2019;9(24).
13. Davis JP, Salmon M, Pope NH, Lu G, Su G, Meher A, et al. Pharmacologic blockade and genetic deletion of androgen receptor attenuates aortic aneurysm formation. *J Vasc Surg.* 2016;63(6):1602-12 e2.
14. Hou T, Su W, Duncan MJ, Olga VA, Guo Z, and Gong MC. Time-restricted feeding protects the blood pressure circadian rhythm in diabetic mice. *Proc Natl Acad Sci U S A.* 2021;118(25).
15. Su W, Xie Z, Guo Z, Duncan MJ, Lutshumba J, and Gong MC. Altered clock gene expression and vascular smooth muscle diurnal contractile variations in type 2 diabetic db/db mice. *Am J Physiol Heart Circ Physiol.* 2012;302(3):H621-33.
16. Xie Z, Gong MC, Su W, Turk J, and Guo Z. Group VIA phospholipase A2 (iPLA2beta) participates in angiotensin II-induced transcriptional up-regulation of regulator of g-protein signaling-2 in vascular smooth muscle cells. *J Biol Chem.* 2007;282(35):25278-89.
17. Xie Z, Liu D, Liu S, Calderon L, Zhao G, Turk J, et al. Identification of a cAMP-response element in the regulator of G-protein signaling-2 (RGS2) promoter as a key cis-regulatory

element for RGS2 transcriptional regulation by angiotensin II in cultured vascular smooth muscles. *J Biol Chem.* 2011;286(52):44646-58.

18. Xie Z, Su W, Liu S, Zhao G, Esser K, Schroder EA, et al. Smooth-muscle BMAL1 participates in blood pressure circadian rhythm regulation. *J Clin Invest.* 2015;125(1):324-36.

19. Zhao G, Zhong Y, Su W, Liu S, Song X, Hou T, et al. Transcriptional Suppression of CPI-17 Gene Expression in Vascular Smooth Muscle Cells by Tumor Necrosis Factor, Kruppel-Like Factor 4, and Sp1 Is Associated with Lipopolysaccharide-Induced Vascular Hypocontractility, Hypotension, and Mortality. *Molecular and cellular biology.* 2019;39(11).

20. Love MI, Huber W, and Anders S. Moderated estimation of fold change and dispersion for RNA-seq data with DESeq2. *Genome Biol.* 2014;15(12):550.

21. Chen C, Chen H, Zhang Y, Thomas HR, Frank MH, He Y, et al. TBtools: An Integrative Toolkit Developed for Interactive Analyses of Big Biological Data. *Mol Plant.* 2020;13(8):1194-202.

22. Chen EY, Tan CM, Kou Y, Duan Q, Wang Z, Meirelles GV, et al. Enrichr: interactive and collaborative HTML5 gene list enrichment analysis tool. *BMC Bioinformatics.* 2013;14:128.

23. Galkina E, Kadl A, Sanders J, Varughese D, Sarembock IJ, and Ley K. Lymphocyte recruitment into the aortic wall before and during development of atherosclerosis is partially L-selectin dependent. *J Exp Med.* 2006;203(5):1273-82.

24. Mellak S, Ait-Oufella H, Esposito B, Loyer X, Poirier M, Tedder TF, et al. Angiotensin II mobilizes spleen monocytes to promote the development of abdominal aortic aneurysm in Apoe-/- mice. *Arterioscler Thromb Vasc Biol.* 2015;35(2):378-88.

25. Chen S, Gulla S, Cai C, and Balk SP. Androgen receptor serine 81 phosphorylation mediates chromatin binding and transcriptional activation. *J Biol Chem.* 2012;287(11):8571-83.

26. Koga N, Suzuki J, Kosuge H, Haraguchi G, Onai Y, Futamatsu H, et al. Blockade of the interaction between PD-1 and PD-L1 accelerates graft arterial disease in cardiac allografts. *Arterioscler Thromb Vasc Biol.* 2004;24(11):2057-62.

27. Yardeni T, Eckhaus M, Morris HD, Huizing M, and Hoogstraten-Miller S. Retro-orbital injections in mice. *Lab Anim (NY).* 2011;40(5):155-60.

28. Police SB, Thatcher SE, Charnigo R, Daugherty A, and Cassis LA. Obesity promotes inflammation in periaortic adipose tissue and angiotensin II-induced abdominal aortic aneurysm formation. *Arterioscler Thromb Vasc Biol.* 2009;29(10):1458-64.

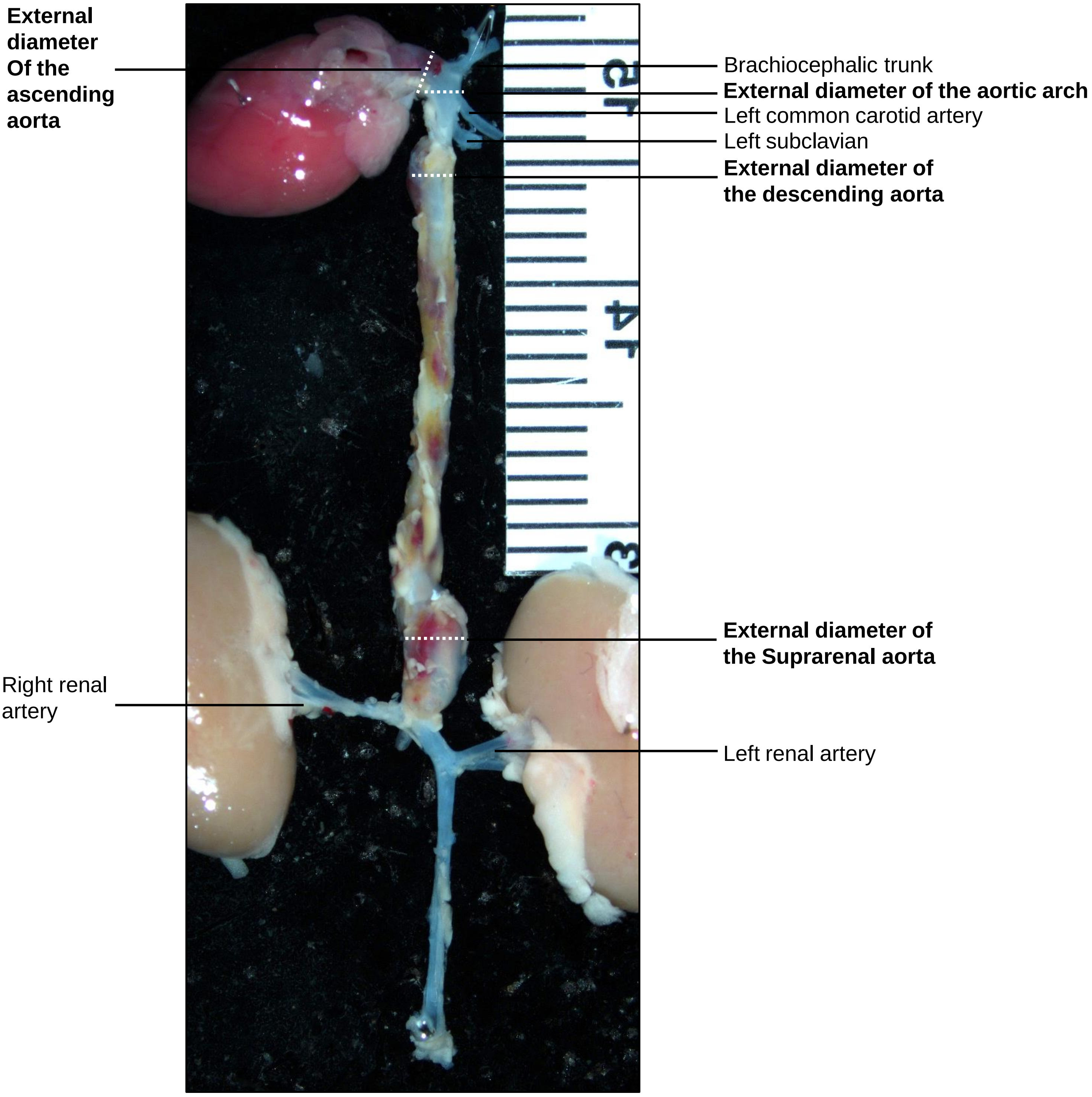

Supplemental Figure 1. ***Ex Vivo*** **measurement of maximal external diameters of the ascending aorta, aortic arch, descending aorta, and suprarenal aorta by microscopy.** The brachiocephalic trunk, left common carotid artery, left subclavian, right renal artery, and left renal artery are indicated. The dotted dash lines labeled in white show the maximal external diameters of the ascending aorta (AscAo), aortic arch (ArchAo), descending aorta (DesAo), and suprarenal aorta (SupAo), measured in the current study.

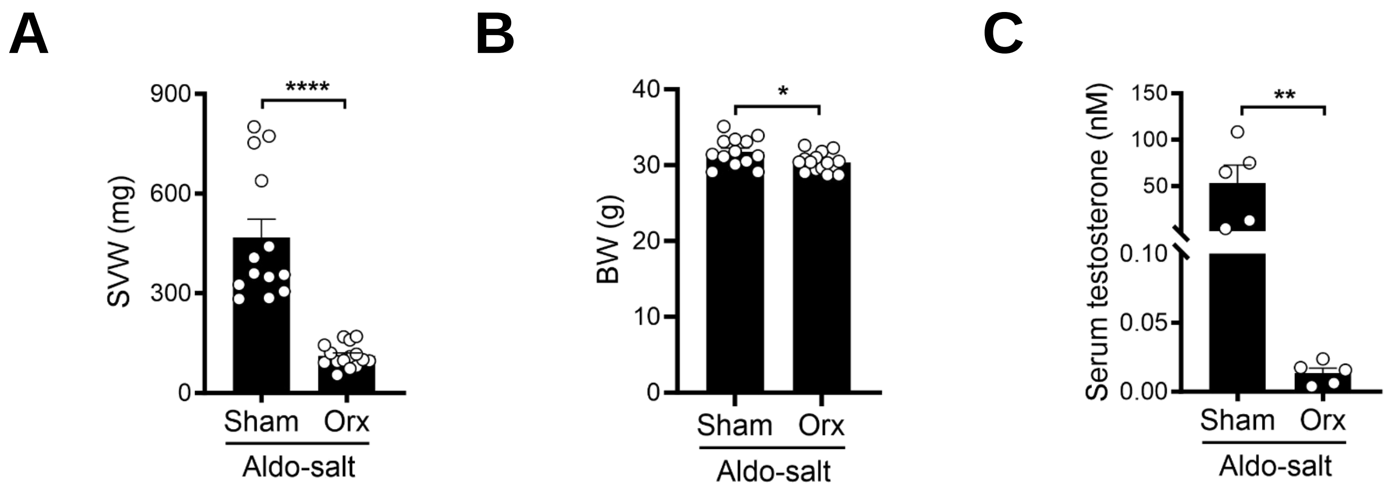

Supplemental Figure 2. Gonadal androgen deprivation decreases seminal vesicle weight, body weight, and serum testosterone level in mice administered Aldo-salt. The seminal vesicle weight (SVW; **A;** n = 13-15/group), body weight (BW; **B**; n = 13-15/group), and serum testosterone level (**C**; n = 5/group) were measured in 10-month-old male C57BL/6J mice with orchiectomy (orx) or sham operation four weeks after Aldo-salt administration. The data were expressed as mean ± SEM and analyzed by a two-tailed unpaired *t*-test. **, P < 0.05; **, P < 0.01; ****, P < 0.0001.*

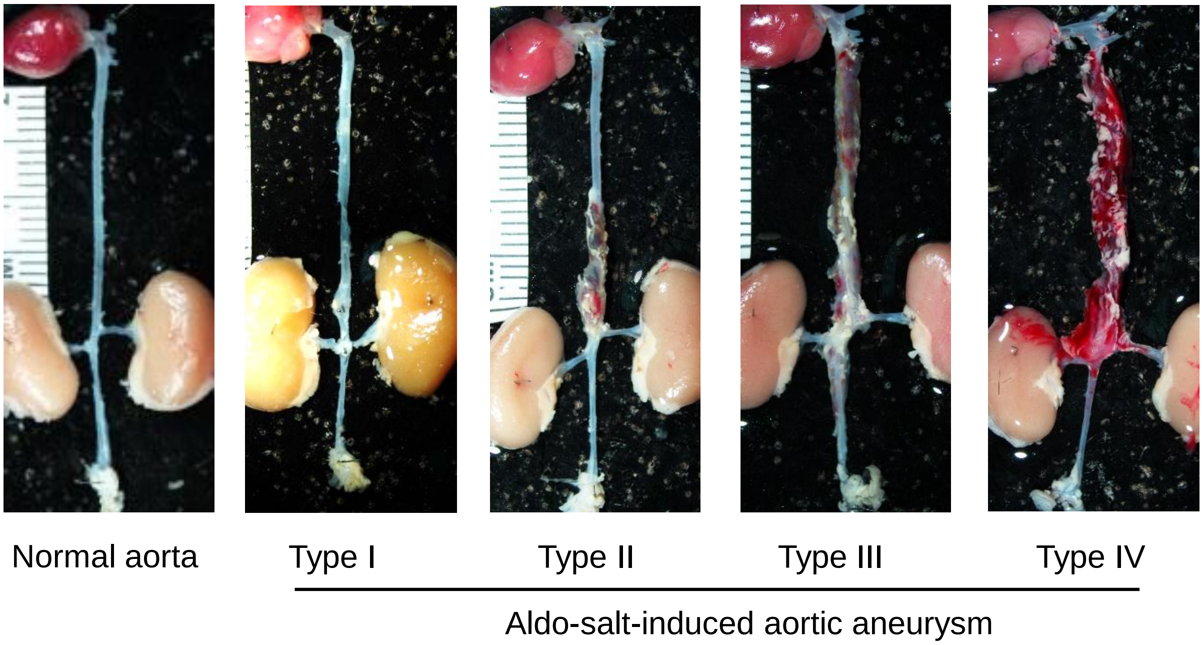

Supplemental Figure 3. Classification of Aldo-salt-induced aortic aneurysms in 10-month-old or older C57BL/6J mice. Aldo-salt-induced aortic aneurysms were classed as Type I (a gross appearance of the abdominal or thoracic aortic dilation compared with the normal aorta), Type II (at least two times the normal external diameter of the abdominal or thoracic aorta, frequently containing a thrombus), Type III (a pronounced bulbous form of type II that includes a thrombus in both the abdominal and thoracic aorta), and Type IV (aortic aneurysms with rupture).

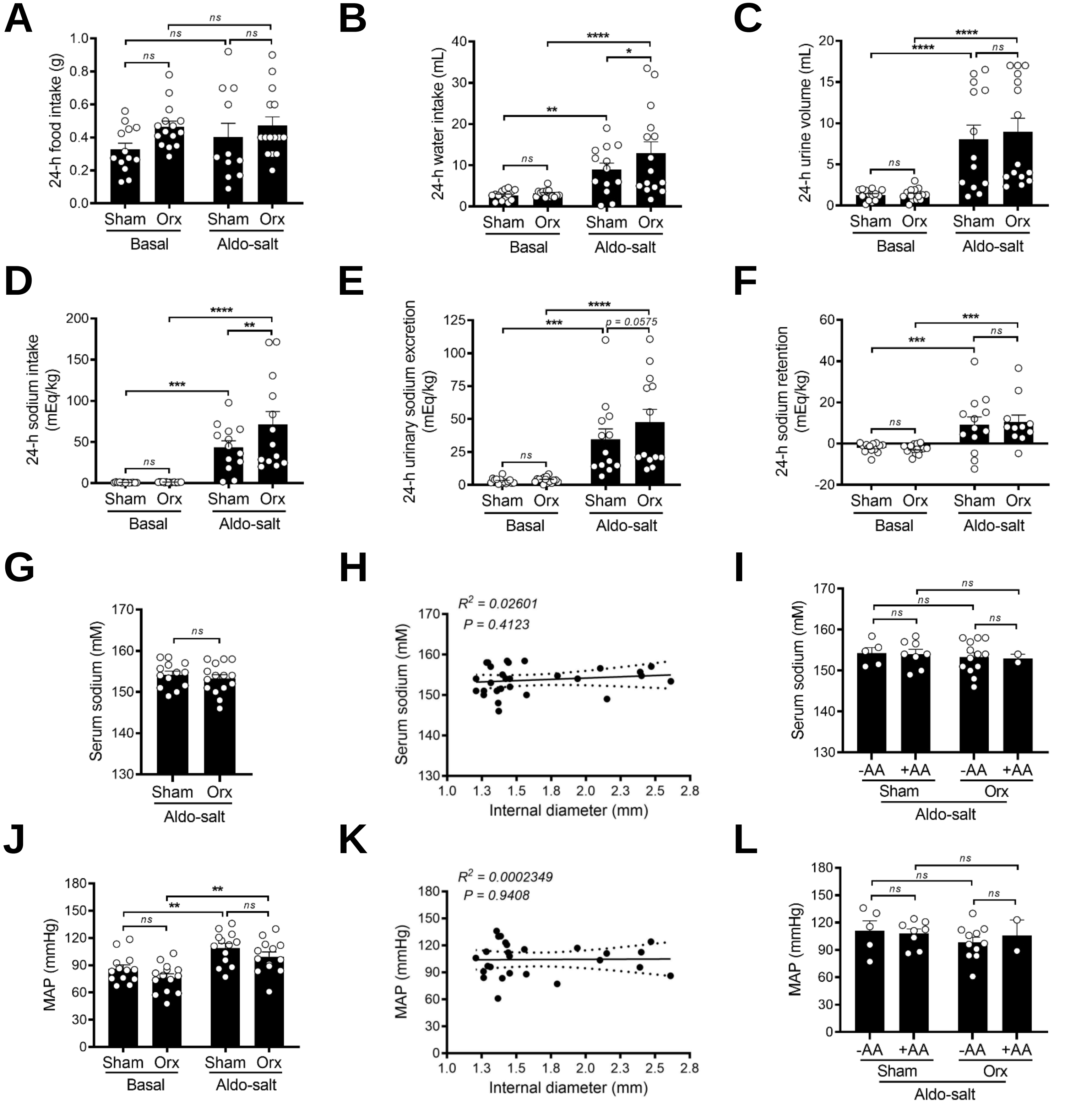

Supplemental Figure 4. Gonadal androgen deprivation does not affect Aldo-salt-induced sodium retention and hypertension. (**A**–**F**) 24-h food intake (A), water intake (B), urine volume (C), sodium intake (D), urinary sodium excretion (E), and sodium retention (F) were measured in 10-month-old male C57BL/6J mice with orx or sham operation one week before (basal) and three weeks after Aldo-salt administration (n = 13-15/group). (**G**) Serum sodium levels of the mice with orx and sham-operation four weeks after Aldo-salt administration (n = 13-15/group). (**H**) Correlation analysis of the internal diameter of the suprarenal aorta and serum sodium levels in the mice with orx and sham operation four weeks after Aldo-salt administration (n = 28/group). (**I**) Serum sodium levels of the orchiectomized and sham-operated mice with (+) and without (-) aortic aneurysms (AA) four weeks after Aldo-salt administration (n = 2-13/group). (**J**) Mean arterial pressure (MAP) of the orchiectomized and sham-operated mice one week before and three weeks after Aldo-salt administration (n = 13-15/group). (**K**) Correlation analysis of the internal diameter of the suprarenal aorta and MAP in mice with orx or sham operation three weeks after Aldo-salt administration (n = 28/group). (**L**) MAP of the orchiectomized and sham-operated mice with (+) and without (-) AA (n = 2-13/group). Data were expressed as mean ± SEM and analyzed by two-way ANOVA with multiple comparison tests (**A**–**F**, I, **J**, and **L**), two-tailed unpaired *t*-test (**G**), and simple linear regression analysis (**H** and **K**). **, P < 0.05; **, P < 0.01; ***, P < 0.001; ****, P < 0.0001; ns, not significant.*

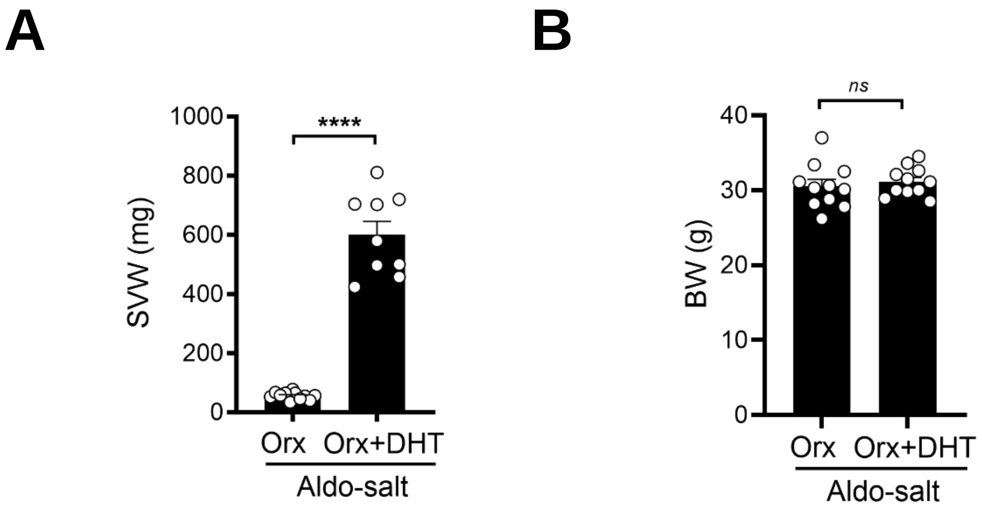

Supplemental Figure 5. Effects of exogenous dihydrotestosterone (DHT) on seminal vesicle and body weight in orchiectomized mice administered Aldo-salt. The SVW (**A**) and BW (**B**) were measured in 10-month-old male C57BL/6J mice with orchiectomy four weeks after Aldo-salt with and without dihydrotestosterone (DHT) pellet implantation (n = 9-11/group). The data were expressed as mean ± SEM and analyzed by a two-tailed unpaired *t*-test. ****, $P < 0.0001$.

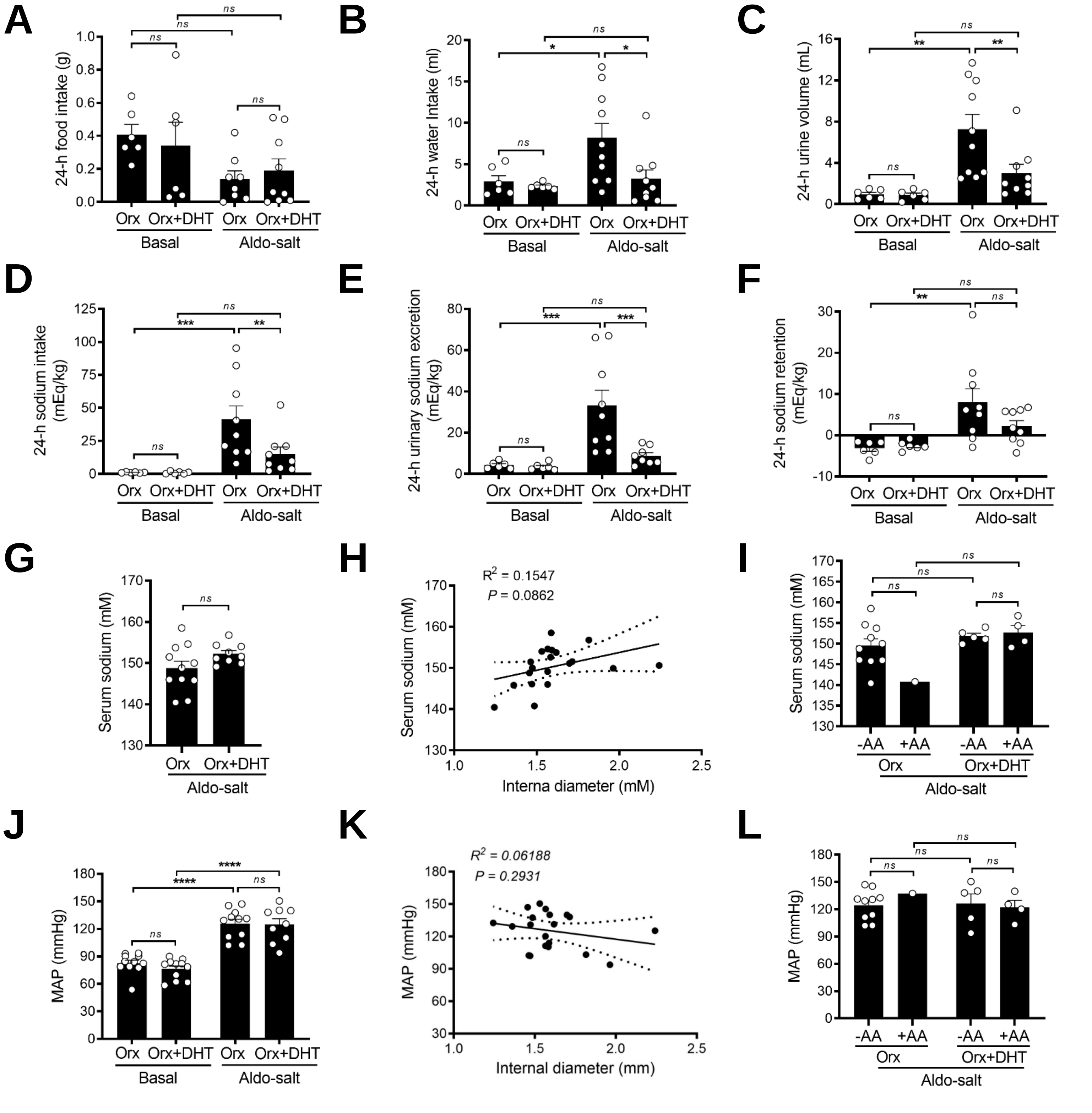

Supplemental Figure 6. Exogenous DHT administration to orchiectomized mice does not affect Aldo-salt-induced sodium retention and hypertension. (**A**–**F**) 24-h food intake (A), water intake (B), urine volume (C), sodium intake (D), urinary sodium excretion (E), and sodium retention (F) were determined in 10-month-old male C57BL/6J mice with orchiectomy one week before (basal) and three weeks after Aldo-salt with and without DHT pellet implantation (n = 6-9/group). (**G**) Serum sodium levels of the orchiectomized mice four weeks after Aldo-salt with or without DHT administration (n = 9-11/group). (**H**) Correlation analysis of the internal diameter of the suprarenal aorta and serum sodium levels in the orchiectomized mice four weeks after Aldo-salt with or without DHT administration (n = 20/group). (**I**) Serum sodium levels in orchiectomized mice administered Aldo-salt with and without DHT administration and with (+) and without (-) AA (n = 1-10/group). (**J**) MAP of the orchiectomized mice one week before and three weeks after Aldo-salt with and without DHT administration (n = 9-11/group). (**K**) Correlation analysis of the internal diameter of the suprarenal aorta and MAP in the orchiectomized mice three weeks after Aldo-salt with and without DHT administration (n = 20/group). (**L**) MAP of the orchiectomized mice with and without DHT and AA (n = 1-10/group). Data were expressed as mean ± SEM and analyzed by two-way ANOVA for multiple comparison tests (**A**–**F**, **I**, **J**, and **L**), two-tailed unpaired *t*-test (**G**), and simple linear regression analysis (**H** and **K**). **, P < 0.05; **, P < 0.01; ***, P < 0.001; ****, P < 0.0001; ns, not significant.*

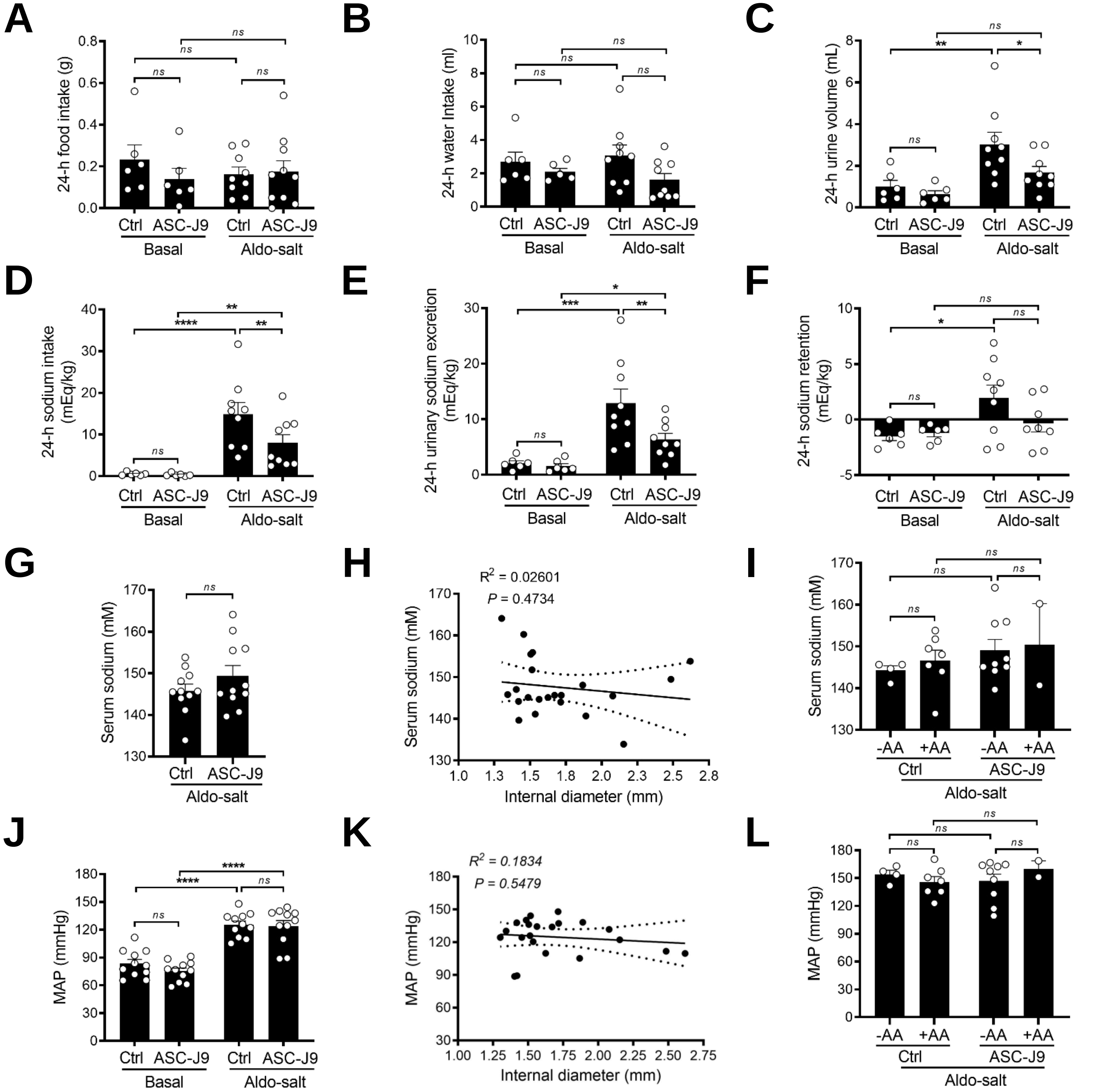

Supplemental Figure 7. Downregulation of androgen receptors with ASC-J9 has little effect on Aldo-salt-induced sodium retention and hypertension. (**A**–**F**) 24-h food intake (A), water intake (B), urine volume (C), sodium intake (D), urinary sodium excretion (E), and sodium retention (F) were determined in 10-month-old male C57BL/6J mice one week before (basal) and three weeks after Aldo-salt with (ASC-J9) and without (Ctrl) ASC-J9 administration (n = 6-10/group). (**G**) Serum sodium levels four weeks after Aldo-salt with and without ASC-J9 administration (n = 11/group). (**H**) Correlation analysis of the internal diameter of the suprarenal aorta and serum sodium levels in mice four weeks after Aldo-salt with and without ASC-J9 administration (n = 22/group). (**I**) Serum sodium levels of the mice administered Aldo-salt with and without ASC-J9 administration and with (+) and without (-) AA (n = 2-9/group). (**J**) MAP of the mice one week before and three weeks after Aldo-salt with and without ASC-J9 administration (n = 11/group). (**K**) Correlation analysis of the internal diameter of the suprarenal aorta and MAP in mice three weeks after Aldo-salt with and without ASC-J9 administration (n = 22/group). (**L**) MAP of the mice with (+) and without (-) AA (n = 2-9/group). Data were expressed as mean ± SEM and analyzed by two-way ANOVA for multiple comparison tests (**A**–**F**, **I**, **J**, and **L**), two-tailed unpaired *t*-test (**G**), and simple linear regression analysis (**H** and **K**). **, $P < 0.05$; **, $P < 0.01$; ***, $P < 0.001$; ****, $P < 0.0001$; ns, not significant.*

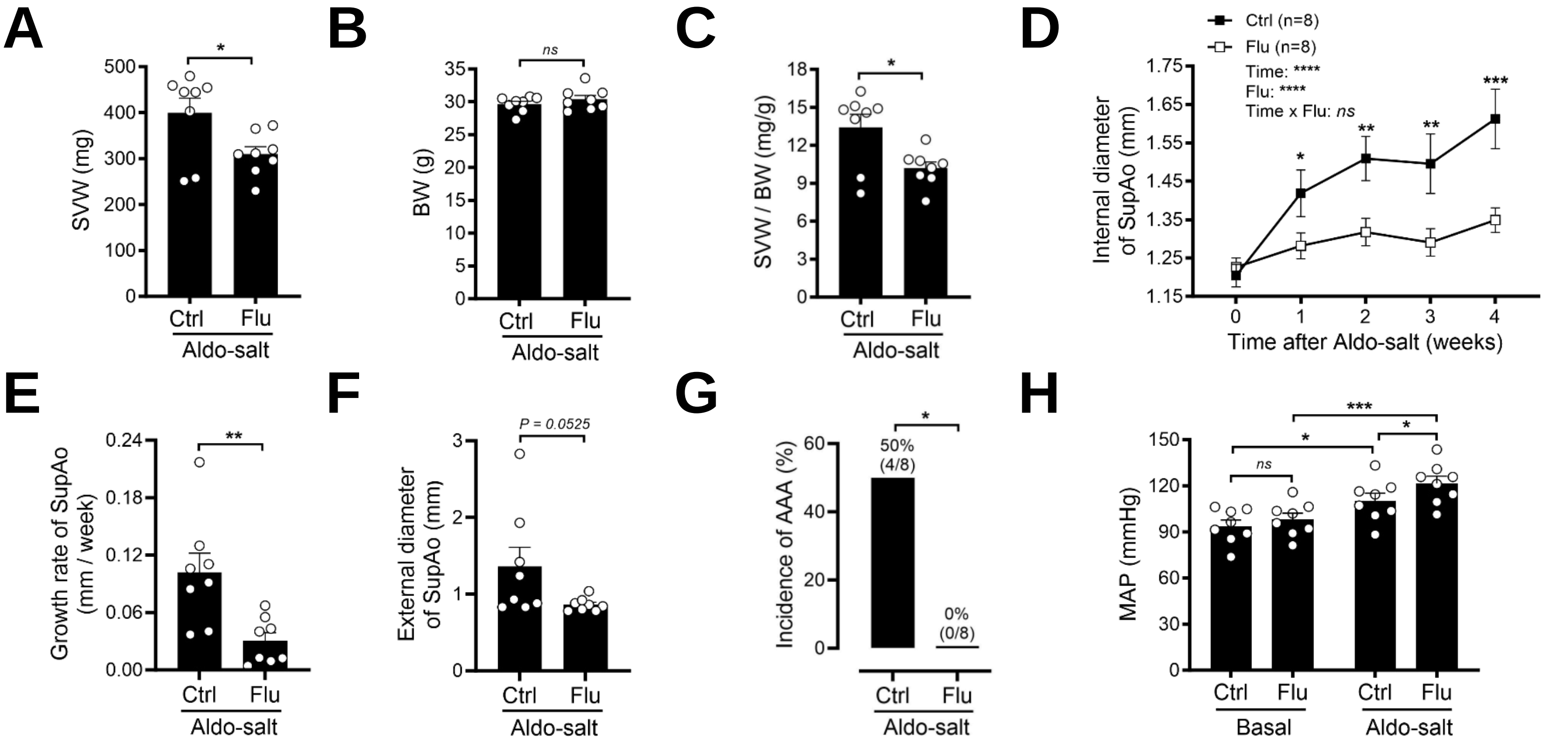

Supplemental Figure 8. Antagonizing androgen receptors with flutamide inhibits Aldo-salt-induced abdominal aortic aneurysms. (**A**–**C**) The SVW, BW, and SVW to BW ratio were measured in 9-10-month-old male C57BL/6J mice four weeks after Aldo-salt with androgen receptor antagonist flutamide (Flu) or vehicle (10% EtOH in corn oil; Ctrl) administration. Flutamide (50 mg/kg/day) and vehicle were delivered to mice via intraperitoneal injection once a day for four weeks (n = 8/group). (**D** and **E**) *In vivo* quantification of the maximal intraluminal diameters (D) and growth rate (E) of the suprarenal aorta by ultrasound one week before (0) and 1–4 weeks after Aldo-salt with flutamide or vehicle administration (n = 8/group). (**F**) *Ex vivo* measurement of the maximal external diameters of the suprarenal aorta by microscopy four weeks after Aldo-salt with flutamide or vehicle administration (n = 8/group). (**G**) The incidence of AAA in mice four weeks after Aldo-salt with flutamide or vehicle administration. (**H**) MAP was measured one week before (basal) and three weeks after Aldo-salt with flutamide or vehicle administration (n = 8/group). The data were expressed as mean ± SEM and analyzed by two-tailed unpaired *t*-test (**A−C**, **E**, and **F**), two-way ANOVA with multiple comparison tests (**D** and **H**), and two-sided Chi-square test (**G**). **, P < 0.05; **, P < 0.01; ***, P < 0.001; ****, P < 0.0001; ns, not significant.*

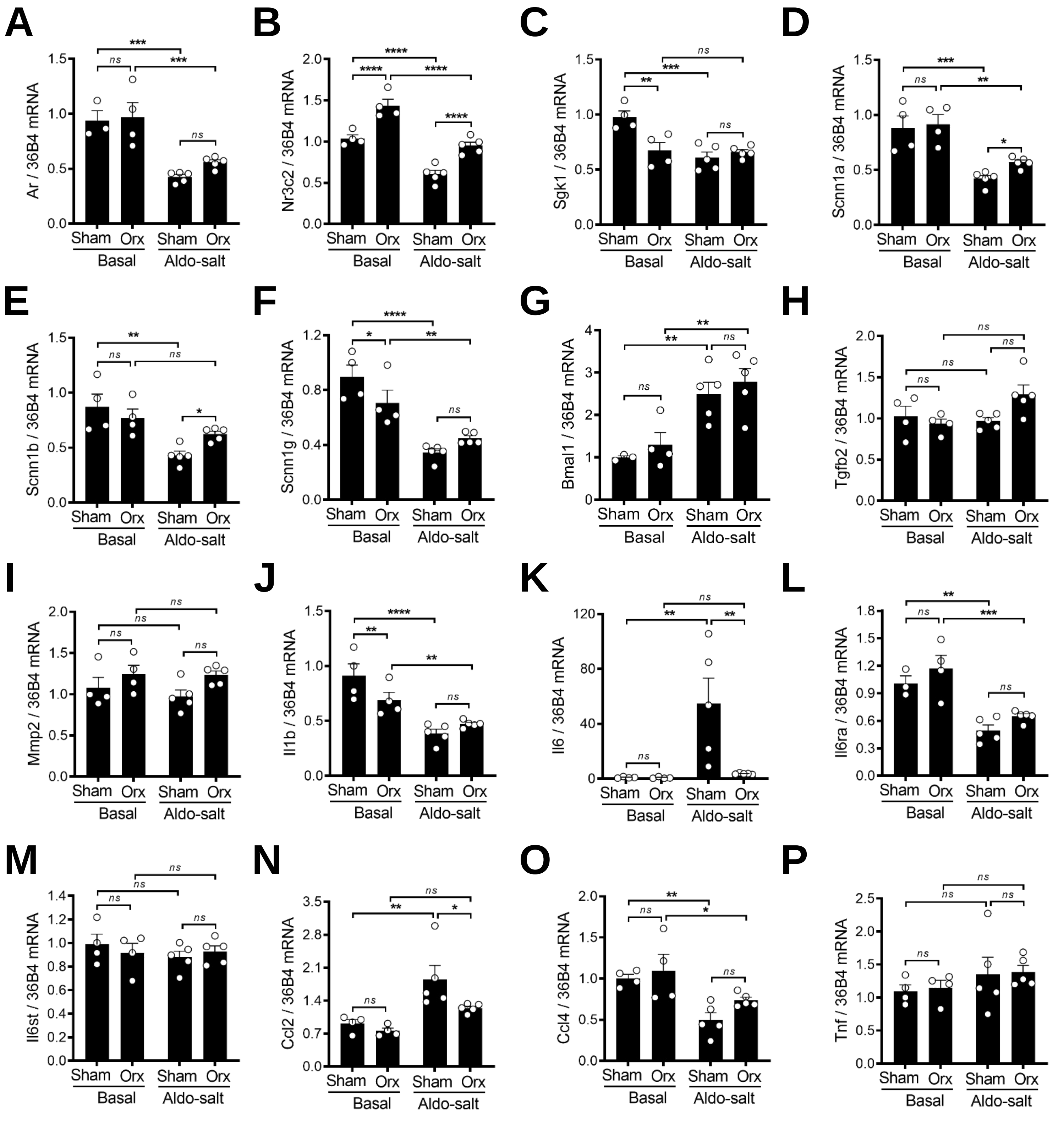

Supplemental Figure 9. Effects of gonadal androgen deprivation on mRNA expression in the aorta in mice administered Aldo-salt. The mRNA expressions in the whole aortas were determined by real-time PCR and normalized to 36B4, a housekeeping gene, in 10-month-old orchiectomized and sham-operated C57BL/6J mice with and without (basal) Aldo-salt administration for ten days. (**A**) *Ar* (androgen receptor). (**B**) *Nr3c2* (mineralocorticoid receptor). (**C**) *Sgk1* (serum and glucocorticoid-regulated Kinase 1). (**D**) *Scnn1a* (sodium channel epithelial 1 subunit alpha; ENaCα). (**E**) *Scnn1b* (Scnn1 subunit beta; Enacβ). (**F**) *Scnn1g* (Scnn1 subunit gamma; ENaCγ). (**G**) *Bmal1*. (**H**) *Tgfb2* (TGFβ2). (**I**) *Mmp2*. (**J**) *Il1b* (IL-1β). (**K**) *Il6* (IL-6). (**L**) *Il6ra* (IL-6 receptor alpha; IL-6Rα). (**M**) *Il6st* (IL-6 signal transducer; IL-6Rβ or gp130). (**N**) *Ccl2* (MCP-1). (**O**) *Ccl4*. (**P**) *Tnf* (TNF-α). N = 3-5/group. Data were expressed as mean ± SEM and analyzed by two-way ANOVA for multiple comparison tests. **, $P < 0.05$; **, $P < 0.01$; ***, $P < 0.001$; ****, $P < 0.0001$; ns, not significant.*

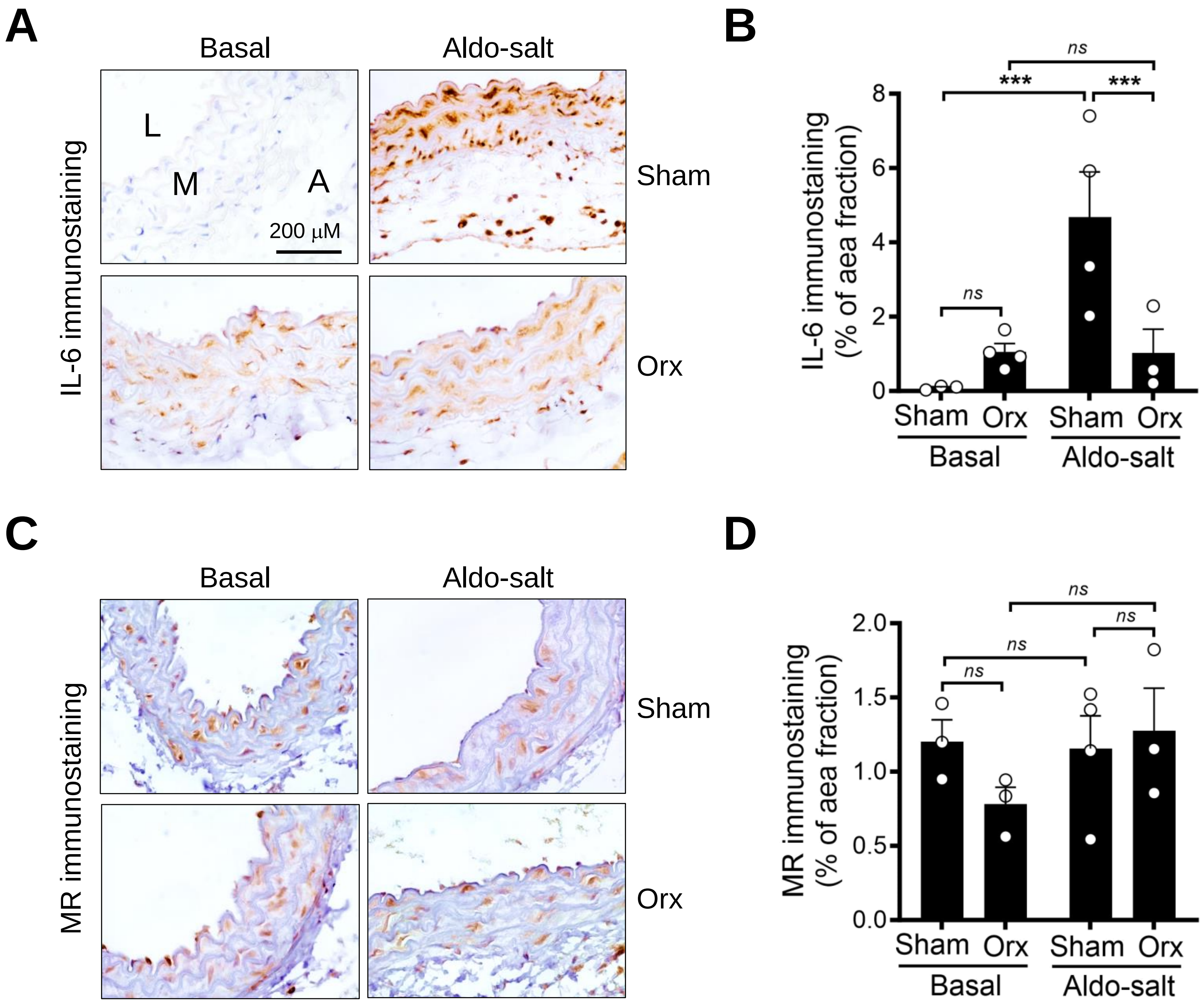

Supplemental Figure 10. Gonadal androgen deprivation affects the IL-6 but not MR protein expression in the suprarenal aorta before and after Aldo-salt administration. (**A** and **B**) Representative immunostaining and quantitative data of IL-6 in the cross-sections of the suprarenal aorta in 10-month-old orchiectomized and sham-operated C57BL/6J mice with and without (basal) 10-day Aldo-salt administration (n = 3-4/group). (**C** and **D**) Representative **i**mmunostaining and quantitative data of MR in the cross-sections of the suprarenal aorta in 10-month-old orchiectomized and sham-operated C57BL/6J mice with and without (basal) 10-day Aldo-salt administration (n = 3-4/group). L, lumen. M, media. A, adventitia. The percentage of areas fraction = (positive areas / areas of fields of view) x 100%. The data were calculated from three fields of view randomly photographed per aortic section per mouse. The data were expressed as mean ± SEM and analyzed by two-way ANOVA with multiple comparison tests. ***, P < 0.01; ns, not significant.*

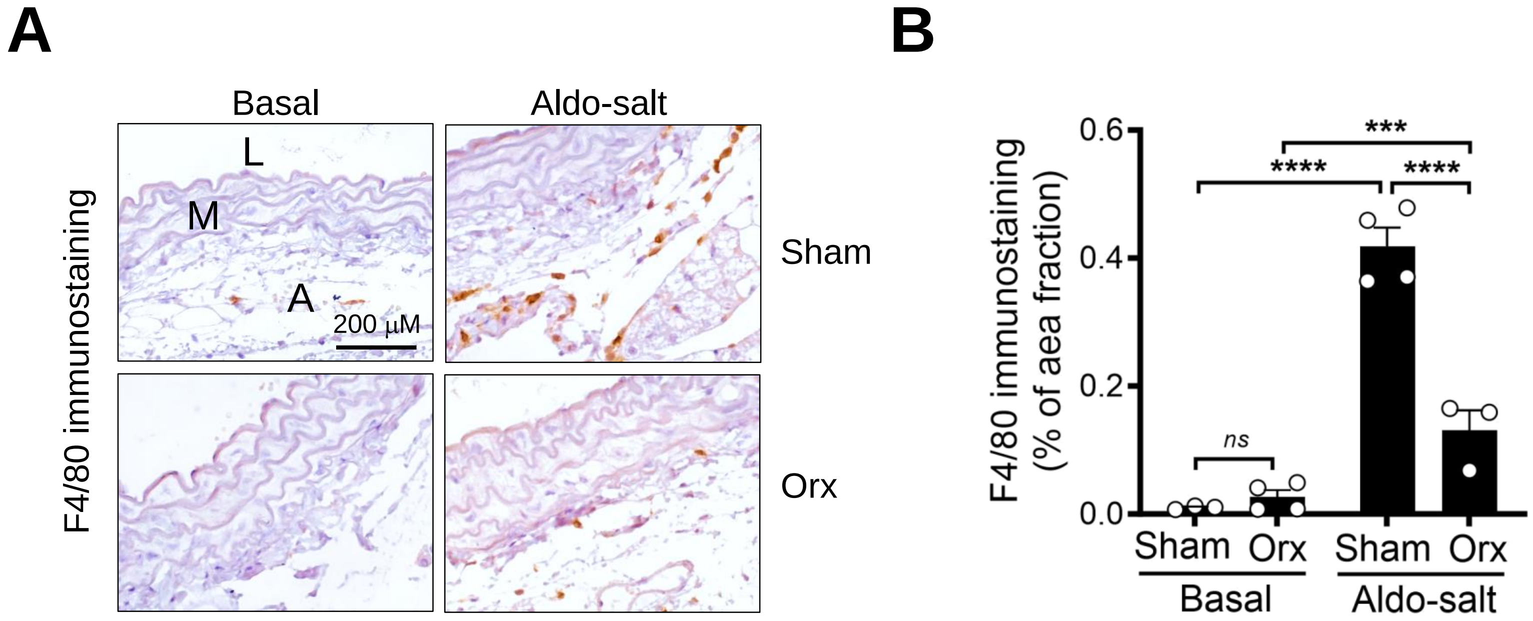

Supplemental Figure 11. Gonadal androgen deprivation abolishes Aldo-salt-induced macrophage infiltration in the abdominal aorta. Representative **i**mmunostaining (**A**) and quantitative data (**B**) of macrophage immunostaining with anti-F4/80 Ab in the cross-sections of the suprarenal aorta in 10-month-old C57BL/6J mice with orx or sham operation ten days after Aldo-salt relative to those without Aldo-salt (basal) administration (n = 3-4/group). L, lumen. M, media. A, adventitia. The percentage of areas fraction = (the F4/80 positive area / the area of fields of view) x 100%. The data were calculated from three fields of view randomly photographed per aortic section per mouse. The data were expressed as mean ± SEM and analyzed by two-way ANOVA with multiple comparison tests ***, $P < 0.01$; ****, $P < 0.0001$; ns, not significant.*

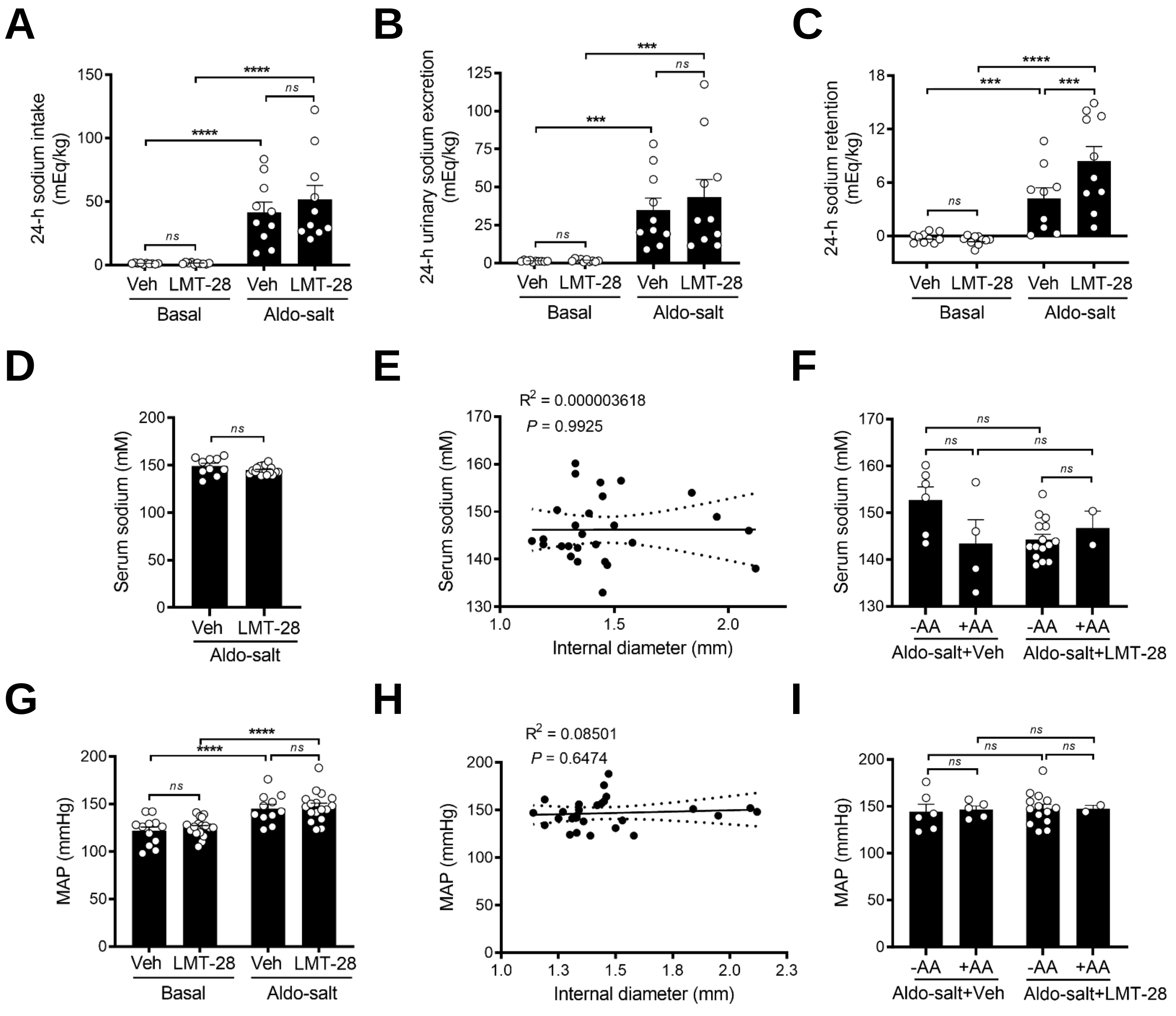

Supplemental Figure 12. LMT-28 has no effect on Aldo-salt-induced renal sodium retention and hypertension. (**A**–**C**) 24-h sodium intake (A), urinary sodium excretion (B), and sodium retention (C) were measured in 10-month-old male C57BL/6J mice one week before (basal) and three weeks after Aldo-salt with LMT-28 or vehicle (Veh) administration (n = 10/group). (**D**) Serum sodium levels of the mice four weeks after Aldo-salt with LMT-28 or vehicle administration (n = 10-17/group). (**E**) Correlation analysis of the internal diameter of the suprarenal aorta and serum sodium levels in mice four weeks after Aldo-salt with LMT-28 or vehicle administration (n = 27/group). (**F**) Serum sodium levels of the mice with and without AA four weeks after Aldo-salt with LMT-28 or vehicle administration (n = 2-15/group). (**G**) MAP was measured one week before (basal) and three weeks after Aldo-salt with LMT-28 or vehicle administration (n = 9-18/group). (**H**) Correlation analysis of the MAP and internal diameter of the suprarenal aorta four weeks after Aldo-salt with LMT-28 or vehicle administration (n = 27/group). (**I**) MAP of the mice administered Aldo-salt with LMT-28 or vehicle administration and with (+) and without (-) AA (n = 2-15/group). Data were expressed as mean ± SEM and analyzed by two-way ANOVA for multiple comparison tests (**A**, **B**, **C**, **F**, **G**, and **I**), two-tailed unpaired *t*-test (**D**), and simple linear regression analysis (**E** and **H**). ***, P < 0.01; ***, P < 0.001; ****, P < 0.0001; ns, not significant.*

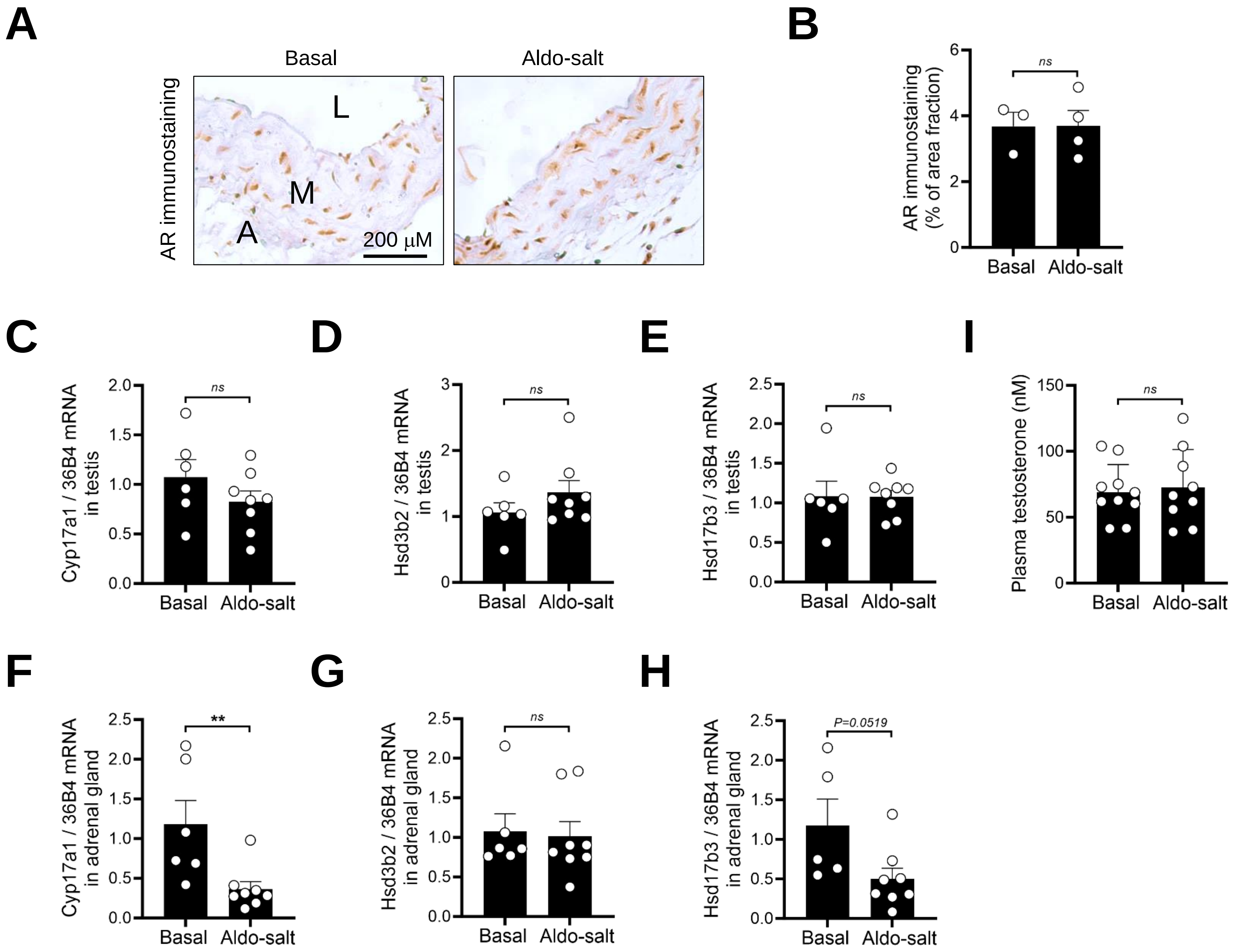

Supplemental Figure 13. Effect of Aldo-salt on androgen receptor and androgen synthesis expression in the aorta, testis, and adrenal gland in mice administered Aldo-salt. (**A** and **B**) Representative immunostaining (A) and quantitative data (B) of androgen receptor (AR) protein expression in the cross-section of the abdominal aorta in 10-month-old male C57BL/6J mice ten days after Aldo-salt relative to those without Addo-salt (basal) administration (n = 3-4/group). (**C−H**) The mRNA expressions in the testis and adrenal gland were determined by real-time PCR and normalized to 36B4 in 10-month-old male C57BL/6J mice with and without (basal) 10-day Aldo-salt administration. Cytochrome P450, family 17, subfamily a, polypeptide 1 (Cyp17a1; C and F), hydroxysteroid (17-beta) dehydrogenase 3 (Hsd17b3; D and G), and hydroxy-delta-5-steroid dehydrogenase, 3 beta- and steroid delta-isomerase 2 (Hsd3b2; E and H) mRNA expressions in the testis (C−E; n = 6-8/group) and adrenal gland (F−H; n = 5-8/group). (**I**) Plasma testosterone levels in mice with and without (basal) 10-day Aldo-salt administration (n = 9-10/group). One symbol in the figures represents one mouse. The data were expressed as mean ± SEM and analyzed by a two-tailed unpaired *t*-test; ***, P < 0.01; ns, not significant.*

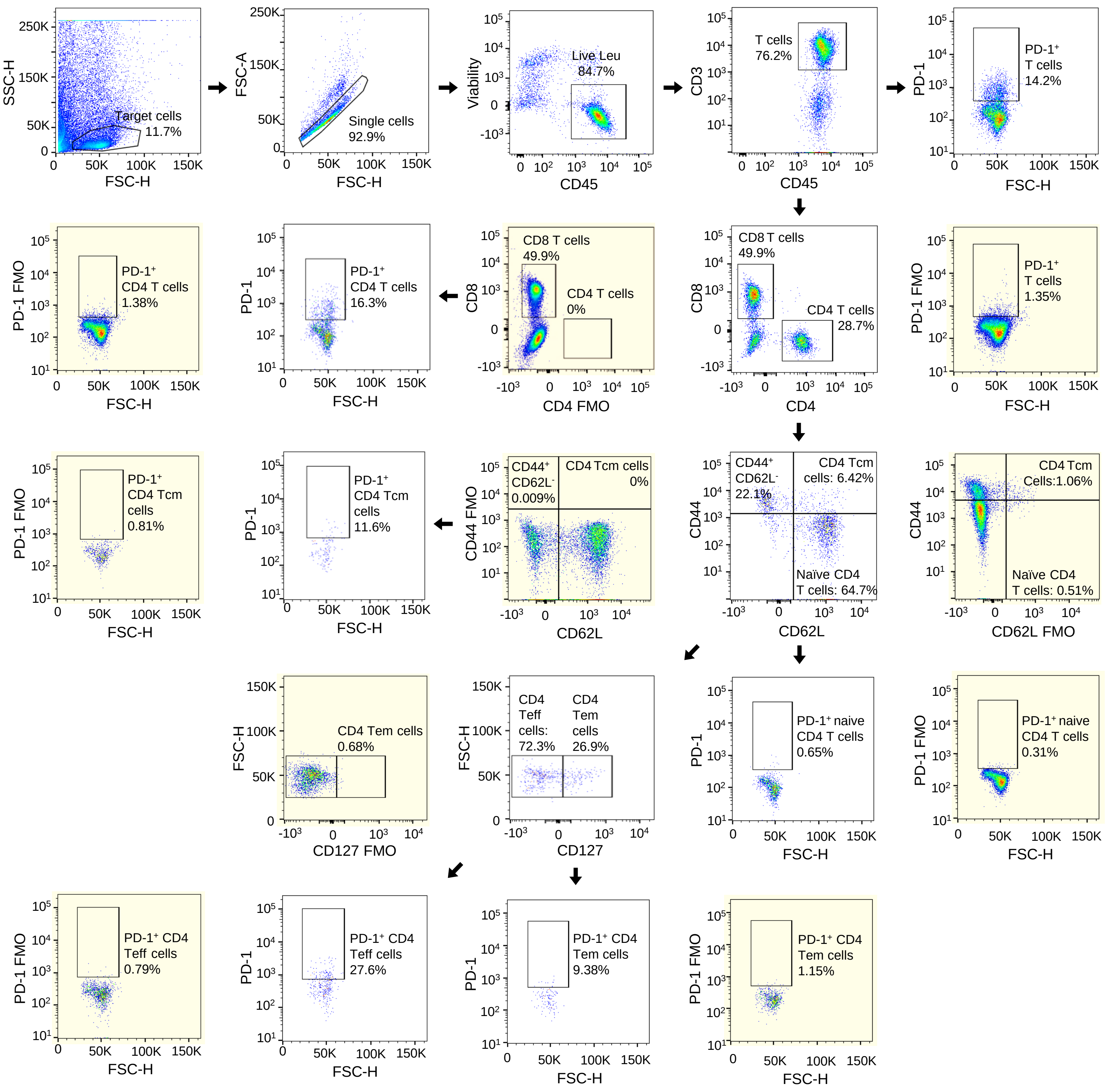

Supplemental Figure 14. Gating strategy of the flow cytometry analysis to identify CD4 T cell subsets in the mouse aorta. Single cells were prepared from the whole aortas of mice and gated in by a forward scatter height (FSC-H) *vs.* side scatter height (SSC-H) pseudocolor plot. The aortic single cells were gated on CD45 *vs.* live/dead cell staining to select viable leukocytes (Leu; CD45$^+$) and then gated on CD3, CD4, and CD8 to identify T cells (CD45$^+$CD3$^+$), CD4 helper T cells (CD45$^+$CD3$^+$CD4$^+$), and CD8 cytotoxic T cells (CD45$^+$CD3$^+$CD8$^+$), respectively. The CD4 T cells were then gated on CD44 and CD62L to identify CD4 central memory T cells (CD4Tcm; CD45$^+$CD3$^+$CD4$^+$CD44$^+$CD62L$^+$), CD4 naïve T cells (CD45$^+$CD3$^+$CD4$^+$CD44$^-$CD62L$^+$), and CD4 CD44$^+$CD62L$^-$ T cells, respectively. The CD44$^+$CD62L$^-$ T cells were then gated on CD127 to identify CD4 effector T cells (CD4 Teff; CD45$^+$CD3$^+$CD4$^+$CD44$^+$CD62L$^-$CD127$^-$) and CD4 effector memory T cells (CD4 Tem; CD45$^+$CD3$^+$CD4$^+$CD44$^+$CD62L$^-$CD127$^+$), respectively. All T cell subsets were gated on PD-1 to identify PD-1$^+$ T cell subsets. The single cells were also prepared from the spleen and analyzed by flow cytometry with fluorescence minus one (FMO; labeled with a yellow background in pseudocolor plots) to define the gating boundaries and ensure the specificity of antibodies.

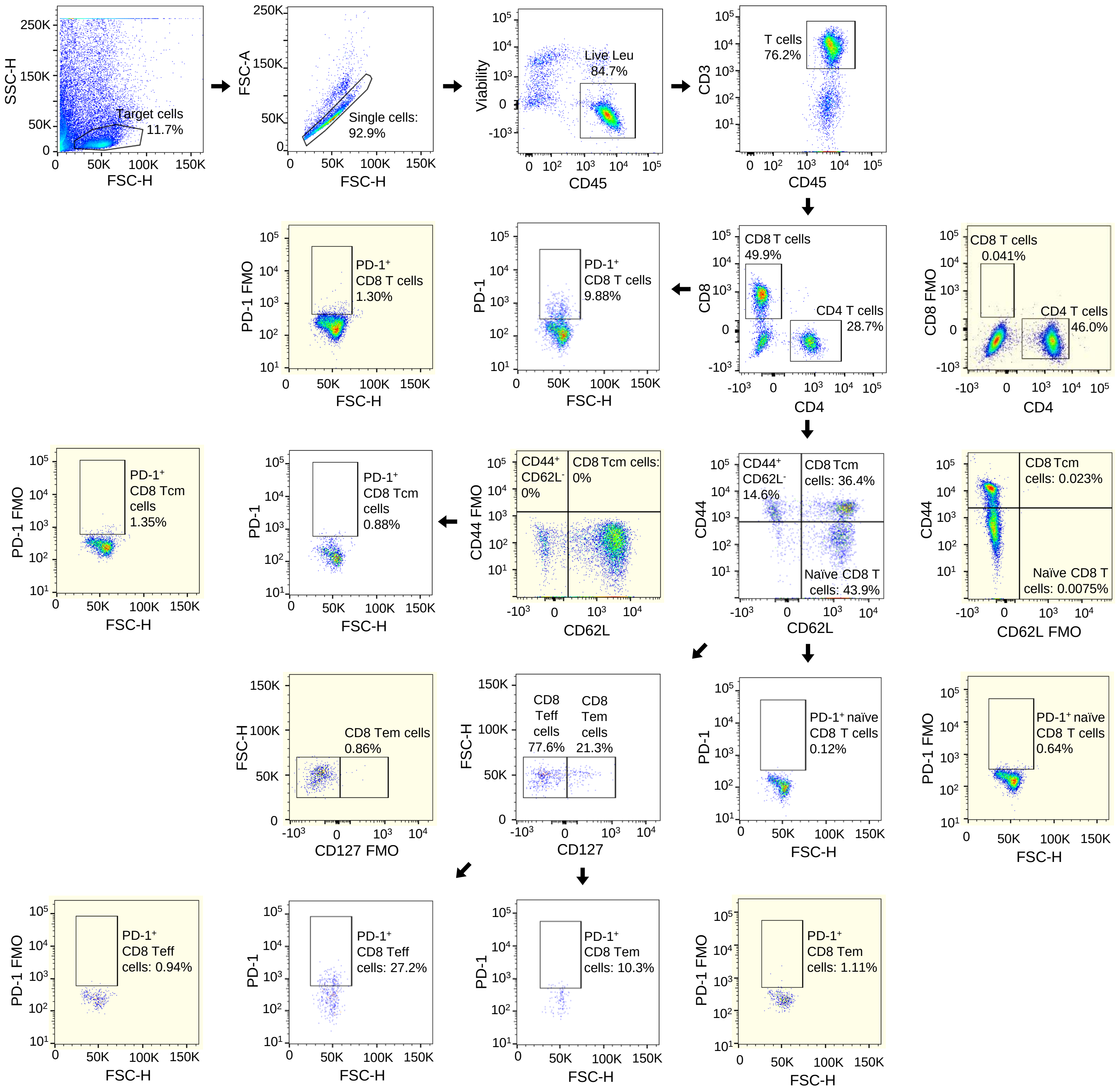

Supplemental Figure 15. Gating strategy of the flow cytometry analysis to identify CD8 T cell subsets in the mouse aorta. Single cells were prepared from the whole aortas of mice and gated in by a forward scatter height (FSC-H) *vs.* side scatter height (SSC-H) pseudocolor plot. The aortic single cells were gated on CD45 *vs.* live/dead cell staining to select viable leukocytes (Leu; $CD45^+$) and then gated on CD3, CD4, and CD8 to identify total T cells ($CD45^+CD3^+$), CD4 helper T cells ($CD45^+CD3^+CD4^+$), and CD8 cytotoxic T cells ($CD45^+CD3^+CD8^+$), respectively. The CD8 T cells were then gated on CD44 and CD62L to identify CD8 central memory T cells (CD8Tcm; $CD45^+CD3^+CD8^+CD44^+CD62L^+$), CD8 naïve T cells ($CD45^+CD3^+CD8^+CD44^-CD62L^+$), and CD8 $CD44^+CD62L^-$ T cells, respectively. The $CD44^+CD62L^-$ T cells were then gated on CD127 to identify CD8 effector T cells (CD8 Teff; $CD45^+CD3^+CD8^+CD44^+CD62L^-CD127^-$) and CD8 effector memory T cells (CD8 Tem; $CD45^+CD3^+CD8^+CD44^+CD62L^-CD127^+$), respectively. All T cell subsets were gated on PD-1 to identify PD-1^+ T cell subsets. The single cells were also prepared from the spleen and analyzed by flow cytometry with fluorescence minus one (FMO; labeled with a yellow background in pseudocolor plots) to define the gating boundaries and ensure the specificity of antibodies.

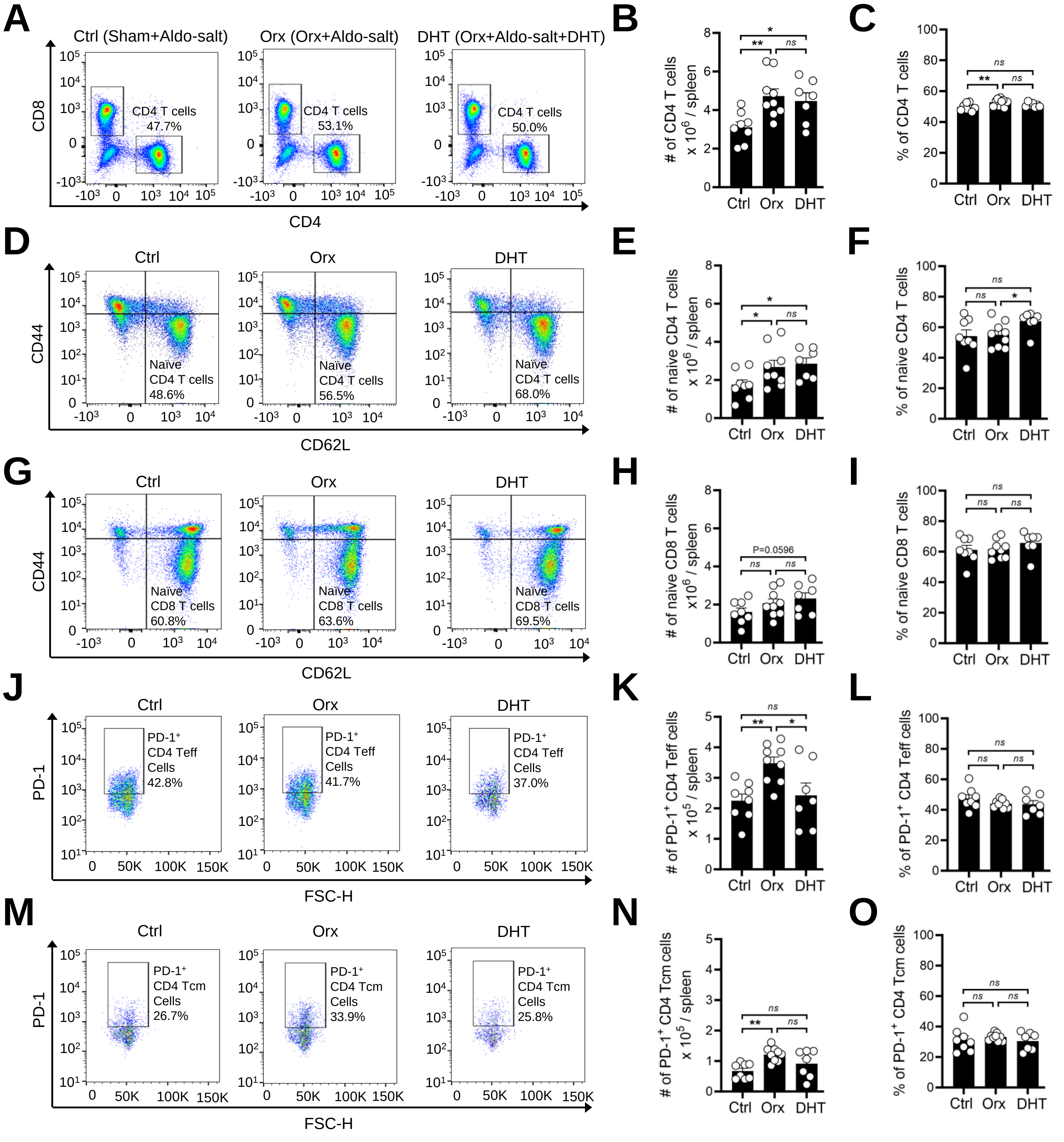

Supplemental Figure 16. Flow cytometry analysis of T cell subsets in the spleen in orchiectomized and sham-operated mice ten days after Aldo-salt with or without DHT administration. Representative pseudocolor plots and quantitative data of the flow cytometry analysis of the total number (#) and percentage (%) of splenic CD4 T cells ($CD45^+CD3^+CD4^+$; % in total T cells; **A−C**), naïve CD4 T cells ($CD45^+CD3^+CD4^+CD44^-CD62L^+$; % in total CD4 T cells; **D−F**), naïve CD8 T cells ($CD45^+CD3^+CD8^+CD44^-CD62L^+$; % in total CD8 T cells; **G−I**), $PD\text{-}1^+$ effector CD4 T cells ($PD\text{-}1^+$CD4 Teff; $CD45^+CD3^+CD4^+CD44^+CD62L^-CD127^-PD\text{-}1^+$; % in total CD4 Teff cells; **J−L**), and $PD\text{-}1^+$ central memory CD4 T cells ($PD\text{-}1^+$CD4 Tcm; $CD45^+CD3^+CD4^+CD44^+CD62L^+PD\text{-}1^+$; % in total CD4 Tcm cells; **M−O**) in the whole spleen in 9-10-month-old male C57BL/6J mice with orchiectomy (Orx) or sham operation (Ctrl) 10 days after Aldo-salt with or without DHT administration (n = 7-9/group). One symbol in the figures represents the cells of one mouse's spleen. The data were expressed as mean ± SEM and analyzed by one-way ANOVA with multiple comparison tests. **, $P < 0.05$; **, $P < 0.01$; ns, not significant.*

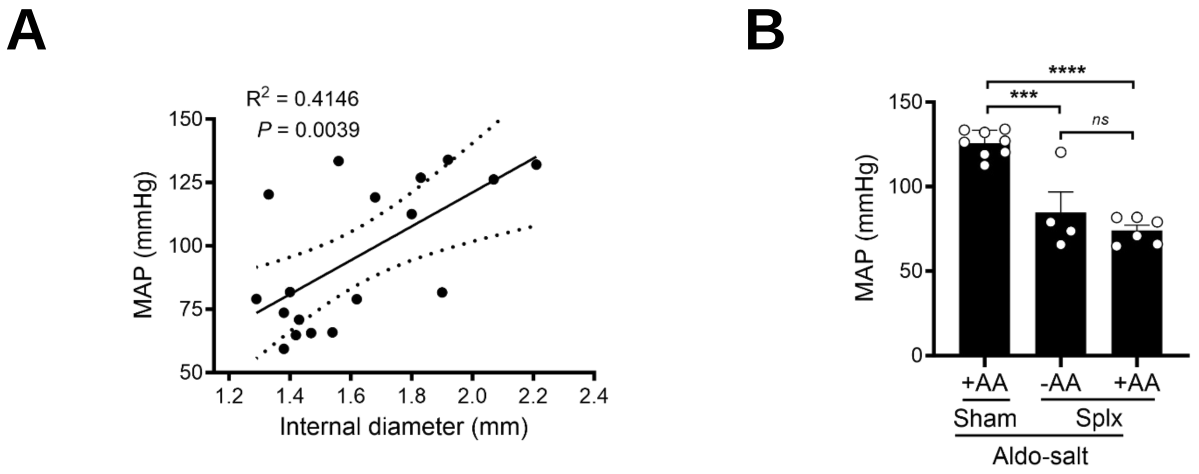

Supplemental Figure 17. Blood pressure, aortic dilation, and aortic aneurysms in splenectomized mice administered Aldo-salt. (**A**) Correlation analysis of MAP and the internal diameter of the suprarenal aorta in 11-13-month-old male C57BL/6J mice with splenectomy (splx) and sham operation three weeks after Aldo-salt administration (n = 18/group). (**B**) MAP in splenectomized and sham-operated mice administered Aldo-salt with (+) and without (-) aortic aneurysms (n = 4-8/group). Data were expressed as mean ± SEM and analyzed by simple linear regression analysis (**A**) and one-way ANOVA for multiple comparison tests (**B**). ****, $P < 0.001$; ****, $P < 0.0001$; ns,. not significant.*

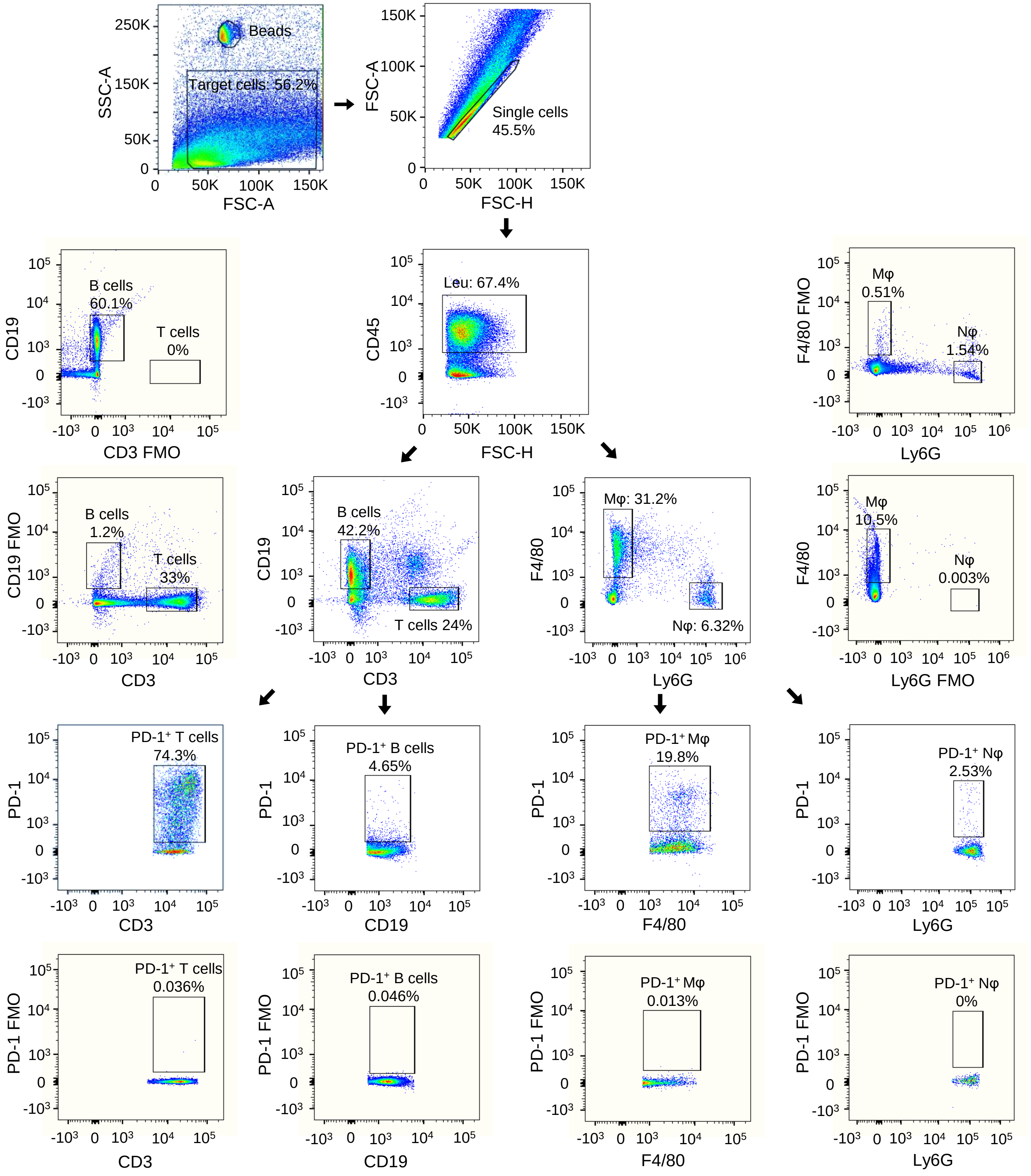

Supplemental Figure 18. Gating strategy of the flow cytometry analysis to identify PD-1$^+$ T cells, PD-1$^+$ B cells, PD-1$^+$ macrophages, and PD-1$^+$ neutrophils in the mouse aorta. Single cells were prepared from the whole aortas and gated in by a forward scatter area (FSC-A) *vs.* side scatter area (SSC-A) pseudocolor plot. Single cells were gated on CD45$^+$ to select leukocytes (Leu; CD45$^+$) and then gated on CD3$^+$, CD19$^+$, F4/80$^+$, and Ly6G$^+$ to identify T cells (CD45$^+$CD3$^+$), B cells (CD45$^+$CD19$^+$), macrophages (Mφ; CD45$^+$F4/80$^+$), and neutrophils (Nφ; CD45$^+$Ly6G$^+$), respectively. The cells were then gated on PD-1$^+$ to identify PD-1$^+$ T cells (CD45$^+$CD3$^+$PD-1$^+$), PD-1$^+$ B cells (CD45$^+$CD19$^+$PD-1$^+$), PD-1$^+$ macrophages (CD45$^+$F4/80$^+$PD-1$^+$), and PD-1$^+$ neutrophils (CD45$^+$Ly6G$^+$ PD-1$^+$), respectively. The single cells were also prepared from lymph nodes and analyzed by flow cytometry with fluorescence minus one (FMO) (labeled with a yellow background in pseudocolor plots) to define the gating boundaries and ensure the specificity of antibodies.

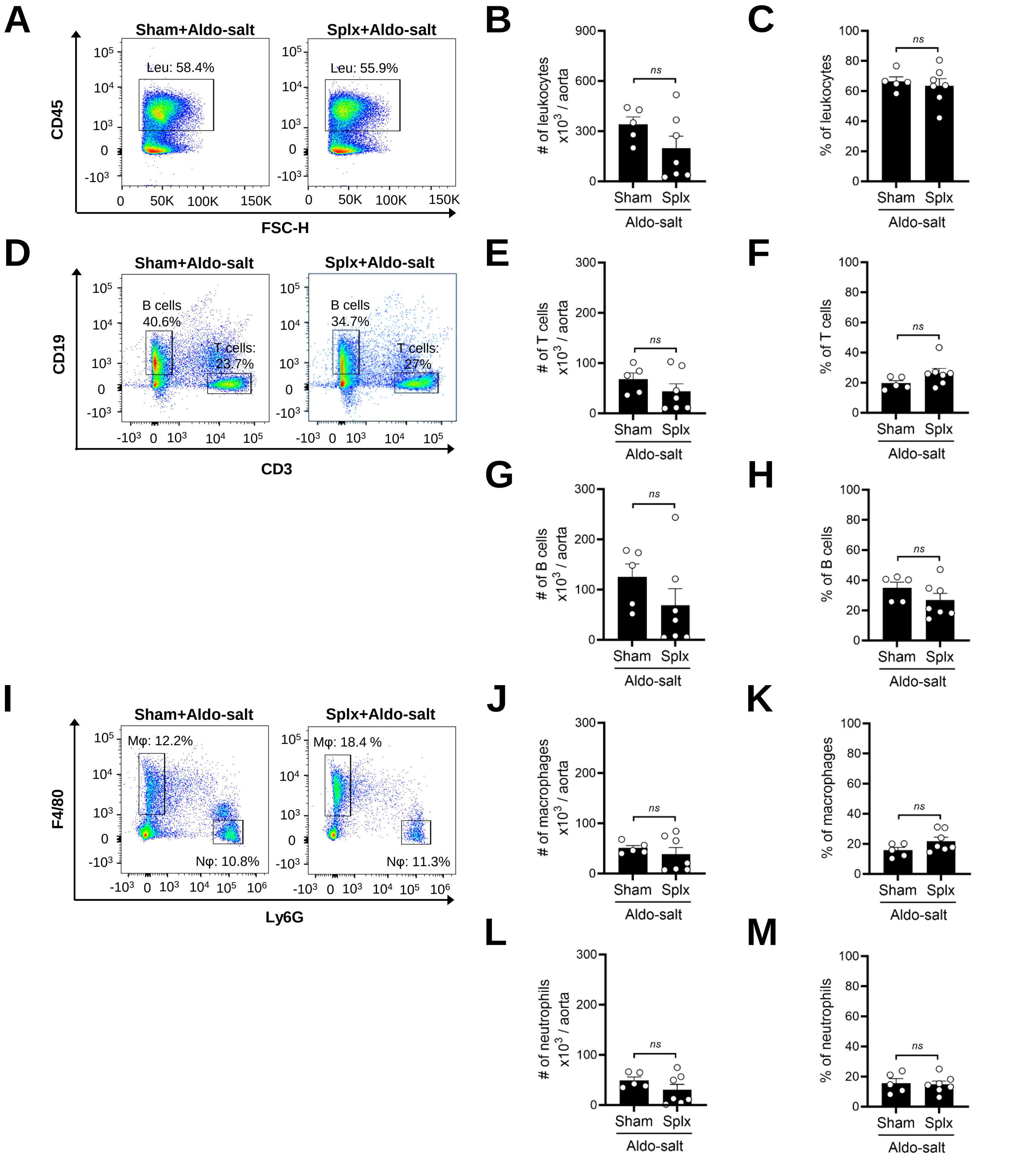

Supplemental Figure 19. Splenectomy does not alter leukocytes, T cells, B cells, macrophages, and neutrophils in the aorta in mice administered Aldo-salt. Representative pseudocolor plots and quantitative data of the flow cytometry analysis of the total number (#) and percentage (%) of leukocytes (Leu; $CD45^+$; % in total single aortic cells; **A−C**), T cells ($CD45^+CD3^+$; % in total leukocytes; **D−F**), B cells ($CD45^+CD19^+$; % in total leukocytes; **D**, **G**, and **H**), macrophages (Mφ; $CD45^+F4/80^+$; % in total leukocytes; **I−K**), and neutrophils (Nφ; $CD45^+Ly6G^+$; % in total leukocytes; **I**, **L**, and **M**) in the whole aortas in 11-13-month-old male C57BL/6J mice with splenectomy (splx) or sham operation four weeks after Aldo-salt administration (n = 5-7/group). One symbol in the figures represents the cells of one mouse aorta. The data were expressed as mean ± SEM and analyzed by a two-tailed unpaired *t*-test. *ns, not significant.*

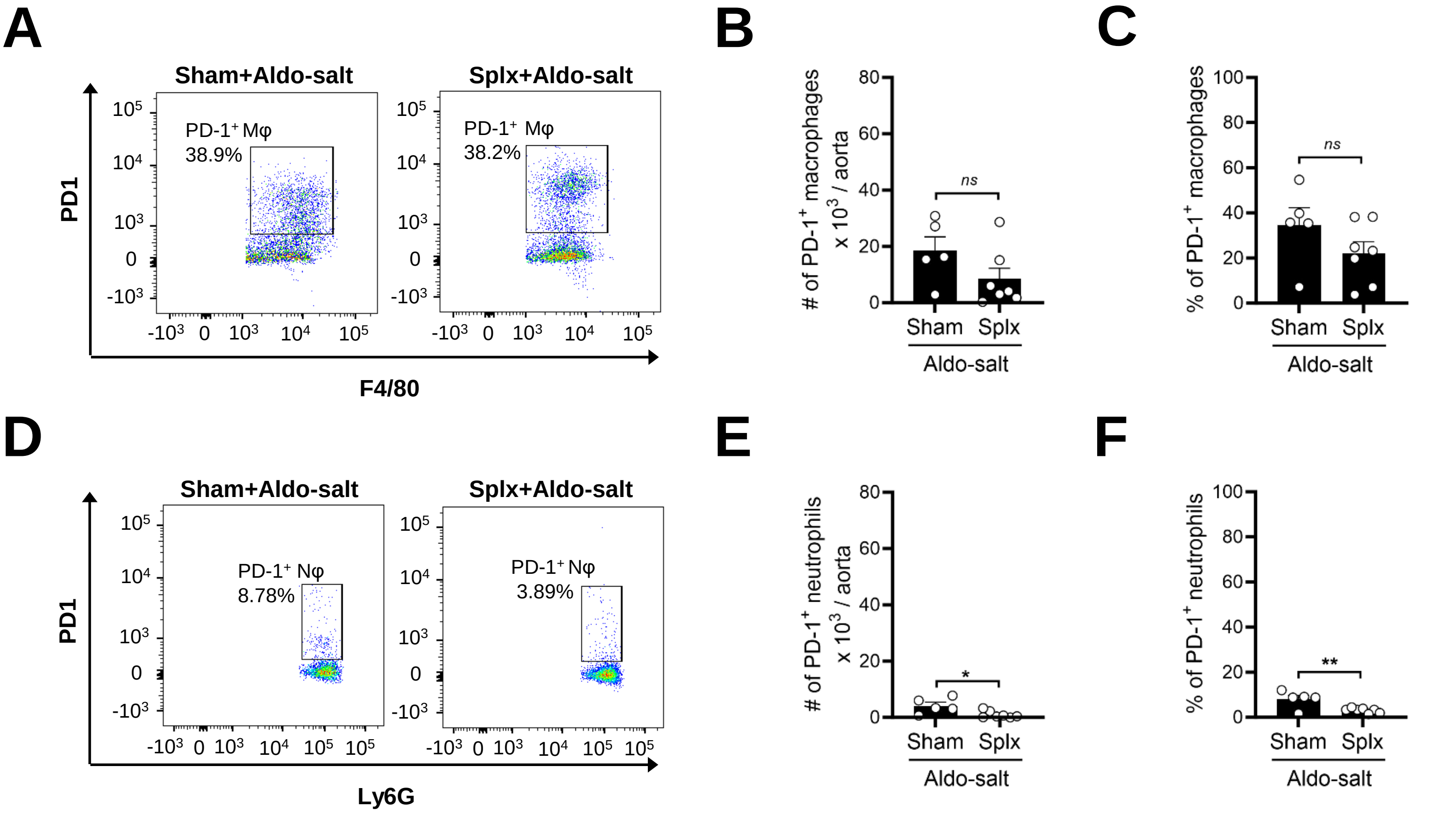

Supplemental Figure 20. Effects of splenectomy on PD-1$^+$ macrophages and PD-1$^+$ neutrophils in the aorta of mice administered Aldo-salt. Representative pseudocolor plots and quantitative data of the flow cytometry analysis of the total numbers (#) and percentages (%) of PD-1$^+$ macrophages (PD-1$^+$Mφ; CD45$^+$F4/80$^+$PD-1$^+$; % in total Mφ; **A**−**C**) and PD-1$^+$ neutrophils (PD-1$^+$Nφ; CD45$^+$Ly6G$^+$PD-1$^+$; % in total Nφ; **D**−**F**) in the whole aortas of 11-13-month-old male C57BL/6J mice with splx or sham-operation four weeks after Aldo-salt administration (n = 5-7/group). One symbol shown in the figures represents cells in one whole mouse's aorta. The data were expressed as mean ± SEM and analyzed by a two-tailed unpaired *t*-test. **, P < 0.05; **, P < 0.01. ns, not significant.*

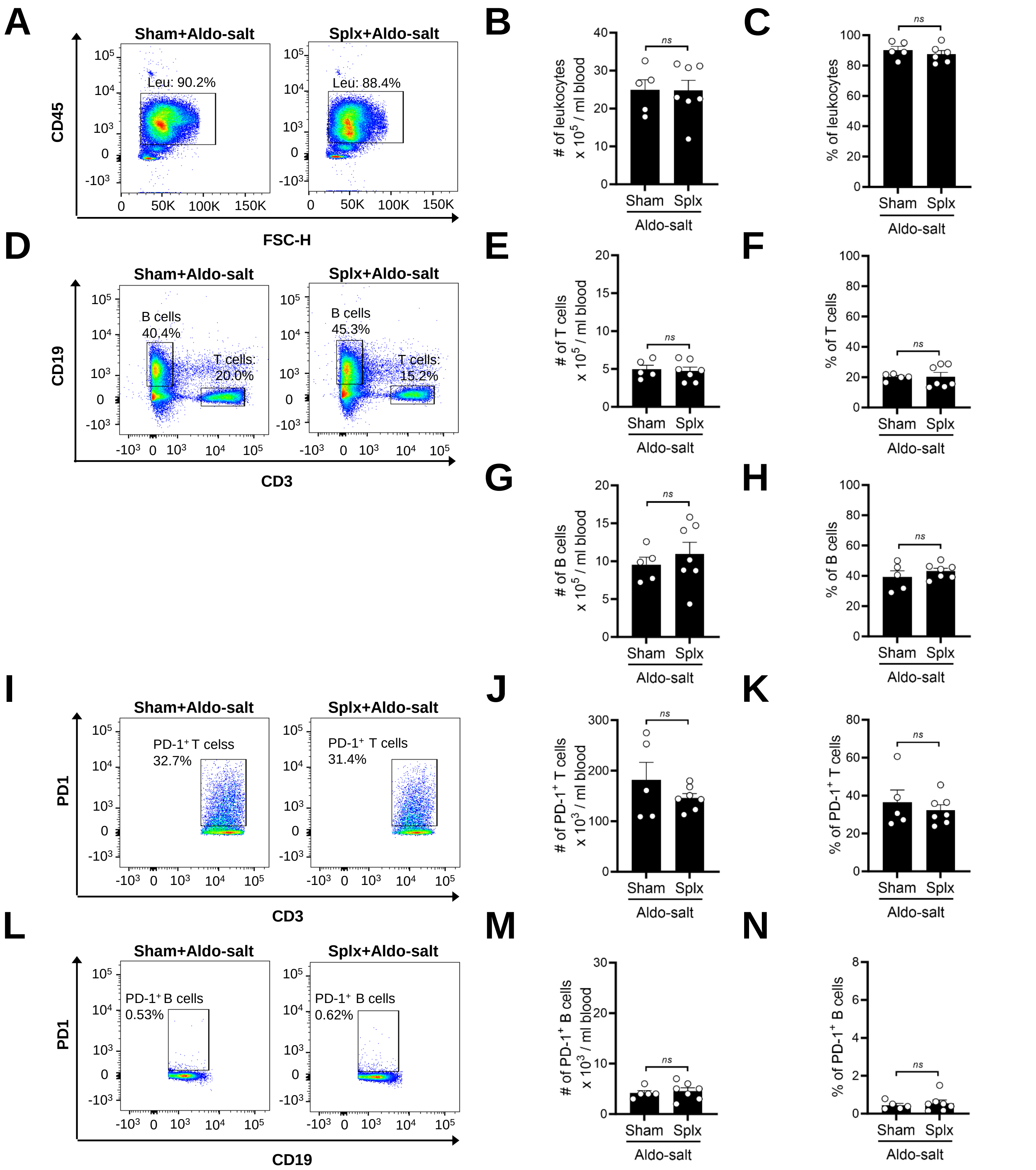

Supplemental Figure 21. Splenectomy has no effect on PD-1⁺ T cells and PD-1⁺ B cells in the blood in mice administered Aldo-salt. Representative pseudocolor plots and quantitative data of the flow cytometry analysis of the total number (#) and percentage (%) of leukocytes (Leu; $CD45^+$; % in total blood cells; **A−C**), T cells ($CD45^+CD3^+$; % in total leukocytes; **D−F**), B cells ($CD45^+CD19^+$; % in total leukocytes; **D**, **G**, and **H**), PD-1⁺ T cells ($CD45^+CD3^+PD\text{-}1^+$; % in total T cells; **I−K**), and PD-1⁺ B cells ($CD45^+CD19^+PD\text{-}1^+$; % in total B-cells; **L−N**) in the blood in 11-13-month-old male C57BL/6J mice with splx or sham operation four weeks after Aldo-salt administration (n = 5-7/group). One symbol represents the cells of one mouse's blood. The data were expressed as mean ± SEM and analyzed by a two-tailed unpaired *t*-test. *ns, not significant.*

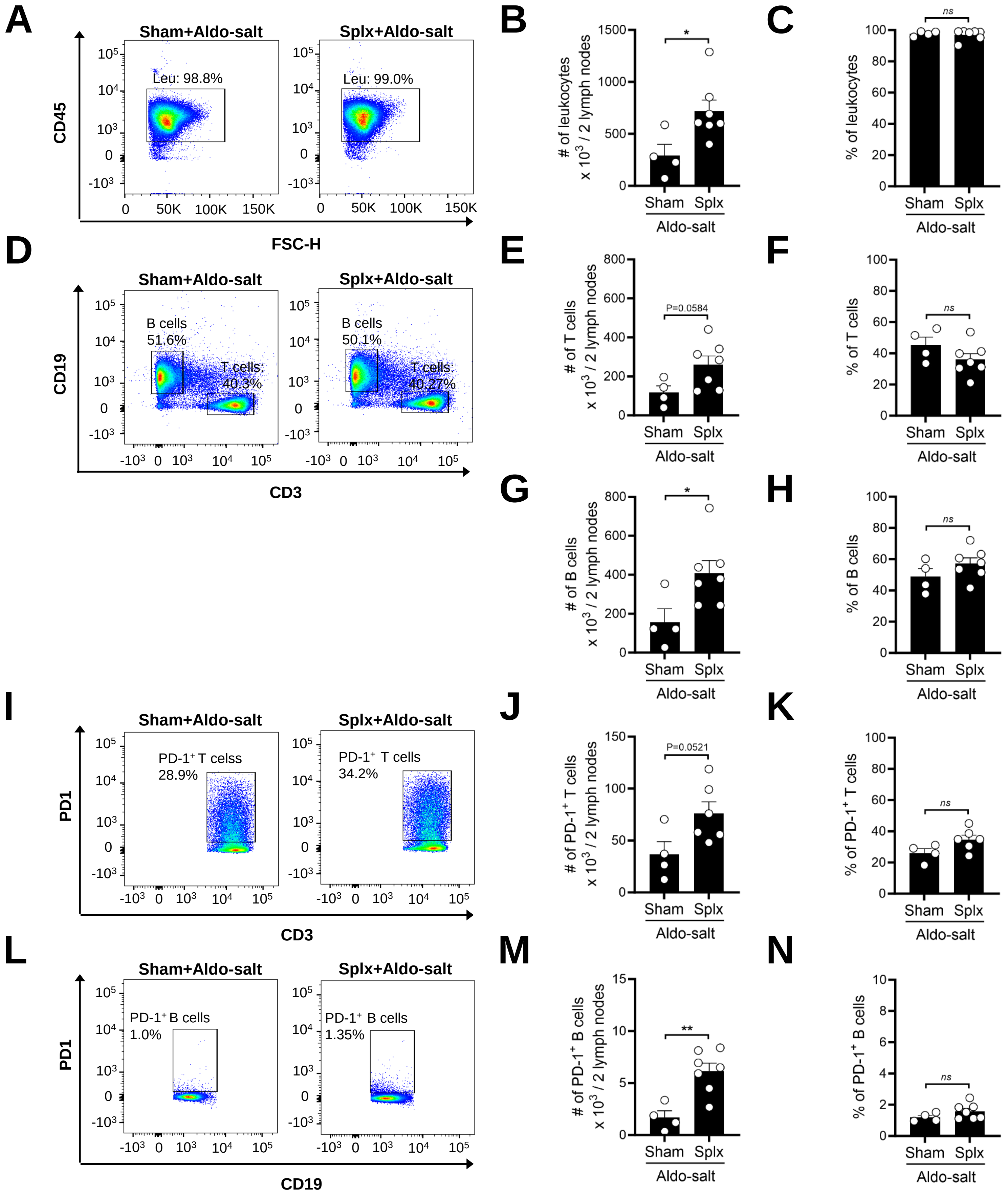

Supplemental Figure 22. Splenectomy augments PD-1$^+$ T cells and PD-1$^+$ B cells in the periaortic lymph nodes in mice administered Aldo-salt. Representative pseudocolor plots and quantitative data of the flow cytometry analysis of the total number (#) and percentage (%) of leukocytes (Leu; CD45$^+$; % in total lymph node cells; **A−C**), T cells (CD45$^+$CD3$^+$; % in total leukocytes; **D−F**), B cells (CD45$^+$CD19$^+$; % in total leukocytes; **D**, **G**, and **H**), PD-1$^+$ T cells (CD45$^+$CD3$^+$PD-1$^+$; % in total T cells; **I−K**), and), and PD-1$^+$ B cells (CD45$^+$CD19$^+$PD-1$^+$; % in total cells; **L−N**) in two periaortic lymph nodes in 11-13-month-old male C57BL/6J mice with splenectomy (splx) or sham operation four weeks after Aldo-salt administration (n = 4-7/group). One symbol in the figures represents the cells of one mouse's two periaortic lymph nodes. The data were expressed as mean ± SEM and analyzed by a two-tailed unpaired *t*-test. **, P < 0.05; **, P < 0.01; ns, not significant.*

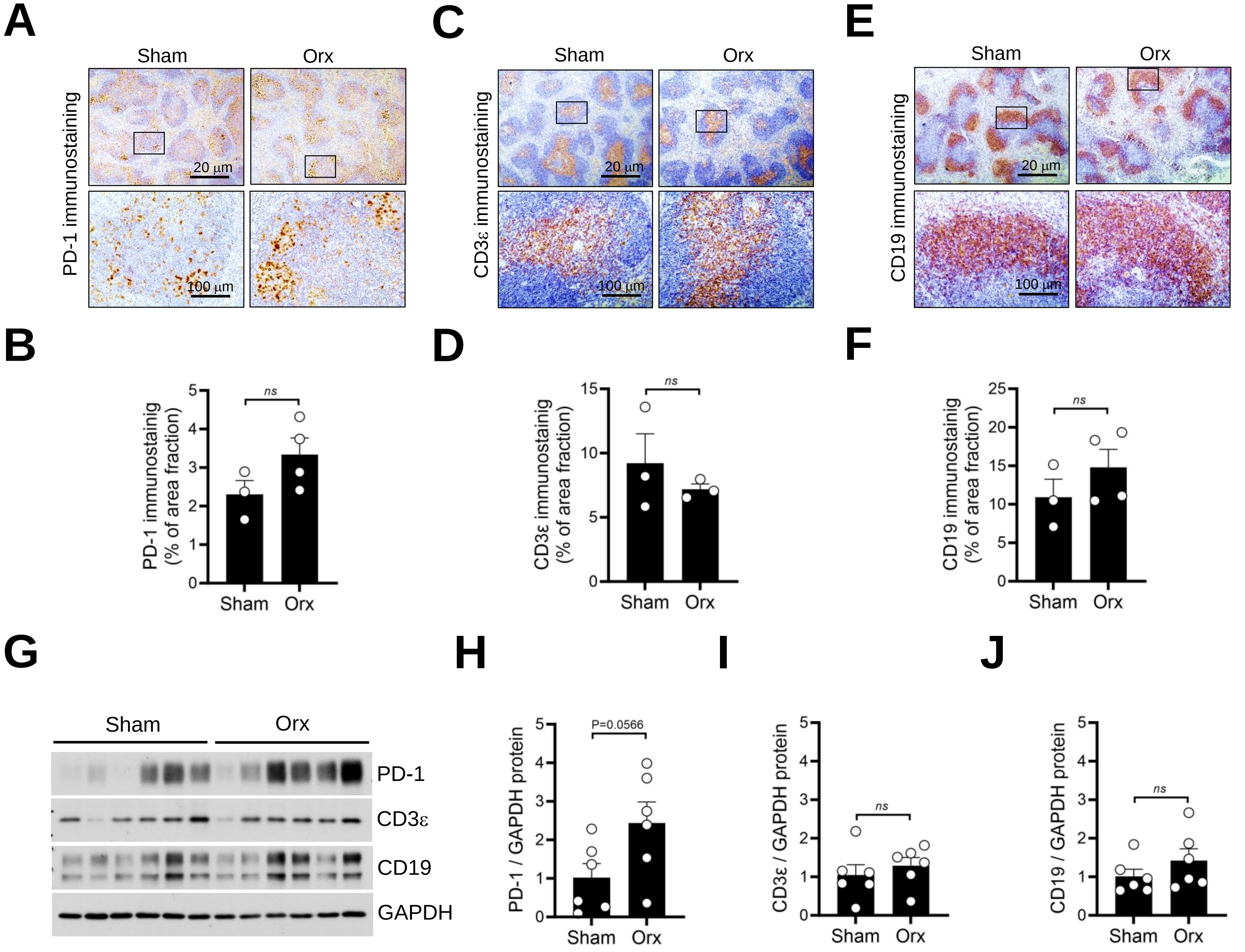

Supplemental Figure 23. Effects of gonadal androgen deprivation on PD-1, CD3, and CD19 protein levels in the spleen in mice without Aldo-salt administration. (**A−F**) Representative immunostainings and quantitative data of PD-1, CD3ε, and CD19 protein expression in the spleens from 10-month-old C57BL/6J mice with orx or sham operation but without Aldo-salt administration (n = 3-4/group). The percentage of areas fraction = (positive area / area of fields of view) x 100%. The data were calculated from five fields of view randomly photographed per splenic section per mouse. (**G−J**) Representative Western blots and quantitative data of PD-1, CD3ε, CD19, and GAPDH protein expression in the spleens from 10-month-old male mice with orx or sham operation but without Aldo-salt administration (n = 6/group). The data were expressed as mean ± SEM and analyzed by a two-tailed unpaired *t*-test. *ns, not significant.*

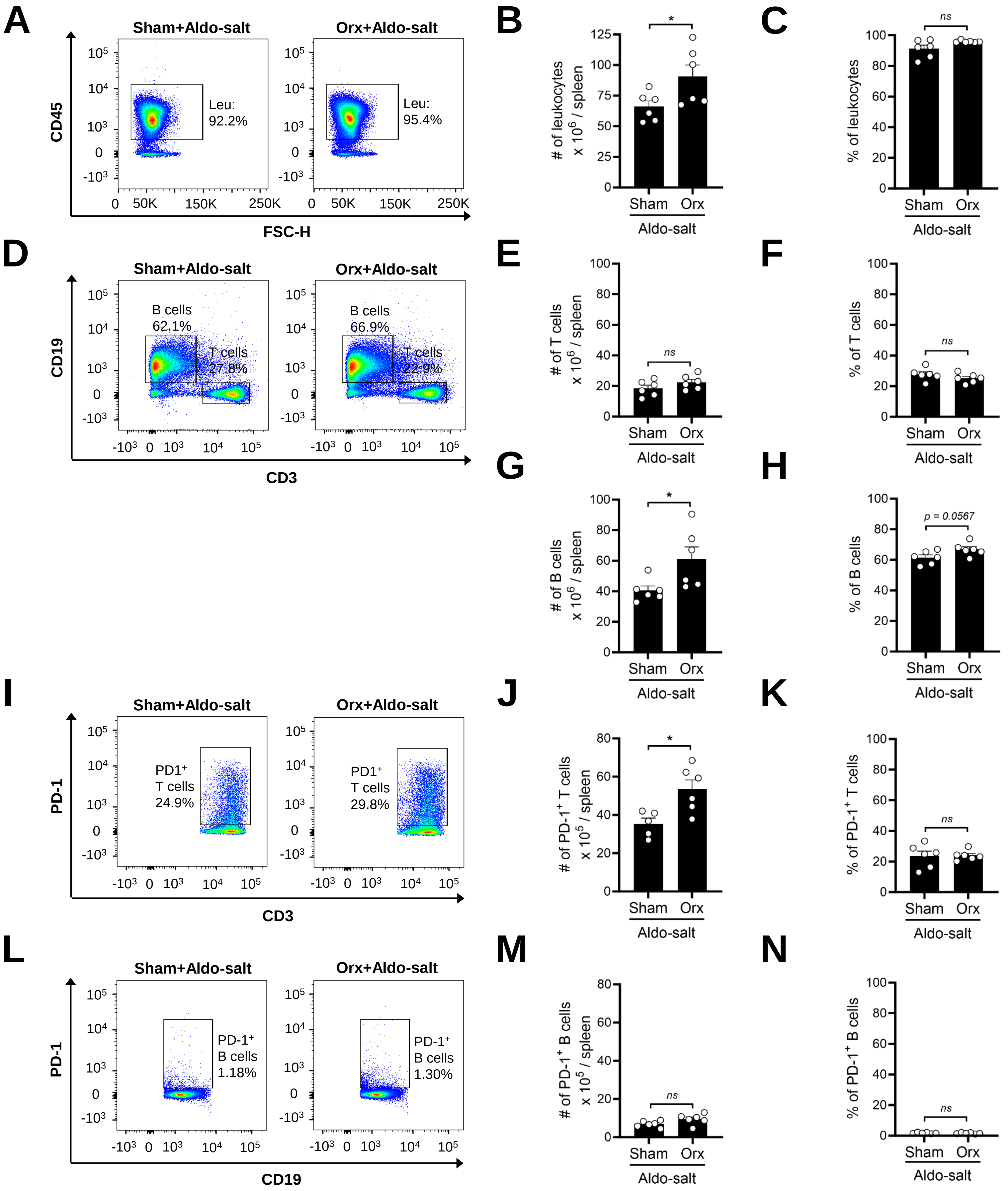

Supplemental Figure 24. Gonadal androgen deprivation increases the PD-1⁺ T cells but not PD-1⁺ B cells in the spleen in mice administered Aldo-salt. Representative pseudocolor plots and quantitative data of flow cytometry analysis of the total numbers (#) and percentage (%) of leukocytes (Leu; CD45⁺; % in total splenic cells; **A−C**), T cells (CD45⁺CD3⁺; % in total leu; **D**−F), B cells (CD45⁺CD19⁺; % in total leu; **D**, **G**, and **H**), PD-1⁺ T cells (CD45⁺CD3⁺PD-1⁺; % in total T cells; **I−K**), and PD-1⁺ B cells (CD45⁺CD19⁺PD-1⁺; % in total B cells; **L−N**) in the spleen from 10-month-old male C57BL/6J mice with orx or sham-operation ten days after Aldo-salt administration (n = 5-6/group). The data were expressed as mean ± SEM and analyzed by a two-tailed unpaired *t*-test. **, P < 0.05; ns, not significant.*

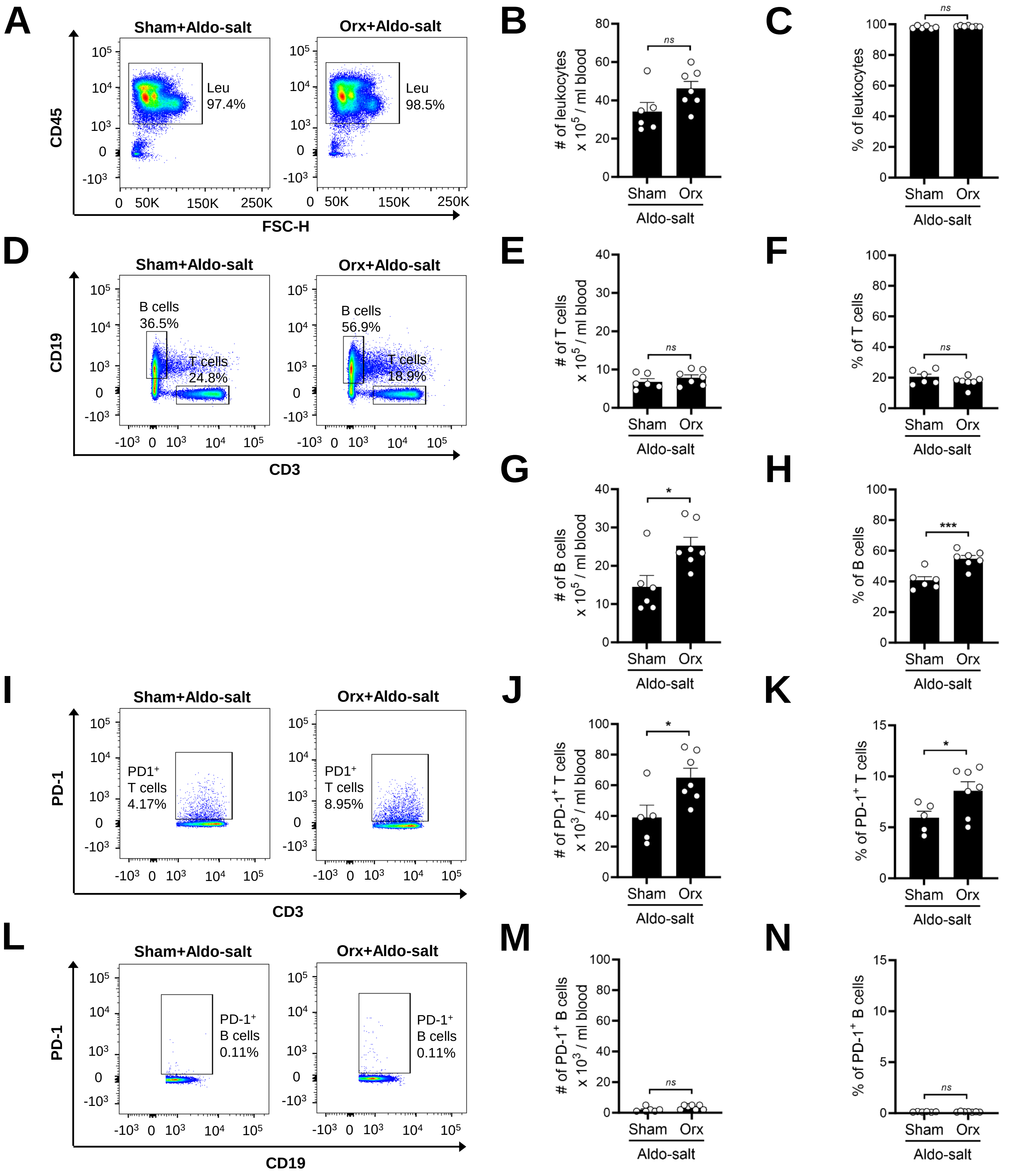

Supplemental Figure 25. Gonadal androgen deprivation augments PD-1$^+$ T cells but not PD-1$^+$ B cells in the blood in mice administered Aldo-salt. Representative pseudocolor plots and quantitative data of the flow cytometry analysis of the total numbers (#) and percentage (%) of leukocytes (Leu; CD45$^+$; % in total blood cells; **A–C**), T cells (CD45$^+$CD3$^+$; % in total leu; **D–**F), B cells (CD45$^+$CD19$^+$; % in total leu; **D**, **G**, and **H**), PD-1$^+$ T cells (CD45$^+$CD3$^+$PD-1$^+$; % in total T cells; **I–K**) and PD-1$^+$ B cells (CD45$^+$CD19$^+$PD-1$^+$; % in total B cells; **L–N**) in the blood from 10-month-old male C57BL/6J mice with orx or sham-operation ten days after Aldo-salt administration (n = 6-7/group). The data were expressed as mean ± SEM and analyzed by a two-tailed unpaired *t*-test. **, P < 0.05; ***, P < 0.001; ns, not significant.*

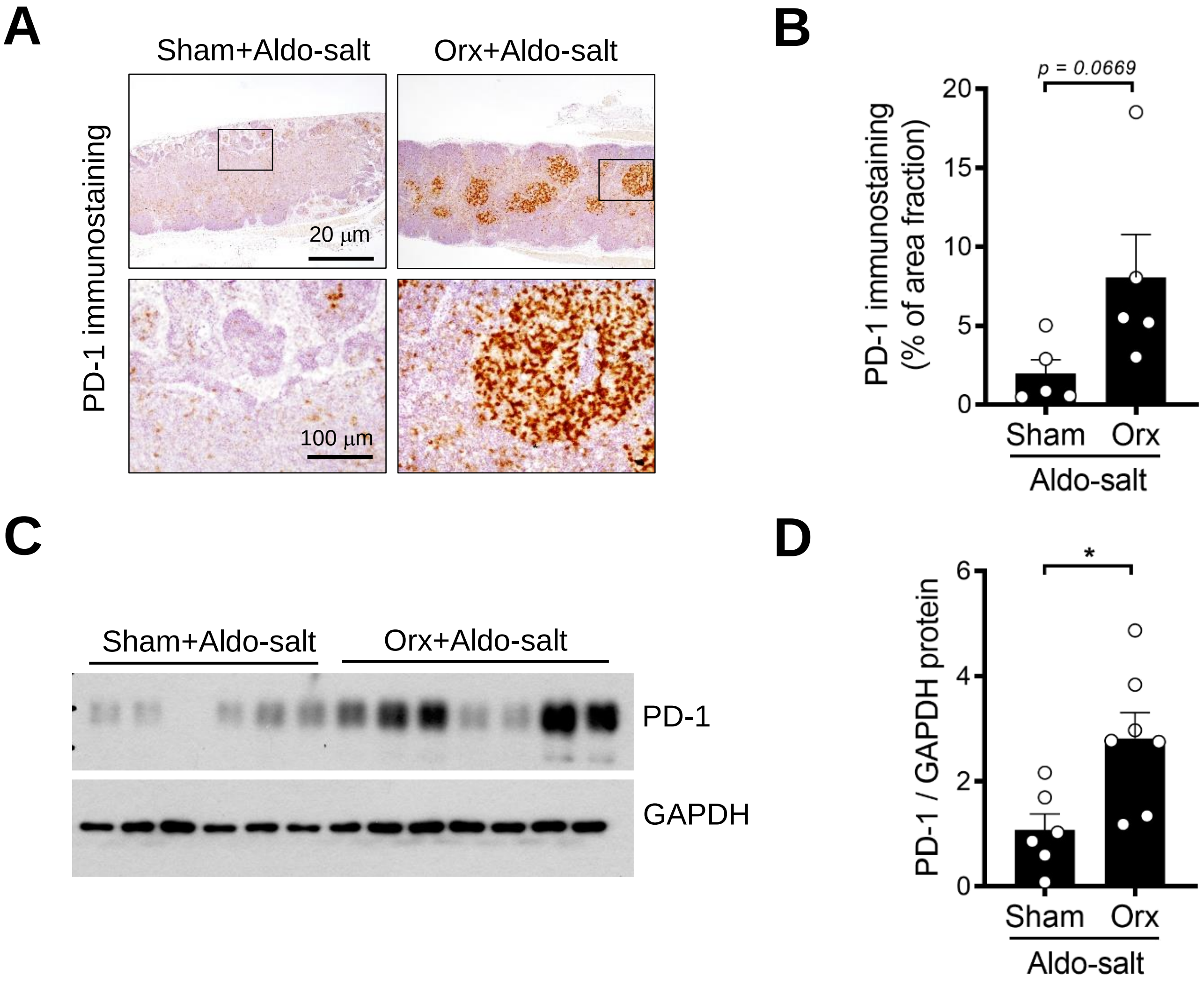

Supplemental Figure 26. Effects of gonadal androgen deprivation on PD-1 protein expression in the periaortic lymph nodes in mice administered with Aldo-salt. (A and **B)** Representative immunostaining (**A**) and quantitative data (**B**) of PD-1 protein expression in the periaortic lymph nodes from 10-month-old male C57BL/6J mice with orx and sham operation ten days after Aldo-salt administration (n = 5/group). (**C** and **D**) Representative Western blots (**C**) and quantitative data (**D**) of PD-1 protein expression in the periaortic lymph nodes from 10-month-old male C57BL/6J mice with orx and sham-operated ten days after Aldo-salt administration (n = 6-7/group). PD-1 protein expressions were normalized to GAPDH. The data were expressed as mean ± SEM and analyzed by a two-tailed unpaired *t*-test. *, $P < 0.05$.

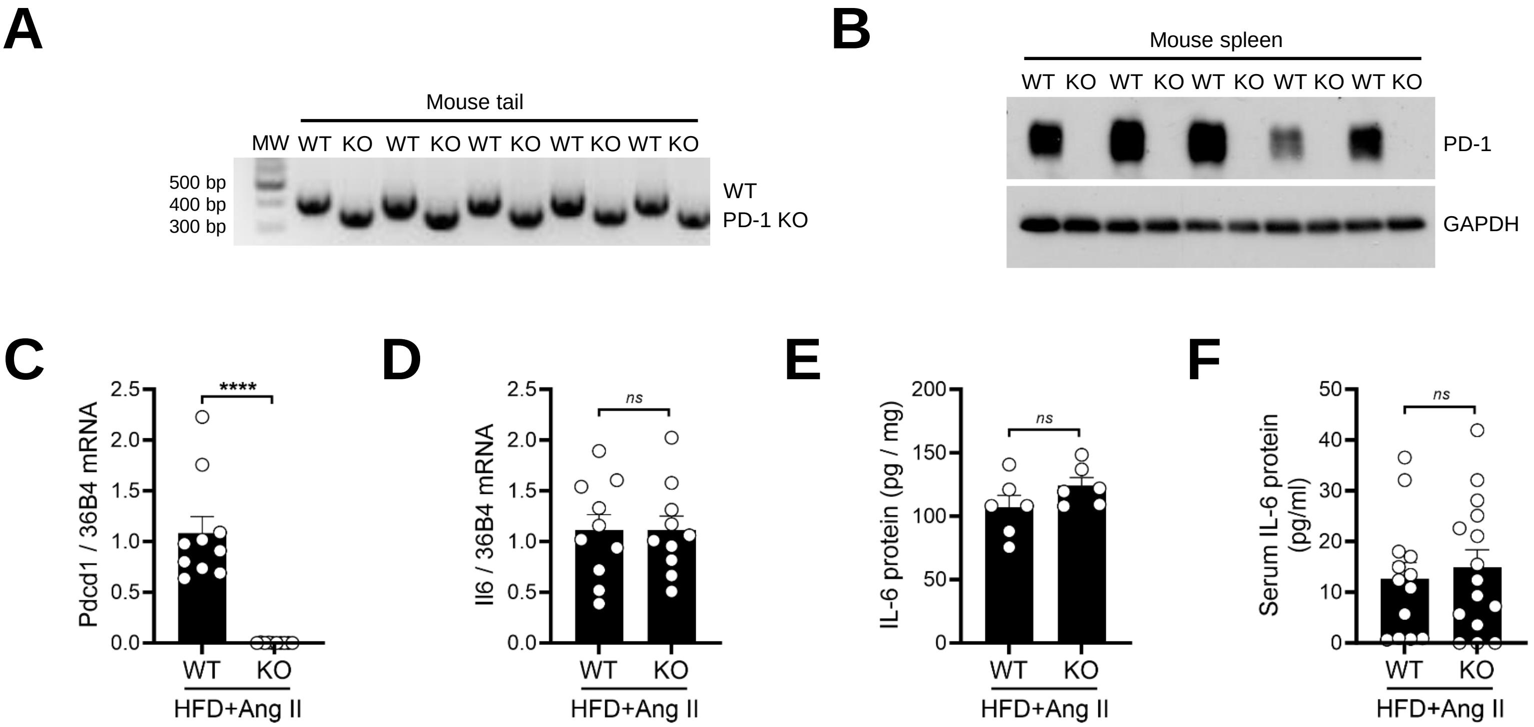

Supplemental Figure 27. Genetic deletion of PD-1 did not affect IL-6 mRNA and protein expressions in the spleen and serum in mice administered high-fat diet and Ang II. (**A** and **B**) PCR genotyping (**A**) and Western blots of PD-1 protein expression in the spleens (**B**) in PD-1 KO and WT male C57BL/6J mice. (**C** and **D**) PD-1 (Pdcd1; **C**) and IL-6 (Il6; **D**) mRNA expressions were determined by real-time PCR in the spleen in 4-month-old male PD-1 KO and WT male mice with 8-week high-fat diet (HFD) feeding and 4-week Ang II infusion (HFD+Ang II; n = 10/group). (**E** and **F**) IL-6 protein expression was determined by ELISA in the spleen (**E**; n = 6/group) and serum (**F**; n = 11-12/group) of the same 4-month-old male PD-1 KO and WT mice with HFD+Ang II administration. The data were expressed as mean ± SEM and analyzed by a two-tailed unpaired t-test. ****, *P < 0.0001. ns, not significant.*

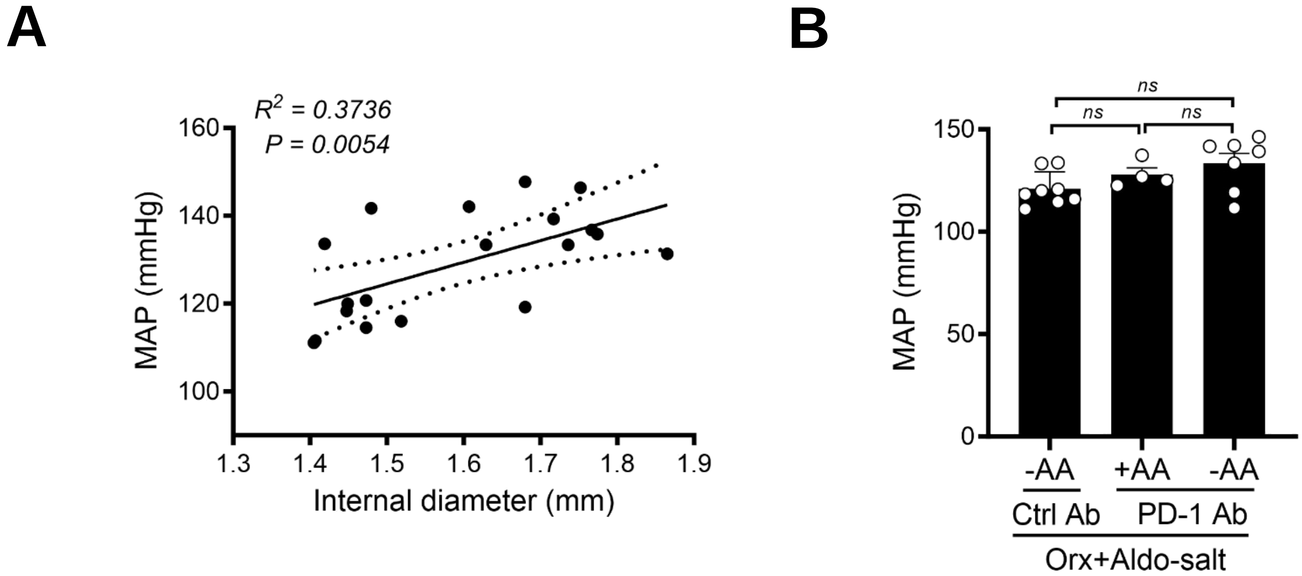

Supplemental Figure 28. Blood pressure, internal diameters of the aortic arch, and aortic aneurysms in orchiectomized mice administered Aldo-salt and anti-PD-1 or control antibodies. (**A**) Correlation analysis of the internal diameter of the ArchAo and MAP in orchiectomized 10-month-old male C57BL/6J mice seven weeks after Aldo-salt and anti-PD-1 or control Ab administration (n = 19/group). (**B**) MAP in orchiectomized 10-month-old male C57BL/6J mice administered Aldo-salt with anti-PD-1 or control Ab and with (+) or without (-) aortic aneurysms (n = 4-8/group). The data were expressed as mean ± SEM and analyzed by simple linear regression analysis (**A**) and one-way ANOVA for multiple comparison tests (**B**). *ns, not statistically significant.*

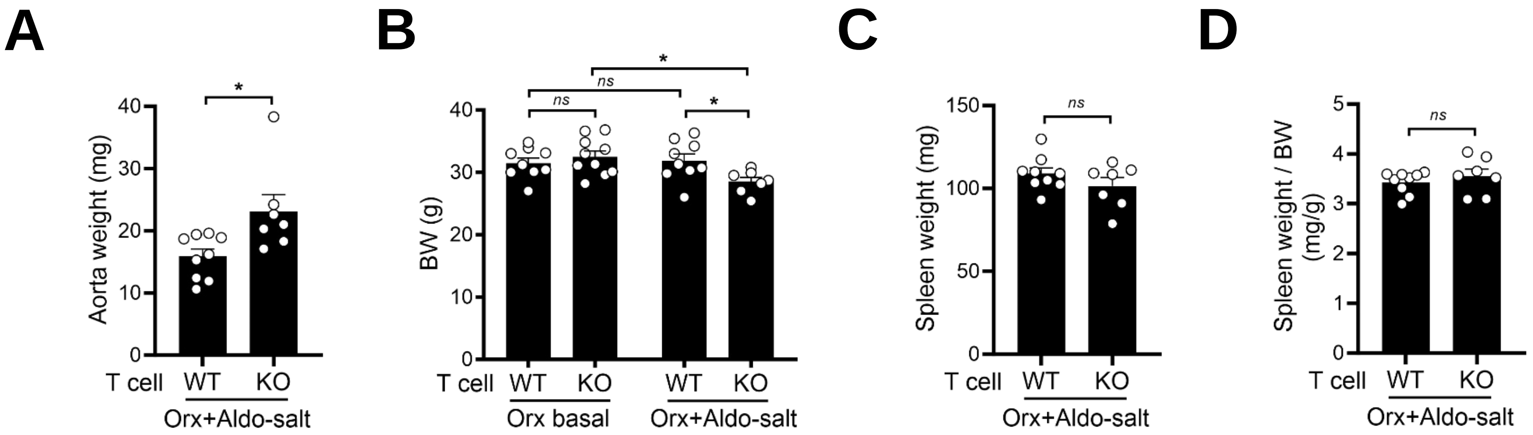

Supplemental Figure 29. The effect of adoptive PD-1 deficient T cell transfer on aorta weight, body weight, and spleen weight in orchiectomized mice administered Aldo-salt. Aorta weight (**A**; n = 7-9/group), BW (**B**; n = 7-10/group), spleen weight (**C**; n = 7-9/group), and the ratio of spleen weight to BW (**D**; n = 7-9/group) in 9-10-month-old orchiectomized C57BL/6J male mice four weeks after Aldo-salt with adoptive PD-1 KO and WT T cell transfer via retro-orbital sinus injection two days before and eight and eighteen days after Aldo- salt administration. PD-1 KO and WT T cells were isolated from 4-5-month-old male PD-1 KO and WT C57BL6J mice. The data were expressed as mean ± SEM and analyzed by unpaired two-tailed *t*-test (**A**, **C**, and **D**) and two-way ANOVA with multiple comparison tests (**B**) and **, p<0.05; ****, p<0.0001; ns, not significant.*

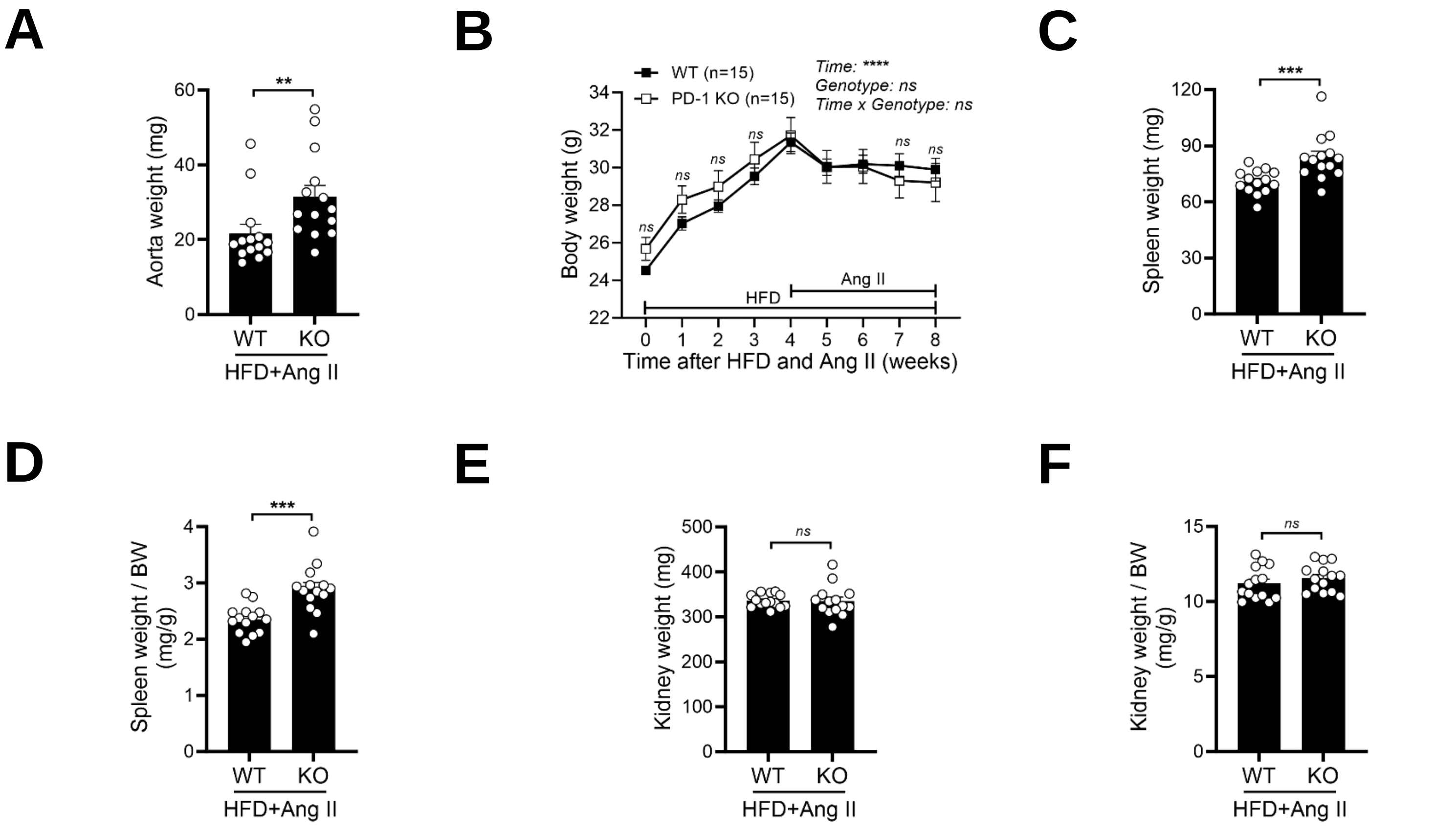

Supplemental Figure 30. The effects of genetic deletion of PD-1 on aorta weight, body weight, spleen weight, and kidney weight in mice administered high-fat diet and angiotensin II. The aorta weight (**A**; n = 14/group), BW (**B**; n = 15/group), spleen weight (**C;** n = 14/group), the ratio of spleen weight to BW (**D**; n = 14/group), kidney weight (**E;** n = 14/group), and ratio of kidney weight to BW (**F**; n = 14/group) in 4-month-old male PD-1 knockout (PD-1 KO) and wild-type (WT) C57BL/6J mice with 8-week HFD feeding 4-week Ang II infusion. The data were expressed as mean ± SEM and analyzed by unpaired two-tailed *t*-test (**A** and **C−F**) and two-way ANOVA with multiple comparison tests (**B**) and ***, *p<0.001; ****, p<0.0001; ns, not significant.*

Supplemental Table 1. A complete list of 180 genes whose mRNAs are upregulated by orchiectomy (Orx *vs. S*ham Ctrl) but downregulated by exogenous dihydrotestosterone (DHT) administration to orchiectomized mice (Orx+DHT *vs.* Orx) in the aorta in mice administered Aldo-salt

#	Gene ID	Description	Location	Fold Change (Orx *vs.* Ctrl)	*P*-value	Fold Change (Orx+DHT *vs.* Orx)	*P*-value
1	Krt17	keratin 17	chr11	72.56	0.00293234	-93.23	0.00515206
2	Alb	albumin	chr5	66.33	0.00000639	-11.24	0.00140744
3	Tbata	thymus, brain and testes associated	chr10	53.87	0.00035045	-28.29	0.00098214
4	Hamp2	hepcidin antimicrobial peptide 2	chr7	39.67	0.00003697	-23.43	0.00045217
5	Camp	cathelicidin antimicrobial peptide	chr9	23.37	0.00009690	-4.54	0.00397801
6	Ngp	neutrophilic granule protein	chr9	20.00	0.00000009	-6.09	0.00006854
7	S100a8	S100 calcium binding protein A8 (calgranulin A)	chr3	13.36	0.00000001	-3.40	0.00378026
8	H2-Eb2	histocompatibility 2, class II antigen E beta2	chr17	12.48	0.00020575	-10.62	0.00301283
9	Themis	thymocyte selection associated	chr10	12.16	0.00429165	-18.45	0.00022233
10	S100a9	S100 calcium binding protein A9 (calgranulin B)	chr3	11.99	0.00000000	-3.20	0.00339268
11	Myb	myeloblastosis oncogene	chr10	10.97	0.00009978	-17.17	0.00000386
12	Cr2	complement receptor 2	chr1	10.59	0.00073704	-14.30	0.00047904
13	Cd79a	CD79A antigen	chr7	10.49	0.00000089	-8.41	0.00005525
14	Ms4a1	membrane-spanning 4-domains A1	chr19	10.47	0.00000850	-18.94	0.00000015
15	Cd19	CD19 antigen	chr7	10.22	0.00000678	-10.85	0.00001361
16	Sh2d1a	SH2 domain containing 1A	chrX	9.42	0.00433350	-17.30	0.00136215
17	Tnfrsf13c	TNF receptor superfamily member 13c	chr15	9.40	0.00022008	-8.35	0.00058788
18	Mzb1	marginal zone B and B1 cell-specific protein 1	chr18	9.10	0.00007934	-10.04	0.00012146
19	Fcmr	Fc fragment of IgM receptor	chr1	9.06	0.00055230	-9.07	0.00033054
20	Prg2	proteoglycan 2, bone marrow	chr2	8.97	0.00009111	-8.38	0.00008549
21	Cd8b1	CD8 antigen, beta chain 1	chr6	8.79	0.00000930	-11.39	0.00000187
22	Bcl11a	B cell CLL/lymphoma 11A	chr11	8.78	0.00011468	-12.34	0.00004661
23	Gfi1	growth factor independent 1 transcription repressor	chr5	8.54	0.00072452	-4.90	0.00809827
24	Sidt1	SID1 transmembrane family member 1	chr16	8.49	0.00140464	-11.35	0.00184782
25	Cacna1e	calcium voltage-gated channel subunit alpha1 E	chr1	8.38	0.00018835	-5.63	0.00415678
26	Cxcr5	chemokine (C-X-C motif) receptor 5	chr9	8.36	0.00008848	-10.71	0.00009679
27	Cd79b	CD79B antigen	chr11	8.28	0.00000775	-7.62	0.00002434
28	Spib	Spi-B transcription factor	chr7	8.21	0.00012726	-17.88	0.00000381
29	Cd8a	CD8 antigen, alpha chain	chr6	8.18	0.00007024	-14.10	0.00000312
30	Gimap7	GTPase, IMAP family member 7	chr6	7.92	0.00242371	-6.81	0.00635858
31	Pax5	paired box 5	chr4	7.79	0.00042815	-7.91	0.00004445
32	Siglecg	sialic acid binding Ig-like lectin G	chr7	7.76	0.00059477	-9.95	0.00010210
33	Ly6d	lymphocyte antigen 6 complex, locus D	chr15	7.74	0.00002771	-8.43	0.00001140
34	Cd22	CD22 antigen	chr7	7.66	0.00036876	-7.85	0.00021901
35	Txk	TXK tyrosine kinase	chr5	7.58	0.00406763	-15.63	0.00131832
36	Fcrla	Fc receptor-like A	chr1	7.31	0.00058840	-9.12	0.00026021
37	Pou2af1	POU class 2 homeobox associating factor 1	chr9	6.47	0.00070169	-9.62	0.00001364
38	Ltb	lymphotoxin B	chr17	6.32	0.00005346	-5.68	0.00022554
39	Fcrl1	Fc receptor-like 1	chr3	6.30	0.00640415	-8.28	0.00467074
40	Ms4a4b	membrane-spanning 4-domains, subfamily A, 4B	chr19	6.25	0.00054053	-8.63	0.00030896
41	Bcl11b	B cell leukemia/lymphoma 11B	chr12	6.20	0.00022008	-17.37	0.00000158
42	H2-Oa	histocompatibility 2, O region alpha locus	chr17	6.18	0.00530697	-15.90	0.00057125
43	Ly6c2	lymphocyte antigen 6 complex, locus C2	chr15	6.00	0.00048563	-5.97	0.00008967
44	Slamf6	SLAM family member 6	chr1	5.99	0.00844321	-7.29	0.00744750
45	Cd27	CD27 antigen	chr6	5.94	0.00088574	-6.77	0.00219890
46	Cd5	CD5 antigen	chr19	5.83	0.00000845	-2.99	0.00188554
47	Gjb2	gap junction protein, beta 2	chr14	5.62	0.00822289	-8.75	0.00898610
48	H2-Ob	histocompatibility 2, O region beta locus	chr17	5.52	0.00051984	-5.94	0.00053131
49	Lef1	lymphoid enhancer binding factor 1	chr3	5.47	0.00024262	-5.34	0.00027613
50	Ptprcap	protein tyrosine phosphatase receptor type C associated protein	chr19	5.44	0.00016530	-7.24	0.00002414
51	H2-DMb2	histocompatibility 2, class II, locus Mb2	chr17	5.36	0.00006367	-6.08	0.00003221
52	Cd69	CD69 antigen	chr6	5.32	0.00309230	-4.77	0.00370211
53	Cd3g	CD3 antigen, gamma polypeptide	chr9	5.30	0.00000798	-4.02	0.00040940
54	Bcl2a1d	B cell leukemia/lymphoma 2 related protein A1d	chr9	5.25	0.00053813	-8.40	0.00013498
55	Sell	selectin, lymphocyte	chr1	5.20	0.00121179	-6.00	0.00015825
56	Lck	lymphocyte protein tyrosine kinase	chr1	5.11	0.00053414	-6.87	0.00003226
57	Tcf7	transcription factor 7, T cell specific	chr11	4.90	0.00015294	-8.26	0.00000127
58	Treml4	triggering receptor expressed on myeloid cells-like 4	chr17	4.84	0.00605111	-4.78	0.00891611
59	Card11	caspase recruitment domain family, member 11	chr5	4.77	0.00022641	-9.47	0.00001543
60	Itk	IL2 inducible T cell kinase	chr11	4.66	0.00077488	-10.58	0.00000563
61	Azgp1	alpha-2-glycoprotein 1, zinc	chr5	4.58	0.00226445	-13.71	0.00006137
62	Traf3ip3	TRAF3 interacting protein 3	chr1	4.57	0.00122313	-9.23	0.00001125
63	Col11a1	collagen, type XI, alpha 1	chr3	4.53	0.00386393	-4.11	0.00926047
64	Prss35	protease, serine 35	chr9	4.47	0.00552033	-4.64	0.00871472
65	Il18r1	interleukin 18 receptor 1	chr1	4.47	0.00633410	-13.41	0.00002101
66	Btla	B and T lymphocyte associated	chr16	4.46	0.00219236	-5.82	0.00069288
67	Ikzf3	IKAROS family zinc finger 3	chr11	4.42	0.00042893	-11.40	0.00000050
68	Cd3d	CD3 antigen, delta polypeptide	chr9	4.34	0.00091830	-5.79	0.00041619
69	Cytip	cytohesin 1 interacting protein	chr2	4.27	0.00125618	-6.67	0.00003467
70	Blk	B lymphoid kinase	vhr14	4.27	0.00543365	-5.22	0.00050444

Supplemental Table 1 (continued). A complete list of 180 genes whose mRNAs are upregulated by orchiectomy (Orx *vs. S*ham Ctrl) but downregulated by exogenous dihydrotestosterone (DHT) administration to orchiectomized mice (Orx+DHT *vs.* Orx) in the aorta in mice administered Aldo-salt

#	Gene ID	Description	Location	Fold Change (Orx *vs.* Ctrl)	*P*-value	Fold Change (Orx+DHT *vs.* Orx)	*P*-value
71	Rnase6	ribonuclease, RNase A family, 6	chr14	4.25	0.00062204	-3.62	0.00697734
72	Rasal3	RAS protein activator like 3	chr17	4.18	0.00026567	-5.66	0.00009229
73	Tcf21	transcription factor 21	chr10	4.15	0.00359346	-6.98	0.00028077
74	Cd28	CD28 antigen	chr1	4.10	0.00487485	-11.30	0.00015504
75	Acap1	ArfGAP with coiled-coil, ankyrin repeat and PH domains 1	Chr11	4.04	0.00049392	-3.99	0.00106126
76	Itgax	integrin alpha X	Chr7	3.91	0.00021341	-7.78	0.00000025
77	Fcho1	FCH domain only 1	chr8	3.77	0.00028312	-5.48	0.00004304
78	Tbc1d10c	TBC1 domain family, member 10c	chr19	3.72	0.00335201	-6.03	0.00014369
79	Ipcef1	interaction protein for cytohesin exchange factors 1	chr10	3.72	0.00063026	-4.62	0.00033393
80	Cdca7	cell division cycle associated 7	chr2	3.64	0.00219618	-4.19	0.00117219
81	Rhbg	Rhesus blood group-associated B glycoprotein	chr3	3.61	0.00461591	-3.67	0.00831695
82	Grap2	GRB2-related adaptor protein 2	chr15	3.59	0.00042164	-4.61	0.00008908
83	P2ry10	purinergic receptor P2Y, G-protein coupled 10	chrX	3.59	0.00518994	-8.35	0.00006476
84	Cd3e	CD3 antigen, epsilon polypeptide	chr9	3.57	0.00107340	-5.39	0.00080476
85	Map4k1	mitogen-activated protein kinase kinase kinase kinase 1	chr7	3.46	0.00065314	-3.55	0.00033196
86	Gimap3	GTPase, IMAP family member 3	chr6	3.46	0.00052659	-3.27	0.00106921
87	Itgal	integrin alpha L	chr7	3.39	0.00065878	-5.42	0.00000829
88	Parvg	parvin, gamma	chr15	3.30	0.00275671	-3.46	0.00086344
89	Rasgrp1	RAS guanyl releasing protein 1	chr2	3.26	0.00013955	-6.18	0.00000003
90	Lat	linker for activation of T cells	chr7	3.25	0.00028462	-4.26	0.00003633
91	Ptpn22	protein tyrosine phosphatase, non-receptor type 22	chr3	3.25	0.00017971	-5.64	0.00000163
92	Gpr141b	G protein-coupled receptor 141B	chr13	3.25	0.00714189	-4.58	0.00272678
93	Cd2	CD2 antigen	chr3	3.21	0.00547508	-3.89	0.00071948
94	Skap1	src family associated phosphoprotein 1	chr11	3.15	0.00086272	-5.52	0.00005163
95	Unc13d	unc-13 homolog D	chr11	3.12	0.00115720	-2.97	0.00141585
96	Cd52	CD52 antigen	chr4	3.06	0.00053838	-3.10	0.00054816
97	Il7r	interleukin 7 receptor	chr15	3.03	0.00465978	-5.88	0.00004590
98	Cxcr6	chemokine (C-X-C motif) receptor 6	chr9	3.00	0.00662081	-7.99	0.00000974
99	Sla	src-like adaptor	chr15	3.00	0.00031091	-4.40	0.00000104
100	Itgb7	integrin beta 7	chr15	2.90	0.00292785	-4.49	0.00001454
101	Stat4	signal transducer and activator of transcription 4	chr1	2.86	0.00813945	-7.63	0.00002618
102	Bcl2a1b	B cell leukemia/lymphoma 2 related protein A1b	chr9	2.84	0.00505359	-4.53	0.00043336
103	Gbp8	guanylate-binding protein 8	chr5	2.84	0.00241229	-6.61	0.00000543
104	Nup210	nucleoporin 210	chr6	2.80	0.00257918	-2.49	0.00690455
105	Cd37	CD37 antigen	chr7	2.80	0.00119792	-3.14	0.00034960
106	Rhoh	ras homolog family member H	chr5	2.78	0.00255259	-3.77	0.00029389
107	Pmaip1	phorbol-12-myristate-13-acetate-induced protein 1	chr18	2.77	0.00940216	-4.14	0.00081350
108	Il2rb	interleukin 2 receptor, beta chain	chr15	2.56	0.00166477	-3.37	0.00009137
109	Wdfy4	WD repeat and FYVE domain containing 4	chr14	2.54	0.00147824	-4.00	0.00000055
110	Il21r	interleukin 21 receptor	chr7	2.52	0.00218437	-3.53	0.00000894
111	Cyp4f18	cytochrome P450, family 4, subfamily f, polypeptide 18	chr8	2.52	0.00593724	-3.69	0.00057901
112	Ezr	ezrin	chr17	2.52	0.00921918	-4.72	0.00001711
113	Coro1a	coronin, actin binding protein 1A	chr7	2.49	0.00009215	-2.88	0.00001024
114	Cd247	CD247 antigen	chr1	2.49	0.00011772	-3.17	0.00006881
115	Mamdc2	MAM domain containing 2	chr19	2.46	0.00309219	-2.72	0.00103849
116	Blnk	B cell linker	chr19	2.43	0.00651873	-3.26	0.00042273
117	Arl5c	ADP-ribosylation factor-like 5C	chr11	2.42	0.00540941	-2.40	0.00739382
118	Sash3	SAM and SH3 domain containing 3	chrX	2.38	0.00115611	-3.04	0.00005892
119	Rgs14	regulator of G-protein signaling 14	chr13	2.38	0.00720522	-4.80	0.00003052
120	Rac2	Rac family small GTPase 2	chr15	2.31	0.00109290	-2.70	0.00034669
121	Irf8	interferon regulatory factor 8	chr8	2.31	0.00992903	-3.21	0.00020599
122	H2-DMa	histocompatibility 2, class II, locus DMa	chr17	2.30	0.00256607	-2.50	0.00221721
123	Ptprc	protein tyrosine phosphatase receptor type C	chr1	2.29	0.00466891	-4.49	0.00000005
124	H2-Ab1	histocompatibility 2, class II antigen A, beta 1	chr17	2.28	0.00279696	-2.65	0.00038114
125	Shisa2	shisa family member 2	chr14	2.28	0.00544805	-2.51	0.00417194
126	Cd74	CD74 antigen	chr18	2.24	0.00281218	-2.53	0.00042047
127	Hvcn1	hydrogen voltage-gated channel 1	chr5	2.24	0.00437503	-2.28	0.00306926
128	Ikzf1	IKAROS family zinc finger 1	chr11	2.22	0.00263754	-3.87	0.00000016
129	Psd4	pleckstrin and Sec7 domain containing 4	chr2	2.18	0.00345950	-2.98	0.00019628
130	Spn	sialophorin	chr7	2.14	0.00363685	-3.06	0.00000287
131	Hmgb2	high mobility group box 2	chr8	2.14	0.00003441	-2.26	0.00000462
132	Selplg	selectin, platelet (p-selectin) ligand	chr5	2.13	0.00314154	-2.85	0.00012451
133	Dlgap5	DLG associated protein 5	chr14	2.12	0.00762526	-4.69	0.00001236
134	H2-Aa	histocompatibility 2, class II antigen A, alpha	chr17	2.09	0.00643965	-2.23	0.00426046
135	Sema4d	semaphorin 4D	chr13	2.07	0.00119369	-2.21	0.00083843
136	Mpeg1	macrophage expressed gene 1	chr19	2.07	0.00739749	-2.95	0.00001279
137	Myo1g	myosin IG	chr11	2.05	0.00819989	-3.85	0.00000032
138	Pclaf	PCNA clamp associated factor	chr9	2.00	0.00922412	-3.65	0.00000918
139	Ptpn6	protein tyrosine phosphatase, non-receptor type 6	chr6	2.00	0.00882316	-2.98	0.00010761
140	Kif11	kinesin family member 11	chr19	1.97	0.00320371	-4.96	0.00000001

Supplemental Table 1 (continued). A complete list of 180 genes whose mRNAs are upregulated by orchiectomy (Orx *vs. S*ham Ctrl) but downregulated by exogenous dihydrotestosterone (DHT) administration to orchiectomized mice (Orx+DHT *vs.* Orx) in the aorta in mice administered Aldo-salt

#	Gene ID	Description	Location	Fold Change (Orx *vs.* Ctrl)	*P*-value	Fold Change (Orx+DHT *vs.* Orx)	*P*-value
141	St3gal6	ST3 beta-galactoside alpha-2,3-sialyltransferase 6	chr16	1.95	0.00015243	-2.99	0.00000000
142	Slc7a10	solute carrier family 7 member 10	chr7	1.91	0.00003684	-2.91	0.00000657
143	Endou	endonuclease, polyU-specific	chr15	1.90	0.00958322	-2.30	0.00131035
144	Top2a	topoisomerase (DNA) II alpha	chr11	1.88	0.00650663	-3.78	0.00000001
145	Mthfd1l	methylenetetrahydrofolate dehydrogenase 1-like	chr10	1.85	0.00002071	-1.48	0.00306873
146	Arhgap45	Rho GTPase activating protein 45	chr10	1.83	0.00825779	-2.20	0.00039083
147	Nusap1	nucleolar and spindle associated protein 1	chr2	1.83	0.00103524	-2.44	0.00004059
148	Ccr2	chemokine (C-C motif) receptor 2	chr9	1.81	0.00949725	-3.16	0.00000078
149	Tbx20	T-box 20	chr9	1.81	0.00592786	-2.08	0.00196389
150	Lat2	linker for activation of T cells family, member 2	chr5	1.79	0.00805564	-2.53	0.00004088
151	Bmp3	bone morphogenetic protein 3	chr5	1.79	0.00004372	-1.45	0.00473755
152	Rrm2	ribonucleotide reductase M2	chr12	1.77	0.00727620	-2.80	0.00006412
153	Galnt6	polypeptide N-acetylgalactosaminyltransferase 6	chr15	1.76	0.00672652	-2.33	0.00018578
154	Alcam	activated leukocyte cell adhesion molecule	chr16	1.72	0.00193548	-1.84	0.00039358
155	Cdca8	cell division cycle associated 8	chr4	1.71	0.00379456	-2.31	0.00075954
156	Cpxm1	carboxypeptidase X 1 (M14 family)	vhr2	1.68	0.00002026	-2.04	0.00001212
157	Irf5	interferon regulatory factor 5	chr6	1.65	0.00422174	-2.51	0.00000055
158	Cpz	carboxypeptidase Z	chr5	1.62	0.00211843	-2.33	0.00009330
159	Kifc1	kinesin family member C1	chr17	1.61	0.00700034	-2.47	0.00008529
160	Sox4	SRY (sex determining region Y)-box 4	chr13	1.58	0.00208812	-1.74	0.00020357
161	Gpc3	glypican 3	chrX	1.52	0.00073456	-3.09	0.00000000
162	Arl4c	ADP-ribosylation factor-like 4C	chr1	1.50	0.00835630	-1.84	0.00005559
163	Ctsk	cathepsin K	chr3	1.49	0.00252571	-1.84	0.00014613
164	St3gal4	ST3 beta-galactoside alpha-2,3-sialyltransferase 4	chr9	1.48	0.00249456	-1.57	0.00090671
165	Efemp1	EGF-containing fibulin-like extracellular matrix protein 1	chr11	1.48	0.00960942	-2.46	0.00000004
166	Chst2	carbohydrate sulfotransferase 2	chr9	1.43	0.00706828	-1.56	0.00174383
167	Mcm4	minichromosome maintenance complex component 4	chr16	1.42	0.00303205	-1.45	0.00020502
168	Mcm3	minichromosome maintenance complex component 3	chr1	1.40	0.00005370	-1.63	0.00000000
169	Mmp2	matrix metallopeptidase 2	chr19	1.35	0.00322527	-1.90	0.00000004
170	Prcp	prolylcarboxypeptidase (angiotensinase C)	chr7	1.33	0.00586741	-1.58	0.00000028
171	Sparcl1	SPARC-like 1	chr5	1.31	0.00162648	-1.39	0.00000000
172	Rcn1	reticulocalbin 1	chr2	1.31	0.00273570	-1.57	0.00011684
173	Cmtm3	CKLF-like MARVEL transmembrane domain containing 3	chr8	1.29	0.00967474	-1.47	0.00487152
174	C1ra	complement component 1, r subcomponent A	chr6	1.28	0.00999725	-1.71	0.00000593
175	Pdcd4	programmed cell death 4	chr19	1.26	0.00654881	-1.40	0.00017664
176	Pcna	proliferating cell nuclear antigen	chr2	1.24	0.00890499	-1.42	0.00001990
177	Hmgb1	high mobility group box 1	chr5	1.24	0.00172916	-1.30	0.00004352
178	Pon2	paraoxonase 2	chr6	1.23	0.00892161	-1.34	0.00006575
179	Alyref	Aly/REF export factor	chr11	1.23	0.00625671	-1.32	0.00066921
180	Aqp1	aquaporin 1	chr6	1.17	0.00964582	-1.34	0.00000000

Supplemental Table 2. A complete list of 150 genes whose mRNAs are downregulated by orchiectomy (Orx *vs.* Sham Ctrl) but upregulated by exogenous dihydrotestosterone (DHT) administration to orchiectomized mice (Orx+DHT *vs.* Orx) in the aorta in mice administered Aldo-salt

#	Gene ID	Description	Location	Fold Change (Orx *vs.* Ctrl)	*P*-value	Fold Change (Orx+DHT *vs.* Orx)	*P*-value
1	Myh4	myosin heavy chain 4, skeletal muscle	chr11	-26.73655093	0.00006572	13.92182629	0.00655175
2	Cacng6	calcium channel, voltage-dependent, gamma subunit 6	chr7	-24.8409316	0.00276263	34.25279623	0.00031960
3	Slco1a1	solute carrier organic anion transporter family, member 1a1	chr6	-17.09886947	0.00233895	19.12046232	0.00069800
4	Scd3	stearoyl-coenzyme A desaturase 3	chr19	-15.297534	0.00034029	10.74260432	0.00185519
5	Gm45915	immune system associated SCR family kinase partner	chr14	-12.55500926	0.00721071	18.11818619	0.00484563
6	Atp2a1	ATPase, Ca++ transporting, cardiac muscle, fast twitch 1	chr7	-9.529129404	0.00221234	11.66179158	0.00002494
7	Bmp8b	bone morphogenetic protein 8b	chr4	-8.942783046	0.00154569	6.639997161	0.00049010
8	Pvalb	parvalbumin	chr15	-8.822837329	0.00479196	6.289348289	0.00563687
9	Hephl1	hephaestin-like 1	chr9	-8.50672212	0.00000000	7.173434607	0.00000007
10	Mybpc1	myosin binding protein C, slow-type	chr10	-7.475004077	0.00826108	24.30187667	0.00000293
11	Rspo4	R-spondin 4	chr2	-5.295639174	0.00762548	6.232415488	0.00003637
12	Tnnc2	troponin C2, fast	chr2	-5.279964009	0.00430892	8.774819367	0.00000080
13	Actn3	actinin alpha 3	chr19	-5.149314201	0.00176955	4.103126291	0.00081762
14	Mylpf	myosin light chain, phosphorylatable, fast skeletal muscle	chr7	-5.030485019	0.00066253	7.205542241	0.00000006
15	Adcy10	adenylate cyclase 10	chr1	-4.824251965	0.00009234	3.767798349	0.00021866
16	6430571L13Rik	RIKEN cDNA 6430571L13 gene	chr9	-4.779242313	0.00000351	3.82626693	0.00000183
17	Slc15a5	solute carrier family 15, member 5	chr6	-4.731133154	0.00120820	9.931236506	0.00000019
18	Myoz1	myozenin 1	chr14	-4.198139313	0.00400330	4.115450402	0.00023289
19	Neb	nebulin	chr2	-3.97642231	0.00010067	6.946361658	0.00000000
20	Slc2a5	solute carrier family 2 member 5	chr4	-3.772616571	0.00130678	4.822316769	0.00000321
21	Marc1	mitochondrial amidoxime reducing component 1 (Mtarc1)	chr1	-3.70937506	0.00011831	3.852321405	0.00000015
22	Tfr2	transferrin receptor 2	chr5	-3.702884638	0.00008953	3.178095483	0.00036694
23	2310069B03Rik	RIKEN cDNA 2310069B03 gene	chr6	-3.430601918	0.00442447	2.879139611	0.00067184
24	Mlxipl	MLX interacting protein-like	chr5	-3.304635818	0.00000362	2.771832582	0.00014300
25	Hapln4	hyaluronan and proteoglycan link protein 4	chr8	-3.295089637	0.00001340	4.638451257	0.00012873
26	Pnpla3	patatin-like phospholipase domain containing 3	chr15	-3.27063947	0.00000031	2.301166383	0.00013270
27	Gm11520	predicted gene 11520	chr11	-3.193152912	0.00517541	2.603489383	0.00575359
28	Ttc25	outer dynein arm complex subunit 4 (Odad4)	chr11	-3.188548614	0.00282747	3.386327104	0.00003446
29	Fam57b	TLC domain containing 3B (Tlcd3b)	chr7	-3.051711678	0.00065974	2.534739256	0.00119985
30	Elovl3	ELOVL fatty acid elongase 3	chr19	-2.878781262	0.00170205	2.572605219	0.00055543
31	Odf3l1	outer dense fiber of sperm tails 3-like 1	chr9	-2.858605255	0.00210978	2.613562674	0.00334420
32	Angptl8	angiopoietin-like 8	chr9	-2.830001395	0.00044141	2.342879714	0.00000034
33	Acaca	acetyl-Coenzyme A carboxylase alpha	chr11	-2.825987526	0.00006213	2.328369192	0.00116391
34	Ctcfl	CCCTC-binding factor (zinc finger protein)-like	chr2	-2.822863407	0.00004196	3.147440361	0.00007848
35	Scd1	stearoyl-Coenzyme A desaturase 1	chr19	-2.79365452	0.00000214	2.024278423	0.00035163
36	Acly	ATP citrate lyase	chr11	-2.786751051	0.00024856	2.231941973	0.00211594
37	Acacb	acetyl-Coenzyme A carboxylase beta	chr5	-2.730347215	0.00006324	2.345124918	0.00069934
38	Fasn	fatty acid synthase	chr11	-2.711814031	0.00027666	2.360887753	0.00221614
39	Acot11	acyl-CoA thioesterase 11	chr4	-2.699798185	0.00033497	2.303976145	0.00054574
40	Hk2	hexokinase 2	chr6	-2.695666378	0.00000499	2.28503619	0.00003183
41	Ncan	neurocan	chr8	-2.670425807	0.00009096	13.10809959	0.00000233
42	Kng2	kininogen 2	chr16	-2.631043584	0.00190526	2.38354522	0.00392413
43	Impa2	inositol monophosphatase 2	chr18	-2.599388357	0.00033038	2.340899643	0.00138838
44	Ppp1r3b	protein phosphatase 1, regulatory subunit 3B	chr8	-2.585642632	0.00010491	1.868952864	0.00206128
45	Gm32200	predicted gene, 32200	chr1	-2.579560512	0.00059940	2.384251324	0.00031380
46	Plin2	perilipin 2	chr4	-2.57301374	0.00036310	2.56262567	0.00000827
47	Elovl6	ELOVL fatty acid elongase 6	chr3	-2.563104392	0.00178066	2.31783194	0.00214619
48	B430212C06Rik	RIKEN cDNA B430212C06 gene	chr18	-2.555155617	0.00465133	2.191188656	0.00387148
49	Deptor	DEP domain containing MTOR-interacting protein	chr15	-2.534030055	0.00010325	2.283292166	0.00060302
50	Orm1	orosomucoid 1	chr4	-2.487835722	0.00029616	1.930801808	0.00018227
51	Ybx2	Y box protein 2	chr11	-2.372547721	0.00068539	2.497398859	0.00016660
52	C7	complement component 7	chr15	-2.364556185	0.00299812	2.582352233	0.00371133
53	Me1	malic enzyme 1, NADP(+)-dependent, cytosolic	chr9	-2.312458522	0.00147527	2.197144045	0.00007772
54	Tmem79	transmembrane protein 79	chr3	-2.308283014	0.00247910	2.308631183	0.00046048
55	Mybpc2	myosin binding protein C, fast-type	chr7	-2.303774322	0.00788846	2.575690918	0.00017106
56	Nat8l	N-acetyltransferase 8-like	chr5	-2.303048643	0.00078200	2.36004515	0.00045022
57	Agpat2	1-acylglycerol-3-phosphate O-acyltransferase 2	chr2	-2.298558276	0.00191509	2.177002225	0.00036141
58	Lpin1	lipin 1	chr12	-2.294161149	0.00010579	1.851453715	0.00105545
59	Acss2	acyl-CoA synthetase short-chain family member 2	chr2	-2.292827529	0.00169793	1.829010795	0.00664079
60	Sbk1	SH3-binding kinase 1	chr7	-2.290899165	0.00041209	2.155875982	0.00016710
61	Pfkl	phosphofructokinase, liver, B-type	chr10	-2.271973256	0.00054044	2.306888751	0.00004527
62	Paqr9	progestin and adipoQ receptor family member IX	chr9	-2.265359905	0.00101696	1.911965951	0.00644233
63	Slc16a1	solute carrier family 16 member 1	chr3	-2.262846428	0.00097938	1.655724294	0.00883555
64	Sfxn5	sideroflexin 5	chr6	-2.252235361	0.00108356	1.833943093	0.00571332
65	Ibsp	integrin binding sialoprotein	chr5	-2.234168899	0.00231733	4.716266365	0.00000000
66	Gpd1	glycerol-3-phosphate dehydrogenase 1	chr15	-2.217866746	0.00302967	2.132792299	0.00334719
67	Plin5	perilipin 5	chr17	-2.191033023	0.00393885	1.996942247	0.00169445
68	Hoxc5	homeobox C5	chr15	-2.186362002	0.00085246	2.204316809	0.00391005
69	Tusc5	trafficking regulator of GLUT4 (SLC2A4) 1 (Trarg1)	chr11	-2.157952745	0.00113431	2.115138551	0.00027939
70	Acsf3	acyl-CoA synthetase family member 3	chr8	-2.132420125	0.00240348	2.222633743	0.00035874

Supplemental Table 2 (continued). A complete list of 150 genes whose mRNAs are downregulated by orchiectomy (Orx *vs.* sham Ctrl) but upregulated by exogenous dihydrotestosterone (DHT) administration to orchiectomized mice (Orx+DHT *vs.* Orx) in the aorta in mice administered Aldo-salt

#	Gene ID	Description	Location	Fold Change (Orx *vs.* Ctrl)	*P*-value	Fold Change (Orx+DHT *vs.* Orx)	*P*-value
71	Pgd	phosphogluconate dehydrogenase	chr4	-2.131726713	0.00205066	2.143337945	0.00010505
72	9330102E08Rik	RIKEN cDNA 9330102E08 gene	chr6	-2.107627848	0.00486940	2.024567569	0.00211630
73	Tkt	transketolase	chr14	-2.091740646	0.00409522	2.067176939	0.00206869
74	Acsl5	acyl-CoA synthetase long-chain family member 5	chr19	-2.078736676	0.00200660	1.853073813	0.00221677
75	Ddhd2	DDHD domain containing 2	chr8	-2.068611456	0.00072610	1.865787973	0.00224640
76	Slc25a1	solute carrier family 25 member 1	chr16	-2.03651381	0.00864575	2.51048562	0.00001006
77	Tmem120b	transmembrane protein 120B	chr5	-2.02086396	0.00287219	1.743871312	0.00334209
78	Comt	catechol-O-methyltransferase	chr16	-1.963952441	0.00138519	2.121874011	0.00000058
79	Aacs	acetoacetyl-CoA synthetase	chr5	-1.939758149	0.00247771	1.781540914	0.00417571
80	Tmem45b	transmembrane protein 45b	chr9	-1.93866892	0.00754666	1.588854032	0.00752814
81	Mecr	mitochondrial trans-2-enoyl-CoA reductase	chr4	-1.928501137	0.00395059	1.72435937	0.00619958
82	Hp	haptoglobin	chr8	-1.886586334	0.00279771	2.248206394	0.00012413
83	Hsd11b1	hydroxysteroid 11-beta dehydrogenase 1	chr1	-1.881418688	0.00007937	1.60069324	0.00378482
84	Hacd2	3-hydroxyacyl-CoA dehydratase 2	chr16	-1.867366825	0.00218642	1.617045546	0.00328187
85	Mrap	melanocortin 2 receptor accessory protein	chr16	-1.860112556	0.00563508	1.729569949	0.00198629
86	Kcnb1	potassium voltage-gated channel subfamily B member 1	chr2	-1.853407374	0.00063840	1.852136652	0.00426265
87	Lss	lanosterol synthase	chr10	-1.850836766	0.00013245	1.605997685	0.00385468
88	Rmdn3	regulator of microtubule dynamics 3	chr2	-1.848397365	0.00447196	1.572412075	0.00838924
89	Tlcd1	TLC domain containing 1	chr11	-1.840102931	0.00377380	1.710694622	0.00018632
90	Cnnm2	cyclin M2	chr19	-1.801393238	0.00000237	1.782632062	0.00004313
91	G6pdx	glucose-6-phosphate dehydrogenase X-linked	chrX	-1.800836729	0.00272730	1.543627875	0.00353357
92	Stradb	STE20-related kinase adaptor beta	chr1	-1.798053004	0.00119327	1.599447743	0.00099766
93	C1rl	complement component 1, r subcomponent-like	chr6	-1.784992951	0.00033755	1.509456118	0.00999971
94	Pcyt2	phosphate cytidylyltransferase 2, ethanolamine	chr11	-1.7697534	0.00657982	1.73544584	0.00268045
95	Nmnat1	nicotinamide nucleotide adenylyltransferase 1	chr4	-1.743290137	0.00188101	1.707795439	0.00008760
96	Lrrc39	eucine rich repeat containing 39	chr3	-1.740638829	0.00000018	1.386946913	0.00128862
97	Phlda3	pleckstrin homology like domain family A member 3	chr1	-1.716044185	0.00818734	1.743282126	0.00095372
98	Mgll	monoglyceride lipase	chr6	-1.683459704	0.00776891	1.576835916	0.00910642
99	Nfil3	nuclear factor, interleukin 3, regulated	chr13	-1.667030556	0.00111362	1.441425339	0.00225324
100	Mvd	mevalonate (diphospho) decarboxylase	chr8	-1.65412722	0.00378824	1.673206738	0.00221631
101	Cox19	cytochrome c oxidase assembly protein 19	chr5	-1.650729314	0.00767108	1.774601459	0.00030905
102	Slc22a3	solute carrier family 22 member 3	chr17	-1.630520927	0.00003373	1.740924376	0.00000000
103	Coa5	cytochrome C oxidase assembly factor 5	chr1	-1.624452201	0.00810975	1.5297709	0.00265661
104	Cars2	cysteinyl-tRNA synthetase 2 (mitochondrial)	chr8	-1.621565232	0.00555612	1.529314002	0.00289166
105	Carmn	cardiac mesoderm enhancer-associated non-coding RNA	chr18	-1.619787595	0.00197920	2.918660771	0.00000000
106	Itpk1	inositol 1,3,4-triphosphate 5/6 kinase	chr12	-1.589336596	0.00256452	1.473029058	0.00358650
107	Dpep1	dipeptidase 1	chr8	-1.5757984	0.00097956	1.735499686	0.00043256
108	Scd2	stearoyl-Coenzyme A desaturase 2	chr19	-1.574427077	0.00108712	1.46864475	0.00098439
109	Mmaa	metabolism of cobalamin associated A	chr8	-1.569681131	0.00551116	1.454749422	0.00191185
110	Ndufaf4	NADH:ubiquinone oxidoreductase complex assembly factor 4	chr4	-1.559775966	0.00267275	1.462207822	0.00604102
111	Mmd	monocyte to macrophage differentiation-associated	chr11	-1.559386139	0.00039233	1.426513229	0.00044902
112	Lurap1	leucine rich adaptor protein 1	chr4	-1.558933711	0.00040246	1.582664412	0.00783060
113	Cyb5b	cytochrome b5 type B	chr8	-1.542238773	0.00036187	1.384604221	0.00049918
114	Apol6	apolipoprotein L 6	chr15	-1.532663891	0.00115312	1.570024671	0.00010926
115	P2rx5	purinergic receptor P2X 5	chr11	-1.490944297	0.00565247	1.557318024	0.00080185
116	Pank3	pantothenate kinase 3	chr11	-1.48337874	0.00133920	1.517074363	0.00134805
117	Npr3	natriuretic peptide receptor 3	chr15	-1.478076943	0.00502678	1.426737408	0.00148496
118	Meg3	maternally expressed 3	chr12	-1.448072809	0.00171498	1.483402855	0.00161525
119	Ptger1	prostaglandin E receptor 1	chr8	-1.443791434	0.00543476	1.446960466	0.00175566
120	Srebf1	sterol regulatory element binding transcription factor 1	chr11	-1.440616238	0.00244150	1.400334518	0.00335320
121	Top1mt	DNA topoisomerase 1, mitochondrial	chr15	-1.439810381	0.00089557	1.582438847	0.00000808
122	Trp53inp2	tumor protein p53 inducible nuclear protein 2	chr2	-1.4131865	0.00055115	1.438719591	0.00013957
123	Agl	amylo-1,6-glucosidase, 4-alpha-glucanotransferase	chr3	-1.406107676	0.00004941	1.313159241	0.00460060
124	Kmt5a	lysine methyltransferase 5A	chr5	-1.392868198	0.00501356	1.618484378	0.00001105
125	H6pd	hexose-6-phosphate dehydrogenase (glucose 1-dehydrogenase)	chr4	-1.388417752	0.00061754	1.345726239	0.00237135
126	Slc26a6	solute carrier family 26, member 6	chr9	-1.368193118	0.00959822	1.390056813	0.00881876
127	Tmem94	transmembrane protein 94	chr11	-1.346056855	0.00578169	1.410066316	0.00926384
128	Zfp91	zinc finger protein 91	chr19	-1.339287698	0.00145760	1.28390934	0.00118220
129	Tesk1	testis specific protein kinase 1	chr4	-1.324693259	0.00535743	1.542268919	0.00000282
130	Dvl3	Dishevelled segment polarity protein 3	chr16	-1.317613868	0.00035895	1.384705362	0.00071284
131	Tubg1	tubulin, gamma 1	chr11	-1.302315434	0.00855233	1.35846039	0.00019502
132	Ankrd40	ankyrin repeat domain 40	chr11	-1.291592189	0.00224489	1.276077607	0.00068951
133	Ermp1	endoplasmic reticulum metallopeptidase 1	chr19	-1.276341119	0.00372563	1.452032333	0.00000013
134	Fam149b	family with sequence similarity 149, member B	chr14	-1.276200989	0.00061755	1.236998333	0.00150033
135	Usp10	ubiquitin specific peptidase 10	chr8	-1.264677609	0.00039713	1.248040584	0.00120024
136	Cipc	CLOCK interacting protein, circadian	chr12	-1.255642343	0.00250727	1.186484603	0.00809228
137	Tigar	Trp53 induced glycolysis regulatory phosphatase	chr6	-1.246052953	0.00380992	1.328222343	0.00024874
138	Med24	mediator complex subunit 24	chr11	-1.245250438	0.00001704	1.213322503	0.00006806
139	Mapk8ip3	mitogen-activated protein kinase 8 interacting protein 3	chr17	-1.237510664	0.00746874	1.325517425	0.00028239
140	Srr	serine racemase	chr11	-1.237403941	0.00932449	1.248915057	0.00383917

Supplemental Table 2 (continued). A complete list of 150 genes whose mRNAs are downregulated by orchiectomy (Orx *vs.* Sham Ctrl) but upregulated by exogenous dihydrotestosterone (DHT) administration to orchiectomized mice (Orx+DHT *vs.* Orx) in the aorta in mice administered Aldo-salt

#	Gene ID	Description	Location	Fold Change (Orx *vs.* Ctrl)	*P*-value	Fold Change (Orx+DHT *vs.* Orx)	*P*-value
141	Neurl4	neuralized E3 ubiquitin protein ligase 4	chr11	-1.227717106	0.00109247	1.298341332	0.00120645
142	Rnf10	ring finger protein 10 [	chr5	-1.2086912	0.00703013	1.325846168	0.00061523
143	Scaf1	SR-related CTD-associated factor	chr7	-1.207545164	0.00331215	1.290980285	0.00004449
144	Arfip2	ADP-ribosylation factor interacting protein 2	chr7	-1.202275336	0.00061851	1.254769016	0.00001189
145	Dvl1	dishevelled segment polarity protein	chr4	-1.190127928	0.00946186	1.271239888	0.00058910
146	Tab2	TGF-beta activated kinase 1/MAP3K7 binding protein 2	chr10	-1.189513717	0.00049606	1.152624365	0.00630599
147	Smg5	SMG5 nonsense mediated mRNA decay factor	chr3	-1.181871224	0.00798154	1.191993936	0.00409333
148	Mbd6	methyl-CpG binding domain protein 6	chr10	-1.175619455	0.00707640	1.360700607	0.00029690
149	Crebzf	CREB/ATF bZIP transcription factor	chr7	-1.173629123	0.00559876	1.185244594	0.00155486
150	R3hdm2	R3H domain containing 2	chr10	-1.140454302	0.00334356	1.222527753	0.00160605

Supplemental Table 3. A complete list of 65 signaling pathways upregulated by orchiectomy (Orx *vs.* Sham Ctrl) but downregulated by exogenous dihydrotestosterone (DHT) administration to orchiectomized mice (Orx+DHT *vs.* Orx) in the aorta in mice administered Aldo-salt

#	Pathway	*P*-value	Genes
1	Interleukin-2 signaling pathway	1.37E-16	Itk;Pcna;Txk;Tcf7;Cxcr5;Ptpn22;Ikzf3;Rasgrp1;Spn;Ctsk;Myb;Itgax;Gpc3;Il21r;Stat4;Pmaip1;Itgb7;Cytip;Ccr2;Cd52;Cr2;Sh2d1a;Cd2;Ptprc;Sell;Lck;Cd5;Il2rb;Pdcd4;Cd27;Irf5;Ptpn6;Ltb;Cd247;Cd69;Il7r;Lat;Il18r1
2	T helper cell surface molecules	2.19E-16	Cd2;Ptprc;Cd8a;Cd28;Cd3g;Cd247;Cd3e;Itgal;Cd3d
3	Adaptive immune system	5.13E-16	Blk;Itk;Cd3g;Kif11;Itgal;Cd3e;Rasgrp1;Cd3d;Cd79b;Cd79a;H2-DMa;Grap2;Cd19;Ctsk;Btla;Blnk;Itgb7;H2-Oa;H2-Ob;H2-Ab1;Cd74;H2-Eb2;Ptprc;Sell;Cd8a;Lck;Cd28;Ptpn6;Cd247;Card11;Lat;H2-Aa
4	T cell receptor signaling in naive CD4+ T cells	8.86E-16	Map4k1;Itk;Cd3g;Cd3e;Cd3d;Rasgrp1;Ptprc;Lck;Grap2;Cd28;Ptpn6;Cd247;Card11;Lat
5	T cell receptor signaling in naive CD8+ T cells	1.71E-15	Cd3g;Cd3e;Cd3d;Rasgrp1;Ptprc;Cd8a;Lck;Grap2;Cd28;Ptpn6;Cd247;Card11;Lat
6	Cell adhesion molecules (CAMs)	3.86E-15	H2-Eb2;Selplg;Itgal;Spn;Cd2;H2-DMa;Alcam;Ptprc;Sell;Cd8a;Cd28;Itgb7;H2-Oa;H2-Ob;H2-Ab1;Cd22;H2-Aa
7	Immune system	4.89E-15	Blk;Itk;Cd3g;Hmgb1;Kif11;Itgal;Cd3e;Rasgrp1;Cd3d;Cd79b;Cd79a;H2-DMa;Grap2;Cd19;Ctsk;Btla;Blnk;Itgb7;H2-Oa;H2-Ob;H2-Ab1;Ccr2;Cd74;H2-Eb2;Nup210;Ptprc;Sell;Cd8a;Lck;Il2rb;Cd28;Irf8;Irf5;Ptpn6;Cd247;Il7r;Card11;Lat;H2-Aa
8	Generation of second messenger molecules	1.14E-14	Itk;H2-Eb2;Lck;Grap2;Cd3g;Cd247;Cd3e;Cd3d;H2-Ab1;Lat;H2-Aa
9	PD-1 signaling	1.02E-13	H2-Eb2;Ptprc;Lck;Cd3g;Ptpn6;Cd247;Cd3e;Cd3d;H2-Ab1;H2-Aa
10	Primary immunodeficiency	4.10E-13	Cd79a;Ptprc;Cd8a;Lck;Cd19;Blnk;Tnfrsf13c;Cd3e;Il7r;Cd3d
11	Hematopoietic cell lineage	1.10E-12	Cr2;H2-Eb2;Cd3g;Cd3e;Cd3d;Cd2;Cd8a;Cd5;Cd19;Cd37;Il7r;Ms4a1;Cd22
12	CostimuLation by the Cd28 family	1.87E-12	H2-Eb2;Lck;Grap2;Btla;Cd28;Cd3g;Ptpn6;Cd247;Cd3e;Cd3d;H2-Ab1;H2-Aa
13	Leptin influence on immune response	2.04E-11	Mmp2;Cd3e;Cd3d;Cd79b;Cd79a;Azgp1;Alcam;Sell;Itgax;Irf8;Ltb;Il7r;Ccr2
14	T cell receptor signaling pathway	3.03E-11	Itk;Cd3g;Cd3e;Rasgrp1;Cd3d;Ptprc;Cd8a;Lck;Grap2;Cd28;Ptpn6;Cd247;Card11;Lat
15	T cell receptor reguLation of apoptosis	2.00E-10	Top2a;Lef1;Cd3g;Hmgb1;Itgal;Cd3e;Rasgrp1;Spn;Myb;Rac2;Il21r;Pmaip1;Ccr2;Map4k1;Cr2;Sema4d;Mmp2;Rhoh;Cd2;Ptprc;Lck;Cd28;Ltb;Il7r;H2-Aa
16	T cell signal transduction	2.12E-10	Lat2;Itk;Ptprc;Lck;Grap2;Cd28;Cd3g;Cd3e;Cd3d;Rasgrp1;Lat
17	Intestinal immune network for IgA production	3.93E-10	H2-DMa;H2-Eb2;Cd28;Itgb7;Tnfrsf13c;H2-Oa;H2-Ob;H2-Ab1;H2-Aa
18	T cell activation co-stimuLatory signal	4.45E-10	Itk;Lck;Cd28;Cd3g;Cd247;Cd3e;Cd3d
19	Lck and Fyn tyrosine kinases in initiation of T cell receptor activation	7.96E-10	Ptprc;Lck;Cd3g;Cd247;Cd3e;Cd3d
20	Interleukin-17 signaling pathway	2.29E-09	Cd2;Cd8a;Cd3g;Cd247;Cd3e;Cd3d
21	NO2-dependent IL-12 pathway in NK cells	5.57E-09	Cd2;Stat4;Cd3g;Cd247;Cd3e;Cd3d
22	Interleukin-12-mediated signaling events	6.57E-09	Cd8a;Lck;Il2rb;Stat4;Cd3g;Cd247;Cd3e;Cd3d;Il18r1
23	HIV-induced T cell apoptosis	6.83E-09	Cd28;Cd3g;Cd247;Cd3e;Cd3d
24	Asthma	7.27E-09	H2-DMa;H2-Eb2;Prg2;H2-Oa;H2-Ob;H2-Ab1;H2-Aa
25	Antigen-activated B-cell receptor generation of second messengers	7.55E-09	Blk;Map4k1;Cr2;Cd79b;Lat2;Cd79a;Ptprc;Cd19;Cd28;Rac2;Blnk;Ptpn6;Card11;Cd22
26	Inhibition of T cell receptor signaling by activated Csk	1.20E-08	Ptprc;Lck;Cd3g;Cd247;Cd3e;Cd3d
27	Viral myocarditis	1.46E-08	H2-DMa;H2-Eb2;Rac2;Cd28;Itgal;H2-Oa;H2-Ob;H2-Ab1;H2-Aa
28	MEF2D role in T cell apoptosis	2.31E-08	Ptprc;Lck;Cd3g;Cd247;Cd3e;Cd3d;Lat
29	Allograft rejection	3.49E-08	H2-DMa;H2-Eb2;Cd28;H2-Oa;H2-Ob;H2-Ab1;H2-Aa
30	CTL mediated immune response against target cells	6.78E-08	Cd3g;Cd247;Cd3e;Itgal;Cd3d
31	Graft-versus-host disease	7.39E-08	H2-DMa;H2-Eb2;Cd28;H2-Oa;H2-Ob;H2-Ab1;H2-Aa
32	Type 1 diabetes mellitus	1.04E-07	H2-DMa;H2-Eb2;Cd28;H2-Oa;H2-Ob;H2-Ab1;H2-Aa
33	Interleukin-12/Stat4 pathway	1.45E-07	Stat4;Cd28;Cd3g;Cd247;Cd3e;Cd3d;Il18r1
34	Cell surface interactions at the vascular wall	1.75E-07	Spn;Cd2;Selplg;Sell;Lck;Itgax;Ptpn6;Itgal;Slc7a10
35	MHC class II antigen presentation	3.85E-07	Cd74;H2-Eb2;H2-DMa;Ctsk;Kif11;H2-Oa;H2-Ob;H2-Ab1;H2-Aa
36	Autoimmune thyroid disease	4.05E-07	H2-DMa;H2-Eb2;Cd28;H2-Oa;H2-Ob;H2-Ab1;H2-Aa
37	Tob role in T-cell activation	5.86E-07	Cd28;Cd3g;Cd247;Cd3e;Cd3d
38	Antigen processing and presentation	6.77E-07	Cd74;H2-DMa;H2-Eb2;Cd8a;H2-Oa;H2-Ob;H2-Ab1;H2-Aa
39	Hemostasis pathway	1.20E-06	Selplg;Prcp;Kif11;Slc7a10;Itgal;Rasgrp1;Spn;Cd2;Sell;Kifc1;Lck;Myb;Alb;Rac2;Itgax;Ptpn6;Lat
40	ImmunoreguLatory interactions between a lymphoid and a non-lymphoid cell	1.31E-06	Sell;Cd8a;Cd19;Cd3g;Itgb7;Cd247;Cd3e;Itgal;Cd3d
41	CD8/T cell receptor downstream pathway	2.60E-06	Cd8a;Il2rb;Stat4;Cd3g;Cd247;Cd3e;Cd3d
42	Leishmaniasis	3.83E-06	H2-DMa;H2-Eb2;Ptpn6;H2-Oa;H2-Ob;H2-Ab1;H2-Aa
43	Interleukin-4 reguLation of apoptosis	5.71E-06	Top2a;Arl4c;Pou2af1;Tcf7;Mcm3;Cd27;Mcm4;Ltb;Il7r;Rasgrp1;Chst2;H2-Aa
44	Stathmin and breast cancer resistance to antimicrotubule agents	7.85E-06	Cd2;Cd3g;Cd247;Cd3e;Cd3d
45	CXCR4 signaling pathway	1.02E-05	Blk;Ptprc;Lck;Cd3g;Ptpn6;Cd247;Cd3e;Cd3d
46	Alpha-M beta-2 integrin signaling	3.22E-05	Blk;Selplg;Lck;Mmp2;Hmgb1
47	Immune system signaling by interferons, interleukins, prolactin, and growth hormones	4.86E-05	H2-Eb2;Nup210;Lck;Il2rb;Blnk;Irf8;Irf5;Ptpn6;Il7r;H2-Ab1;H2-Aa
48	Ras-independent pathway in NK cell-mediated cytotoxicity	5.86E-05	Cd2;Cd28;Ptpn6;Lat
49	Apoptotic DNA fragmentation and tissue homeostasis	8.21E-05	Top2a;Hmgb2;Hmgb1
50	Leukocyte transendothelial migration	9.18E-05	Itk;Txk;Mmp2;Rac2;Rhoh;Ezr;Itgal

Supplemental Table 3 (continued). A complete list of 65 signaling pathways upregulated by orchiectomy (Orx *vs.* Sham Ctrl) but downregulated by exogenous dihydrotestosterone (DHT) administration to orchiectomized mice (Orx+DHT *vs.* Orx) in the aorta in mice administered Aldo-salt

#	Pathway	*P*-value	Genes
51	B lymphocyte cell surface molecules	1.12E-04	Cr2;Ptprc;Itgal
52	Cytokine-cytokine receptor interaction	1.49E-04	Il2rb;Cxcr5;Il21r;Cd27;Tnfrsf13c;Ltb;Cxcr6;Il7r;Il18r1;Ccr2
53	Natural killer cell receptor signaling pathway	2.04E-04	Lat2;Lck;Rac2;Ptpn6;Lat
54	T cell receptor/JNK pathway	2.43E-04	Map4k1;Grap2;Lat
55	Natural killer cell-mediated cytotoxicity	2.45E-04	Lck;Sh2d1a;Rac2;Ptpn6;Cd247;Itgal;Lat
56	Interferon-gamma signaling pathway	2.45E-04	H2-Eb2;Irf8;Irf5;Ptpn6;H2-Ab1;H2-Aa
57	Systemic lupus erythematosus	2.68E-04	H2-Eb2;H2-DMa;Cd28;H2-Oa;H2-Ob;H2-Ab1;H2-Aa
58	Adhesion and diapedesis of granulocytes	3.01E-04	Selplg;Sell;Itgal
59	Signaling by the B cell receptor (BCR)	4.42E-04	Blk;Cd79b;Cd79a;Cd19;Blnk;Rasgrp1;Card11
60	Dendritic cells in reguLating TH1 and TH2 development	4.44E-04	Cd2;Cd5;Itgax
61	Phagosome	4.98E-04	H2-Eb2;H2-DMa;H2-Oa;H2-Ob;Coro1a;H2-Ab1;H2-Aa
62	Interferon signaling	8.33E-04	H2-Eb2;Nup210;Irf8;Irf5;Ptpn6;H2-Ab1;H2-Aa
63	Interleukin-2 receptor beta chain in T cell activation	1.23E-03	Pcna;Il2rb;Ptpn6;Ikzf3
64	Endogenous Toll-like receptor signaling	1.26E-03	Hmgb1;S100a9;S100a8
65	Focal adhesion	1.26E-03	Blk;Parvg;Txk;Col11a1;Rac2;Itgax;Itgb7;Itgal

Supplemental Table 4. A complete list of 19 signaling pathways downregulated by orchiectomy (Orx *vs.* Sham Ctrl) but upregulated by exogenous dihydrotestosterone (DHT) administration to orchiectomized mice (Orx+DHT *vs.* Orx) in the aorta in mice administered Aldo-salt

#	Pathway	*P*-value	Genes
1	Triglyceride biosynthesis	1.64E-13	Slc25a1;Acly;Fasn;Elovl3;Gpd1;Acsl5;Elovl6;Lpin1;Agpat2;Acaca
2	Fatty acid, triacylglycerol, and ketone body metabolism	3.63E-12	Slc25a1;Srebf1;Elovl3;Acsl5;Elovl6;Acacb;Agpat2;Acaca;Acly;Med24;Fasn;Gpd1;Me1;Plin2;Lpin1
3	Fatty acyl-CoA biosynthesis	3.44E-11	Slc25a1;Acly;Fasn;Elovl3;Acsl5;Elovl6;Acaca
4	Lipid and lipoprotein metabolism	1.13E-10	Slc25a1;Srebf1;Pcyt2;Elovl3;Acsl5;Elovl6;Acacb;Lss;Agpat2;Acaca;Hsd11b1;Acly;Med24;Fasn;Gpd1;Me1;Pnpla3;Plin2;Mvd;Lpin1;Mgll
5	Fatty acid biosynthesis	4.98E-10	Acly;Acss2;Mecr;Fasn;Acsl5;Acacb;Acaca
6	ChREBP activates metabolic gene expression	1.22E-09	Acly;Mlxipl;Fasn;Acacb;Acaca
7	Metabolism	2.72E-08	Pank3;H6pd;Acss2;Ncan;Comt;Acacb;Agpat2;Acaca;Hk2;Hsd11b1;Impa2;Me1;Mecr;Pcyt2;Kcnb1;Agl;Elovl3;Itpk1;Acsl5;Elovl6;Pgd;Lss;Acly;Mlxipl;Pfkl;Med24;Nmnat1;Fasn;Gpd1;Pnpla3;Mvd;Tkt;Lpin1;Mgll
8	Striated muscle contraction	9.12E-06	Mybpc1;Mybpc2;Actn3;Tnnc2;Neb
9	Shuttle for transfer of acetyl groups from mitochondria to the cytosol	2.25E-05	Slc25a1;Acly;Me1
10	Muscle contraction	3.24E-05	Mybpc1;Mybpc2;Actn3;Tnnc2;Neb
11	Ghrelin pathway	3.40E-05	Srebf1;Fasn;Plin2;Acaca
12	Pyruvate metabolism	4.34E-05	Acss2;Slc16a1;Me1;Acacb;Acaca
13	Integration of energy metabolism	4.42E-05	Acly;Mlxipl;Kcnb1;Fasn;Tkt;Acacb;Acaca
14	Glycerophospholipid biosynthesis	4.62E-05	Pcyt2;Gpd1;Pnpla3;Lpin1;Agpat2;Mgll
15	Pentose phosphate pathway	4.66E-05	Pfkl;H6pd;Pgd;Tkt
16	AMPK signaling	1.58E-04	Srebf1;Stradb;Fasn;Acacb;Acaca
17	Carbohydrate metabolism	4.05E-04	Slc25a1;Pfkl;Ncan;Agl;Pgd;Slc2a5;Tkt;Hk2
18	Adipogenesis	5.05E-04	Srebf1;Dvl1;Pnpla3;Plin2;Lpin1;Agpat2
19	Acyl chain remodeling of diacylglycerol and triacylglycerol	5.51E-04	Pnpla3;Mgll

Supplemental Table 5. A complete list of T cell subsets analyzed by flow cytometry in the aorta in sham-operated and orchiectomized (Orx) mice administered Aldo-salt with and without dihydrotestosterone (DHT)

		# of T cells / aorta			% of T cells		
Subsets	CD markers	Sham	Orx	Orx+DHT	Sham	Orx	Orx+DHT
T cells	$CD45^{+}CD3^{+}$	1,198±362	7,597±3066	3,381±1560	56.40±5.84%	72.17±4.07%*	65.34±4.14%
CD4 T cells	$CD45^{+}CD3^{+}CD4^{+}$	245±76	2,537±1076	932±451	22.40±2.28%	31.70±1.68%**, #	26.04±1.19%
CD8 T cells	$CD45^{+}CD3^{+}CD8^{+}$	490±129	4,165±1817	1,686±867	42.55±2.95%	47.92±3.15%	42.26±3.00%
Naïve CD4 T cells	$CD45^{+}CD3+CD4+CD44^{-}CD62L^{+}$	89±25	1,808±838	593±322	34.51±7.03%	59.37±4.14%**	49.55±5.46%
Naïve CD8 T cells	$CD45^{+}CD3^{+}CD8+CD44^{-}CD62L^{+}$	122±37	2,532±1267	805±478	23.49±5.57%	46.83±4.46%**	34.39±4.42%
Central memory CD4 T cells	$CD45^{+}CD3^{+}CD4^{+}CD44^{+}CD62L^{+}$	9±3	81±37	44±23	3.66±1.09%	5.10±0.82%	4.92±0.48%
Central memory CD8 T cells	$CD45^{+}CD3^{+}CD8^{+}CD44^{+}CD62L^{+}$	94±29	961±406	460±253	16.29±5.02%	22.07±2.01%	21.46±2.62%
Effector CD4 T cells	$CD45^{+}CD3^{+}CD4^{+}CD44^{+}CD62L^{-}CD127^{-}$	72±28	222±66	129±57	27.75±3.85%	13.60±1.66%***	17.52±2.18%
Effector CD8 T cells	$CD45^{+}CD3^{+}CD8^{+}CD44^{+}CD62L^{-}CD127^{-}$	198±75	316±87	225±74	44.20±10.16%	16.73±3.22%**	27.14±4.23%
Effector memory CD4 T cells	$CD45^{+}CD3^{+}CD4^{+}CD44^{+}CD62L^{-}CD127^{+}$	41±15	95±20	70±22	20.35±6.68%	9.57±2.03%*	13.04±2.50%
Effector memory CD8 T cells	$CD45^{+}CD3^{+}CD8^{+}CD44^{+}CD62L^{-}CD127^{+}$	22±6	80±22	56±21	5.63±1.32%	4.03±0.89%	5.76±1.25%
$PD-1^{+}$ T cells	$CD45^{+}CD3^{+}PD-1^{+}$	405±137	966±295	563±207	34.60±3.87%	19.02±2.50%**	25.13±3.53%
$PD-1^{+}$ CD4 T cells	$CD45^{+}CD3^{+}CD4^{+}PD-1^{+}$	66±22	254±98	137±63	27.55±3.41%	17.69±1.80%*	14.91±2.79%
$PD-1^{+}$ CD8 T cells	$CD45^{+}CD3^{+}CD8^{+}PD-1^{+}$	172±66	321±96	213±82	34.83±6.11%	13.15±2.06%**, #	25.11±4.89%
$PD-1^{+}$ naïve CD4 T cells	$CD45^{+}CD3^{+}CD4^{+}CD44^{-}CD62L^{+}PD-1^{+}$	0	9±5	4±3	0.11±0.11%	0.52±0.15%	0.21±0.14%
$PD-1^{+}$ naïve CD8 T cells	$CD45^{+}CD3^{+}CD8^{+}CD44^{-}CD62L^{+}PD-1^{+}$	1±1	4±3	2±1	0.65±0.30%	0.48±0.21%	0.05±0.04%
$PD-1^{+}$ central memory CD4 T cells	$CD45^{+}CD3^{+}CD4^{+}CD44^{+}CD62L^{+}PD-1^{+}$	0	16±8	6±3	1.28±1.28%	12.37±3.52%*	6.10±2.53%
$PD-1^{+}$ central memory CD8 T cells	$CD45^{+}CD3^{+}CD8^{+}CD44^{+}CD62L^{+}PD-1^{+}$	2±1	11±6	5±2	1.18±0.58%	2.72±0.72%	1.12±0.59%
$PD-1^{+}$ effector CD4 T cells	$CD45^{+}CD3^{+}CD4^{+}CD44^{+}CD62L^{-}CD127^{-}PD-1^{+}$	9±2	78±31	32±16	22.59±7.08%	31.10±4.59%	18.61±3.68%
$PD-1^{+}$ effector CD8 T cells	$CD45^{+}CD3^{+}CD8^{+}CD44^{+}CD62L^{-}CD127^{-}PD-1^{+}$	112±50	111±37	85±33	55.18±7.69%	29.77±3.72%**	39.06±6.28%
$PD-1^{+}$ effector memory CD4 T cells	$CD45^{+}CD3^{+}CD4^{+}CD44^{+}CD62L^{-}CD127^{+}PD-1^{+}$	8±3	9±2	6±3	23.23±9.00%	9.49±1.77%*	4.91±1.74%
$PD-1^{+}$ effector memory CD8 T cells	$CD45^{+}CD3^{+}CD8^{+}CD44^{+}CD62L^{-}CD127^{+}PD-1^{+}$	3±1	10±3	10±5	14.05±3.11%	12.80±2.37%	19.55±5.34%

Note: % of T cells, CD4 T cells, and CD8 T cells were calculated in total leukocytes ($CD45^{+}$) and T cells ($CD45^{+}CD3^{+}$), respectively. % of other T cell subsets were calculated in total CD4 and CD8 T cells, respectively. % of $PD-1^{+}$ T cell subsets were calculated in total corresponding T cell subsets as indicated, respectively. The data were expressed as mean ± SEM (n = 6-10/group). * indicates the statistically significant difference between Orx and Sham; # indicates the statistically significant difference between Orx and Orx+DHT.

Supplemental Table 6. A complete list of T cell subsets analyzed by flow cytometry in the spleen in sham-operated and orchiectomized (Orx) mice administered Aldo-salt with and without dihydrotestosterone (DHT)

		# of T cells / spleen			% of T cells		
Subsets	CD markers	Sham	Orx	Orx+DHT	Sham	Orx	Orx+DHT
T cells	CD45$^+$CD3$^+$	6,281,657±592,168	8,973,344±693,080*	8,767,771±835,538	26.04±1.65%	24.74±1.40%	25.13±0.74%
CD4 T cells	CD45$^+$CD3$^+$CD4$^+$	3,107,685±279,592	4,727,726±375,913*	4,473,675±424,215	49.69±0.82%	52.74±0.79%*	51.04±0.53%
CD8 T cells	CD45$^+$CD3$^+$CD8$^+$	2,557,869±281,336	3,290,791±285,030	3,488,653±351,655	40.26±1.09%	36.42±0.84%*	39.73±0.82%
Naïve CD4 T cells	CD45$^+$CD3+CD4+CD44$^-$ CD62L$^+$	1,747,585±258,488	2,678,433±349,451	2,856,478±298,692	54.21±4.08%	55.01±2.80%	63.84±2.49%
Naïve CD8 T cells	CD45$^+$CD3$^+$CD8+CD44$^-$ CD62L$^+$	1,600,050±215,032	2,062,969±235,527	2,320,566±295,875	61.20±2.95%	61.63±1.98%	65.59±2.85%
Central memory CD4 T cells	CD45$^+$CD3$^+$CD4$^+$CD44$^+$ CD62L$^+$	214,107±18,989	368,163±23,427**	286,068±40,509	7.13±0.67%	7.99±0.56%	6.22±0.60%
Central memory CD8 T cells	CD45$^+$CD3$^+$CD8$^+$CD44$^+$ CD62L$^+$	702,849±71,070	838,956±47,693	826,982±82,651	28.73±2.73%	26.37±1.56%	24.17±2.22%
Effector CD4 T cells	CD45$^+$CD3$^+$CD4$^+$CD44$^+$ CD62L$^-$CD127$^-$	471,853±41,692	786,737±41,703$^{***,\ \#\#}$	543,635±73,548	16.25±2.18%	17.67±1.82%	12.09±1.09%
Effector CD8 T cells	CD45$^+$CD3$^+$CD8$^+$CD44$^+$ CD62L$^-$CD127$^-$	83,802±7,404	143,744±14,892**	112,505±15,606	3.43±0.27%	4.50±0.39%$^{\#}$	3.21±0.30%
Effector memory CD4 T cells	CD45$^+$CD3$^+$CD4$^+$CD44$^+$ CD62L$^-$CD127$^+$	319,502±30,085	474,058±36,190*	37,3531±61,183	10.70±1.09%	10.27±0.81%	8.28±1.25%
Effector memory CD8 T cells	CD45$^+$CD3$^+$CD8$^+$CD44$^+$ CD62L$^-$CD127$^+$	89,860±15,235	131,115±17,435	107,564±13,502	3.52±0.45%	3.99±0.44%	3.31±0.61%
PD-1$^+$ CD4 T cells	CD45$^+$CD3$^+$CD4$^+$PD-1$^+$	732,907±65,432	1,197,386±82,167**	873,180±159,323	24.76±2.78%	25.86±1.68%	19.13±3.11%
PD-1$^+$ CD8 T cells	CD45$^+$CD3$^+$CD8$^+$PD-1$^+$	119,045±15,451	178,680±22,632	177,581±35,976	4.65±0.27%	5.43±0.61%	5.23±1.09%
PD-1$^+$ naïve CD4 T cells	CD45$^+$CD3$^+$CD4$^+$CD44$^-$ CD62L$^+$PD-1$^+$	51,792±4,758	95,330±18,486*	75,078±10,528	3.48±0.66%	3.54±0.32%	2.62±0.22%
PD-1$^+$ naïve CD8 T cells	CD45$^+$CD3$^+$CD8$^+$CD44$^-$ CD62L$^+$PD-1$^+$	15,425±1,577	27,326±2,040**	24,588±3,484	1.08±0.14%	1.42±0.13%	1.07±0.07%
PD-1$^+$ central memory CD4 T cells	CD45$^+$CD3$^+$CD4$^+$CD44$^+$ CD62L$^+$PD-1$^+$	67,471±8,893	121,917±7,785**	91,375±17,072	31.18±2.74%	33.12±0.81%	30.44±2.23%
PD-1$^+$ central memory CD8 T cells	CD45$^+$CD3$^+$CD8$^+$CD44$^+$ CD62L$^+$PD-1$^+$	13,811±2,072	17,439±1,847	15,041±2,409	1.97±0.24%	2.07±0.17%	1.77±0.16%
PD-1$^+$ effector CD4 T cells	CD45$^+$CD3$^+$CD4$^+$CD44$^+$ CD62L$^-$CD127$^-$PD-1$^+$	224,723±22,003	347,489±21,041$^{*,\ \#}$	242,495±40,599	47.49±2.42%	44.07±0.87%	43.61±2.46%
PD-1$^+$ effector CD8 T cells	CD45$^+$CD3$^+$CD8$^+$CD44$^+$ CD62L$^-$CD127$^-$PD-1$^+$	26,300±2,874	42,390±5,493	41,028±9,156	31.86±3.28%	29.57±2.83%	35.73±6.08%
PD-1$^+$ effector memory CD4 T cells	CD45$^+$CD3$^+$CD4$^+$CD44$^+$ CD62L$^-$CD127$^+$PD-1$^+$	68,882±11,774	76,937±11,718	82,860±22,206	20.98±1.71%	15.82±1.47%	19.83±2.50%
PD-1$^+$ effector memory CD8 T cells	CD45$^+$CD3$^+$CD8$^+$CD44$^+$ CD62L$^-$CD127$^+$PD-1$^+$	10,537±1,465	14,155±2,333	17,336±3,563	12.47±1.34%	10.52±0.83%$^{\#}$	16.37±2.34%

Note: % of T cells, CD4 T cells, and CD8 T cells were calculated in total leukocytes (CD45$^+$) and T cells (CD45$^+$CD3$^+$), respectively. % of other T cell subsets were calculated in total CD4 and CD8 T cells, respectively. % of PD-1$^+$ T cell subsets were calculated in total corresponding T cell subsets as indicated, respectively. The data were expressed as mean ± SEM (n = 7-9/group). * indicates the statistically significant difference between Orx and Sham; # indicates the statistically significant difference between Orx and Orx+DHT.

Supplemental Table 7. A complete list of antibodies used in the current study

Antibody	Application	Source/ Reactivity	Manufacture	Catalog	Dilution/amount	Fluorophore	Reference
AR	IHC	Rabbit anti-mouse	Abcam	Ab74272	1:300		Dufour et al., Cell Reports. 2022;38, 110534
AR	WB	Rabbit anti-human	Cell Signaling	5153	1:2000		Li et al. Maturitas.2009; 63, 142-148.
IL-6	IHC	Rabbit anti-mouse	Bioss	Bs-0782R	1:2000		Sun et al., Biomed Res Int. 2015;919401
MR	IHC	Mouse anti-mouse	DSHB	rMR1-18 1D5	1.1 μg/ml		Prager et al., PLoS One. 2010;5(12):e14344
p-STAT3	IHC	Rabbit anti-mouse	Cell Signaling	9145	1:400		Cheng et al., Nat Commun. 2022;29;13(1):4418
F4/80	IHC	Rabbit anti-mouse	Cell Signaling	70076	1:1000		Wang et al., Front Immunol. 2022;13:901209
Ly6G	IHC	Rat anti-mouse	BD Pharmingen	551459	1:1500		Fleming, et. al. J Immunol. 1993; 151(5):2399-2408
PD-1	WB, IHC	Rabbit anti-mouse	Cell Signaling	84651	WB 1:1000<br>IHC 1:250		Yuan et al., JCI insight. 2022;7(11):e157788.
PD-1	IHC	Rabbit anti-human	Cell Signaling	86163	IHC 1:250		Ju et al., Nat Commun. 2022; 13(1):5378
CD3e	WB, IHC	Rabbit anti-mouse	Cell Signaling	99940	WB 1:1000<br>IHC 1:500		Wallace-Povirk et al., Sci Rep. 2022;5;12(1):11346
CD19	WB, IHC	Rabbit anti-mouse	Cell Signaling	90176	WB 1:1000<br>IHC 1:2000		Tedder et al. Immunity. 1997;6, 107-18.
GAPDH	WB	Rabbit anti-mouse	Cell Signaling	2118	1:5000		Wong et al., J Clin Invest. 2022;132(15):e152635
AR	ChIP	Mouse anti-mouse	Santa Cruz	sc7305	5 μg/ChIP		Sharma et al., Front Oncol. 2022;12: 824594
AR	ChIP	Rabbit anti-mouse	Millipore	17-10489	1 μg/ChIP		Nigro et al., Diabetes. 2021;70(6):1250–1264
CD45	FCM	Rat anti-mouse	Biolegend	103128	0.25 μg/10^6 cells	Alexa Fluor 700	Tian et al., Nat Commun. 2016;7:13283.
CD45	FCM	Rat anti-mouse	Biolegend	103126	0.25 μg/10^6 cells	Pacific blue	Pattabiraman et al., Science. 2016;351(6277):aad3680
CD45	FCM	Rat anti-mouse	Biolegend	103131	0.25 μg/10^6 cells	PerCP/Cyanine5.5	Radtke AJ, et al. 2020. Proc Natl Acad Sci U S A. 117:33455-65.
CD3ε	FCM	Rat anti-mouse	Biolegend	100320	0.5 μg/10^6 cells	PE/Cy7	Cabañero et al. Elife. 2020;9:e55582
CD3	FCM	Rat anti-mouse	Biolegend	100240	0.25 μg/10^6 cells	Alexa Fluor 594	Lederer et al., Immunity. 2020;53(6):1281-1295.e5.
CD4	FCM	Rat anti-mouse	BD Horizon	563790	0.25 μg/10^6 cells	BUV395	Wineman JP et al. Blood. 1992; 180(7):1717-1724.
CD8a	FCM	Rat anti-mouse	Biolegend	100793	0.25 μg/10^6 cells	Spark Blue 574	Radtke AJ, et al. 2022. Nat Protoc. 17:378-401.
CD44	FCM	Rat anti-mouse	Biolegend	103028	1 μg/10^6 cells	APC/Cyanine7	Tay S, et al. 2014. Proc Natl Acad Sci U S A. 111:2540.
CD62L	FCM	Rat anti-mouse	Biolegend	104436	0.125 μg/10^6 cells	BV421	Bergot AS, et al. 2020. J Immunol. 204:1787.
CD127	FCM	Rat anti-mouse	Biolegend	135018	2 μg/10^6 cells	Alexa Fluor 488	Yamaguchi A, et al. 2021. Genes Cells. 26:782.
PD-1	FCM	Rat anti-mouse	Biolegend	135210	0.25 μg/10^6 cells	APC	Mandal et al. Cell Reports. 2021;35(6):109094.
PD-1	FCM	Rat anti-mouse	Biolegend	135219	0.125 μg/10^6 cells	BV605	Mogilenko et al., Immunity 2021;54(1):99-115.e12
CD19	FCM	Rat anti-mouse	Biolegend	115534	0.25 μg/10^6 cells	PerCP	Faust et al., J Clin Invest. 2020;130(10):5493-5507
Ly6G	FCM	Rat anti-mouse	Biolegend	127608	0.25 μg/10^6 cells	PE	Okada et al., J Biol Chem. 2014;289(47):32926-36
F4/80	FCM	Rat anti-mouse	Biolegend	123120	1 μg/10^6 cells	Alexa Fluor 488	Wheeler et al., Nat Commun. 2015;6: 8964.
F4/80	FCM	Rat anti-mouse	Biolegend	123110	1 μg/10^6 cells	PE	Xiang et al., PNAS 2014;111(48):E5159-68

Note: IHC, immunocytochemistry. WB, western blot. ChIP, Chromatin immunoprecipitation. FCM, flow cytometry.

Supplemental Table 8. A complete list of mouse PCR primers used in the current study

Gene	Primer	Sequence	Application
Ar	Forward	5'-GGACCATGTTTTACCCATCG-3'	Real-time PCR
	Reverse	5'-TCGTTTCTGCTGGCACATAG-3'	
Bmal1	Forward	5'-ATCAGCGACTTCATGTCTCC-3'	Real-time PCR
	Reverse	5'-CTCCCTTGCATTCTTGATCC-3'	
36B4	Forward	5'-CCCTGAAGTGCTCGACATCA-3'	Real-time PCR
	Reverse	5'-TGCGGACACCCTCCAGAA-3'	
Ccl2	Forward	5'-CTTCCTCCACCACCATGCA-3'	Real-time PCR
	Reverse	5'-CCAGCCGGCAACTGTGA-3'	
Ccl4	Forward	5'-TTCCTGCTGTTTCTCTTACACCT-3'	Real-time PCR
	Reverse	5'-CTGTCTGCCTCTTTTGGTCAG-3'	
Cyp17a1	Forward	5'-AGTGCTCGTGAAGAAGGGGA-3'	Real-time PCR
	Reverse	5'-TTTCCTTGGTCCGACAAGAGG-3'	
Hsd17b3	Forward	5'-AGGTTCTCGCAGCACCTTTTT-3'	Real-time PCR
	Reverse	5'-CATCGCCTGCTCCGGTAATC-3'	
Hsd3b2	Forward	5'-GGTTTTTGGGGCAGAGGATCA-3'	Real-time PCR
	Reverse	5'-GGTACTGGGTGTCAAGAATGTCT-3'	
Il1b	Forward	5'-TCGCTC AGGGTCACAAGAAA-3'	Real-time PCR
	Reverse	5'-CATCAGAGGCAAGGAGGAAAAC-3'	
Il6	Forward	5'-ACAAGTCGGAGGCTTAATTACACAT-3'	Real-time PCR
	Reverse	5'-TTGCCATTGCACAACTCTTTTC-3'	
Il6ra	Forward	5'-AGCGACACTGGGGACTATTTA-3'	Real-time PCR
	Reverse	5'-ACAGCCTTCGTGGTTGGAG-3'	
Il6st	Forward	5'-TGGAGTGAGGAGGCTAGTGG-3'	Real-time PCR
	Reverse	5'-ATTTTCCCATTGGCTTCAGA-3'	
Mmp2	Forward	5'-GGACAAGTGGTCCGCGTAAA-3'	Real-time PCR
	Reverse	5'-CCGACCGTTGAACAGGAAGG-3'	
Nr3c2	Forward	5'-ATGGGTACCCGGTCCTAGAG-3'	Real-time PCR
	Reverse	5'-ACCAAGCAGATCTTGGAAGG-3'	
Pdcd1	Forward	5'-CACTATCCCACTGACCCTTCA-3'	Genotyping
	WT reverse	5'-AGAAGGTGAGGGACCTCCAG-3'	
	Mutant reverse	5'-CACAGGGTAGGCATGTAGCA-3'	
Pdcd1	Forward	5'-CCTTTAGCTTCTGGGAAATGTTT-3'	ChIP Exon 4
	Reverse	5'-CCCTGGAGGTAATGGCAAGTTTCC-3'	
Pdcd1	Forward	5'-TCATTCCACTCACAAGTCAATCAA-3'	ChIP Exon 6
	Reverse	5'-TCTTCCCTTCTCATCTCATTGTGA-3'	
Pdcd1	Forward	5'-ACCCTGGTCATTCACTTGGG-3'	Real-time PCR
	Reverse	5'- CATTTGCTCCCTCTGACACTG-3'	
Scnn1a	Forward	5'-TACTTCAGCTACCCCGTGAGT-3'	Real-time PCR
	Reverse	5'-AAAAAGCGTCTGTTCCGTGAT-3'	
Scnn1b	Forward	5'-ACCCGGTGGTTCTCAATTTGT-3'	Real-time PCR
	Reverse	5'-AAGTTCCGCAAGGTACACACA-3'	
Scnn1g	Forward	5'-GGCACCGACCATTAAGGACC-3'	Real-time PCR
	Reverse	5'-CTGTCAGCGTGAACGCAATC-3'	
Sgk1	Forward	5'-TCAGAGCGGAATGTTCTGTTG-3'	Real-time PCR
	Reverse	5'-AGCGGTCTGGAATGAGAAGTG-3'	
Tgfb2	Forward	5'-CCATCCCGCCCACTTTCTAC-3'	Real-time PCR
	Reverse	5'-TCTGGTTTTCACAACCTTGCT-3'	
Tnf	Forward	5'-CCCTCACACTCAGATCATCTTCT-3'	Real-time PCR
	Reverse	5'-GCTACGACGTGGGCTACAG-3'	
