# Supplementary material for: Androgen aggravates aortic aneurysms via suppression of PD-1 in mice
Source: J Clin Invest. 2024 Jun 20;134(15):e169085. doi: 10.1172/JCI169085 (PMC11290977; doi:10.1172/JCI169085)
Supplement: Unedited blot and gel images [file jci-134-169085-s206.pdf]

Uncropped/unedited images for all blots and gels in the manuscript

Full Unedited Immunoblot for Figure 9E

PD-1 Immunoblot

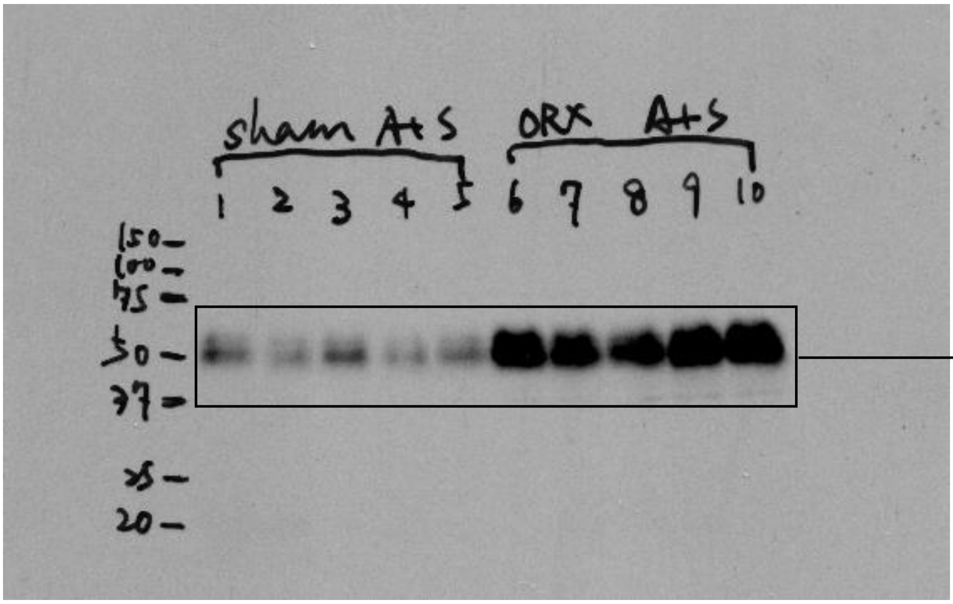

CD3ε Immunoblot

αPD-1 residual after stripping

Non-specific

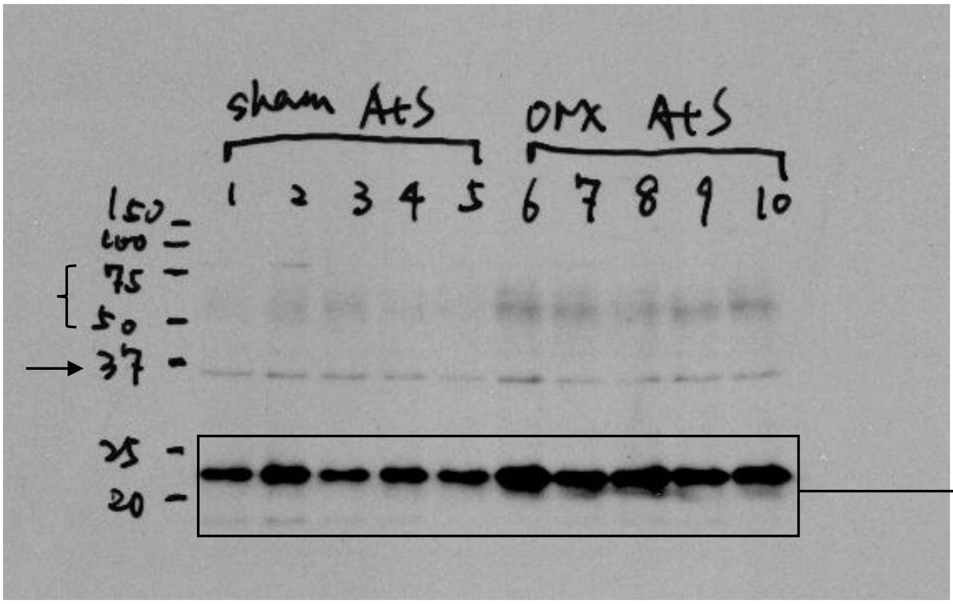

Representative Immunoblot Shown in Figure 9E

Full Unedited Immunoblot for Figure 9E

CD19 Immunoblot

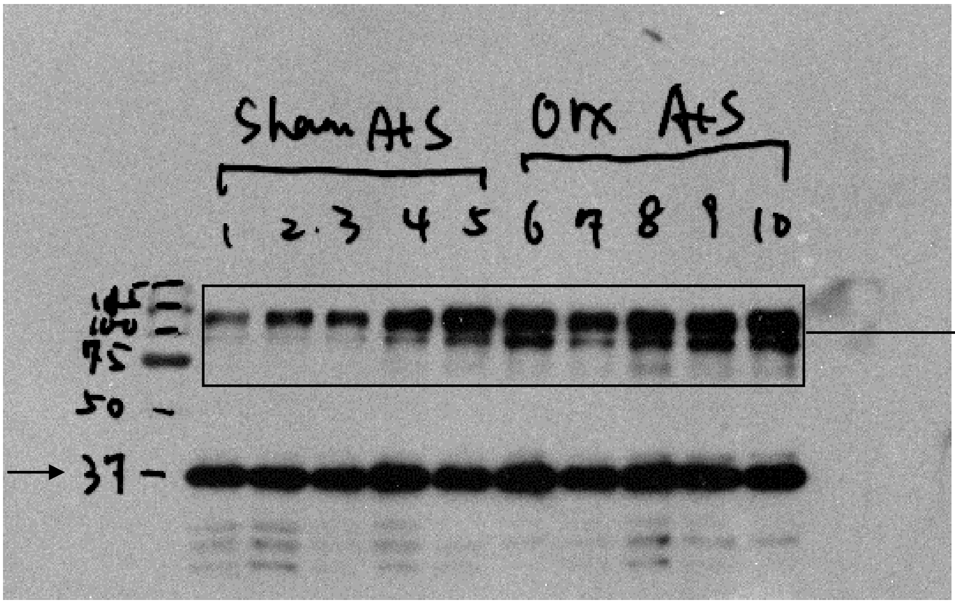

αGAPDH residual after stripping

GAPDH Immunoblot

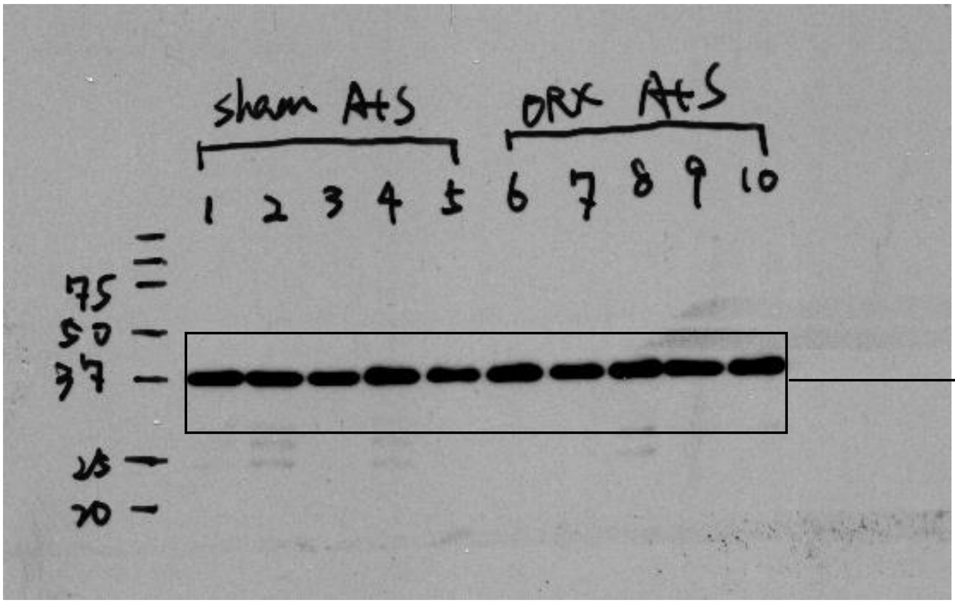

Representative Immunoblot Shown in Figure 9E

Full Unedited Gel 1 for Figure 9K

Representative Gel Shown
in Figure 9K

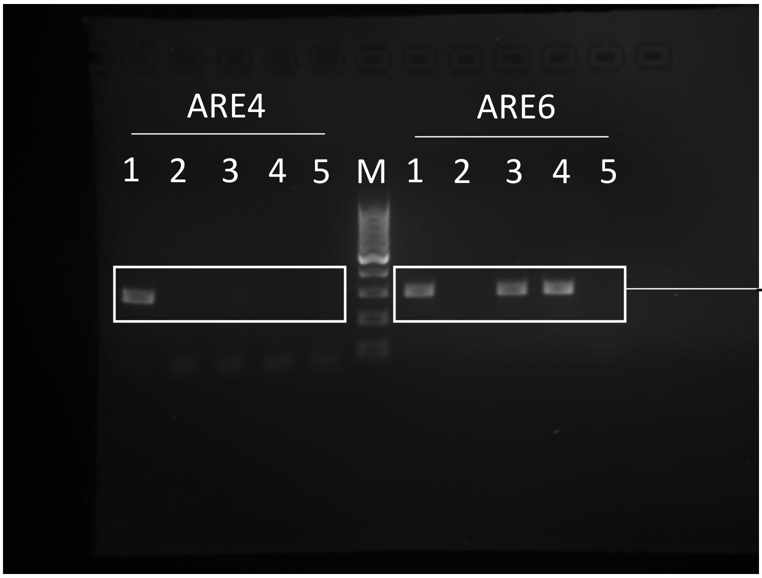

1. Input
2. IP: IgG
3. IP: AR (Santa Cruz)
4. IP: AR (Millipore)
5. NTC (no template control)

Full Unedited Gel 2 for Figure 9K

ARE6

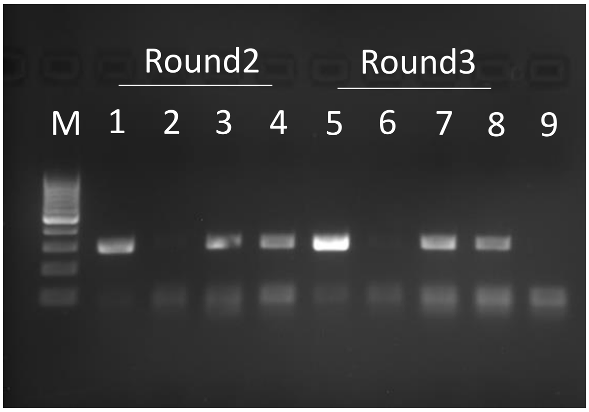

ARE4

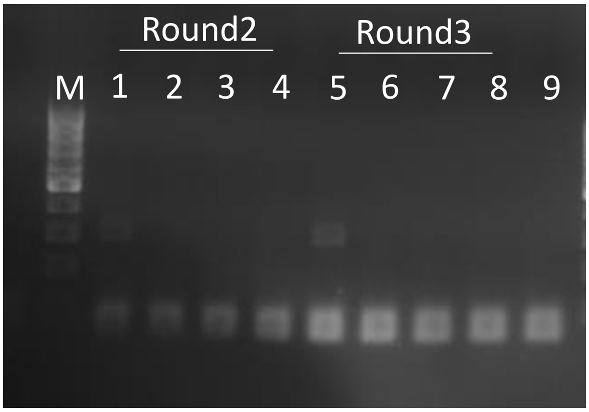

1 and 5. Input
2 and 6. IP: IgG
3 and 7. IP: AR (Santa Cruz)
4 and 8. IP: AR (Millipore)
9. NTC

Full Unedited Immunoblot for Figure 9N

AR Immunoblot

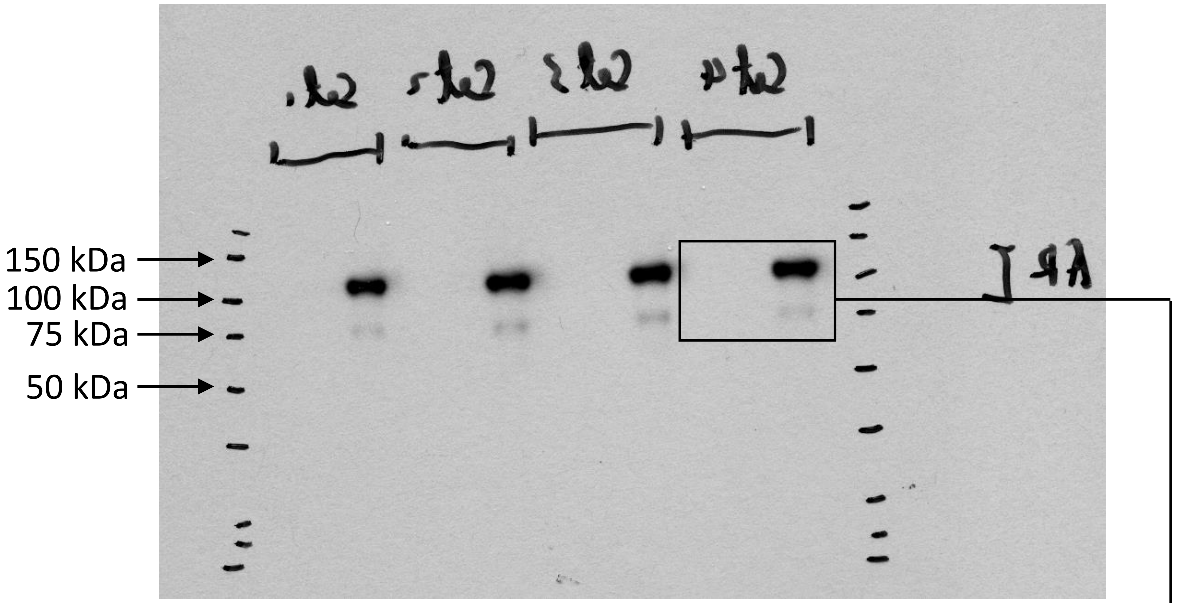

GAPDH Immunoblot

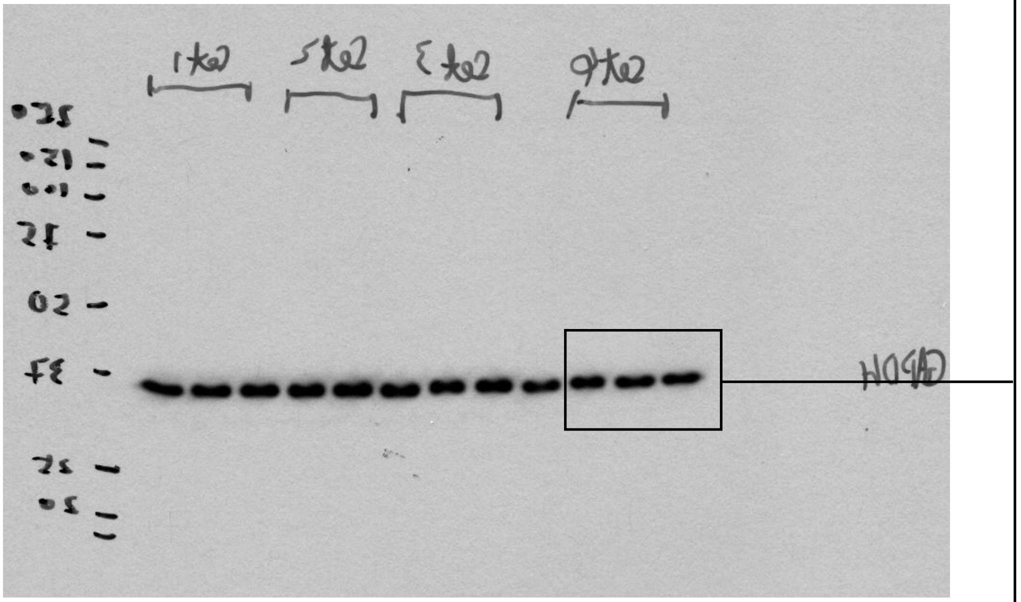

Representative Immunoblot
Shown in Figure 9N

Full Unedited Immunoblot for Supplemental Figure 23G

PD-1 Immunoblot

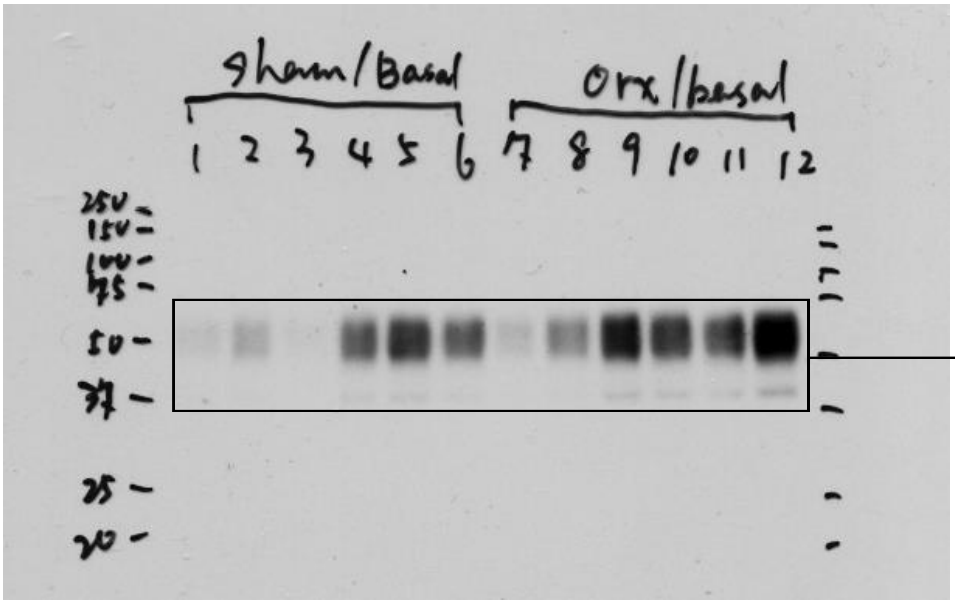

CD3ε Immunoblot

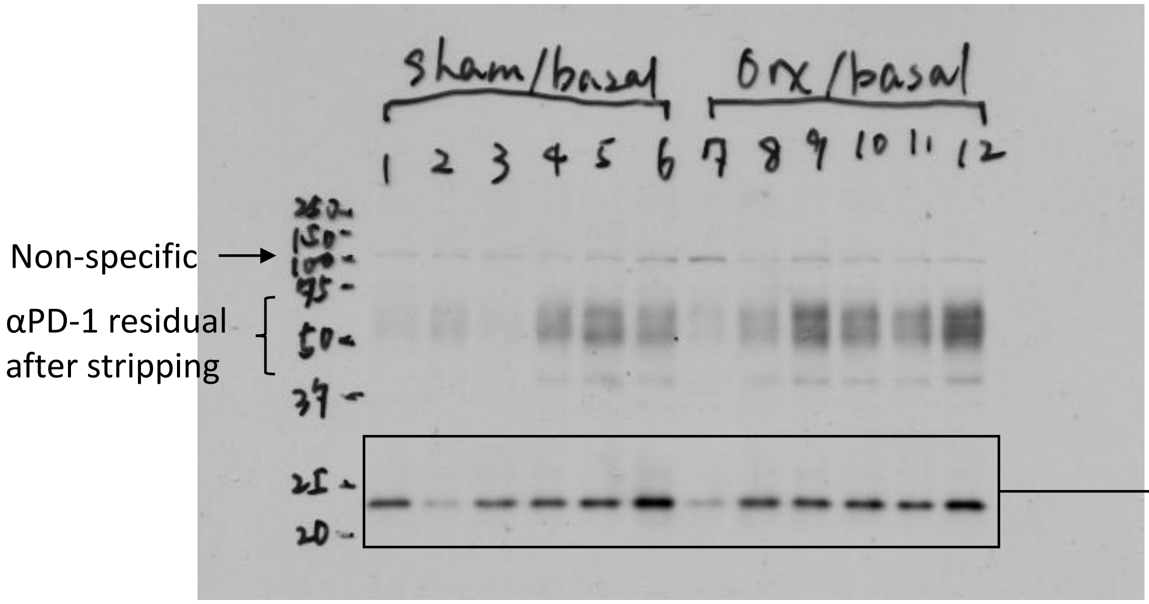

Representative Immunoblot
Shown in Supplemental Figure 23G

Full Unedited Immunoblot for Supplemental Figure 23G

CD19 Immunoblot

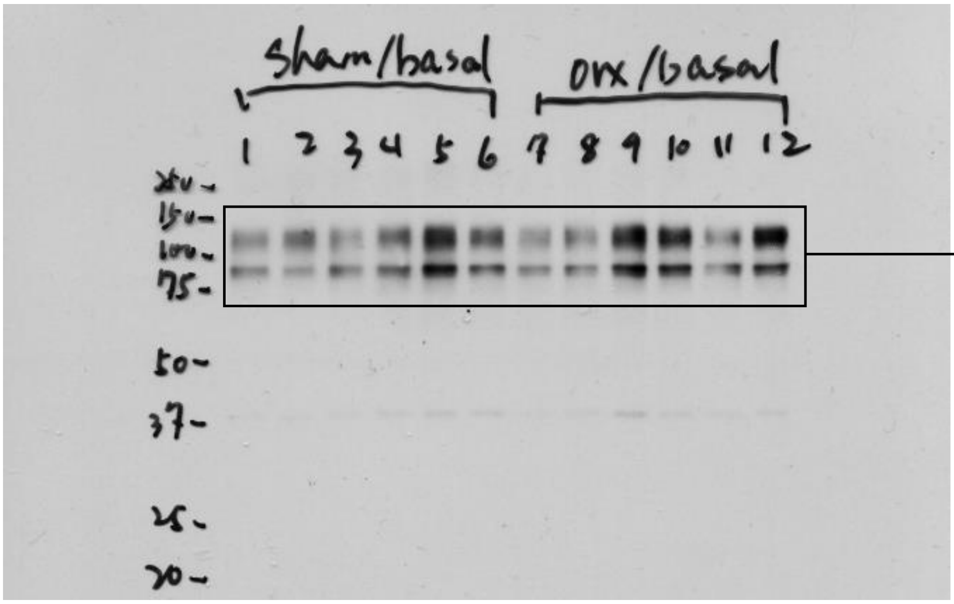

GAPDH Immunoblot

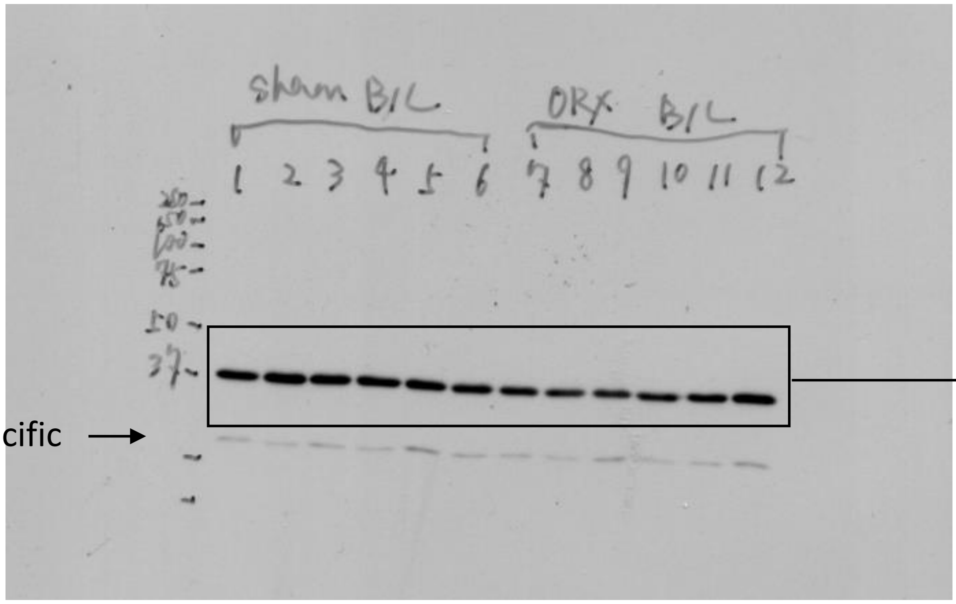

Non-specific →

Representative Immunoblot
Shown in Supplemental Figure 23G

Full Unedited Immunoblot for Supplemental Figure 26C

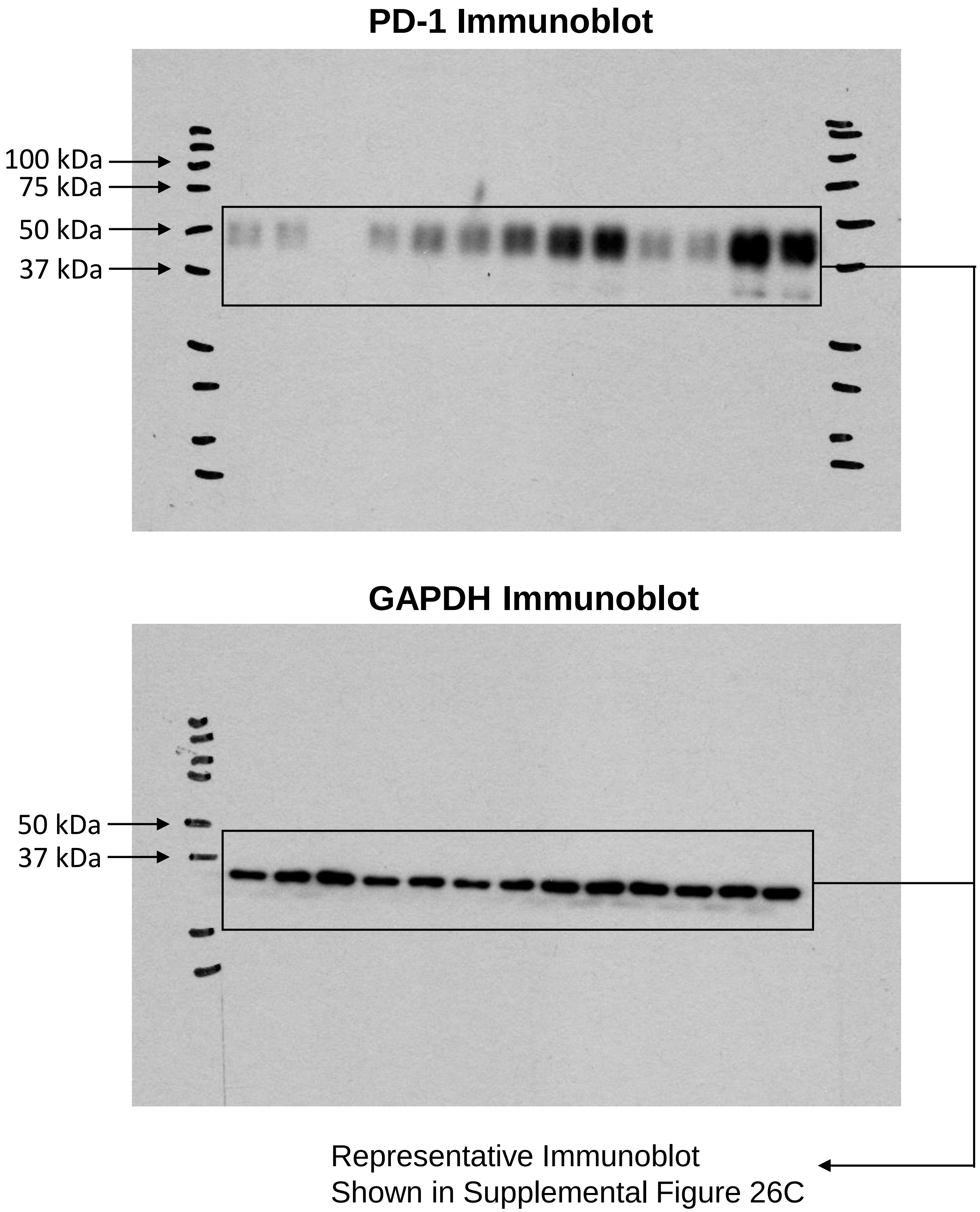

Full Unedited Gel for Supplemental Figure 27A

WT: C57/BL6J
KO: Global PD-1 knockout

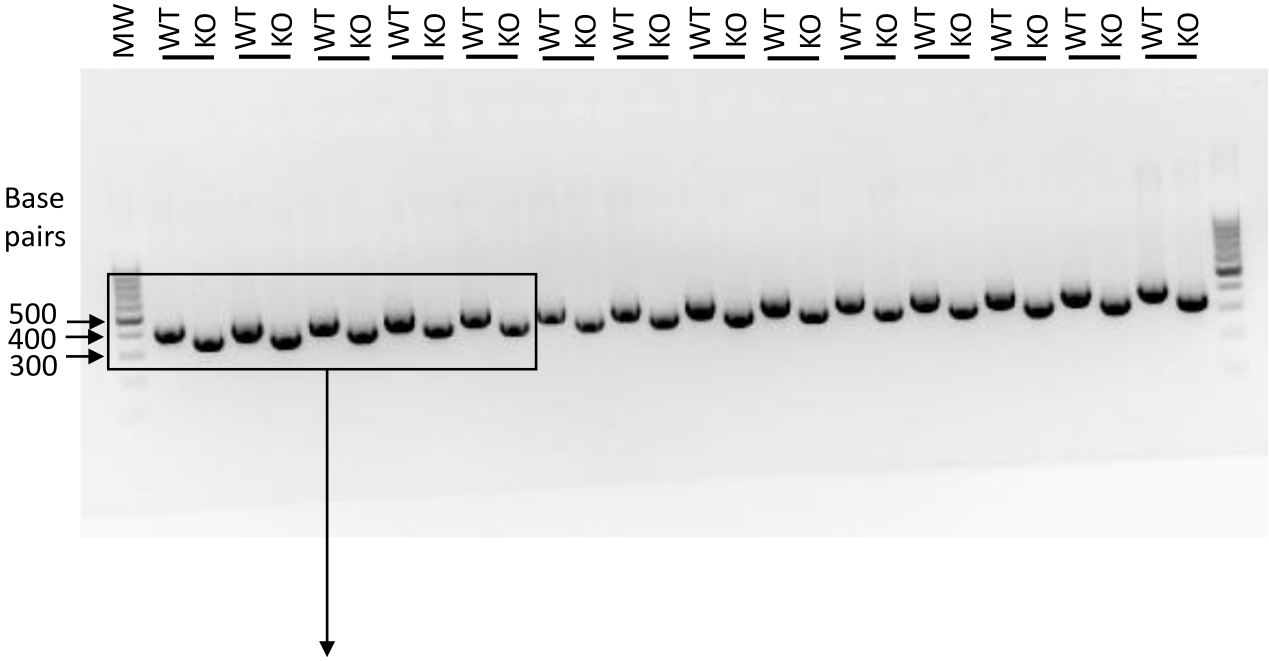

Representative Gel Shown in Supplemental Figure 27A

Full Unedited Immunoblot for Supplemental Figure 27B

PD-1 Immunoblot

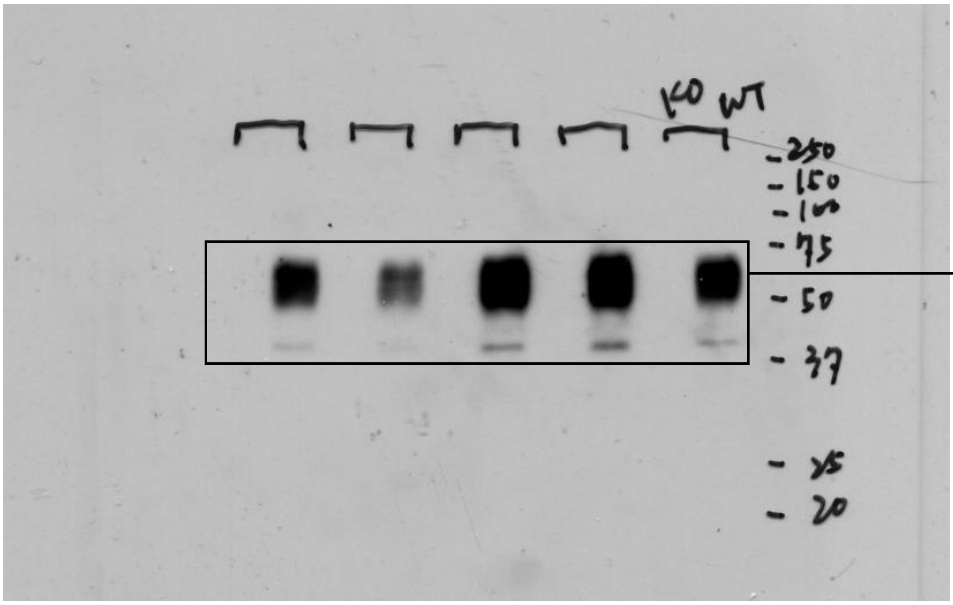

GAPDH Immunoblot

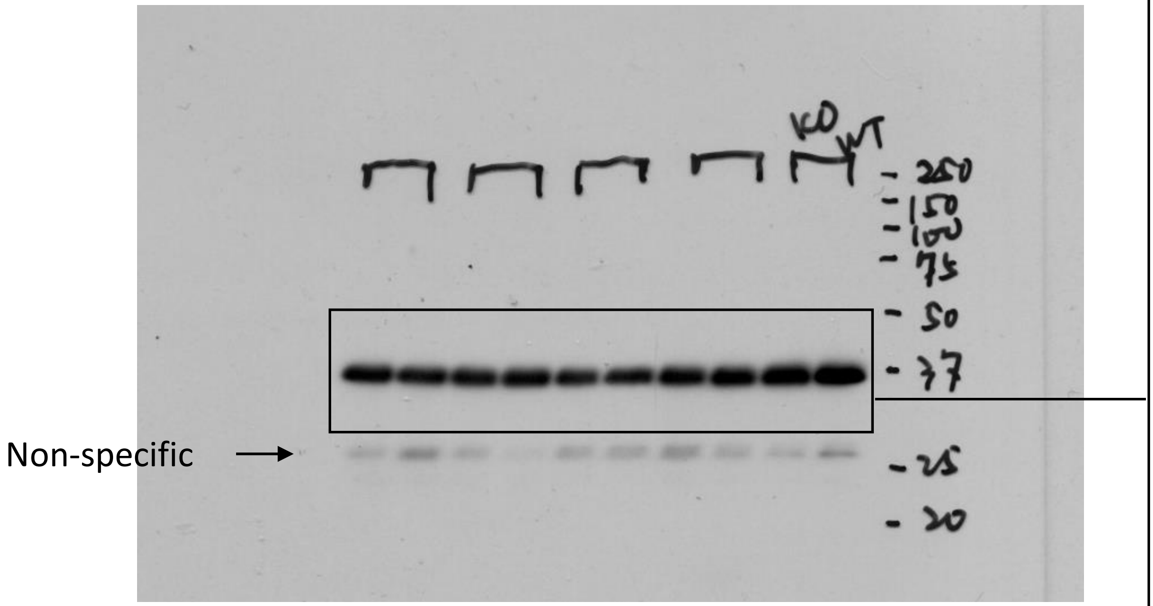

Representative Immunoblot
Shown in Supplemental Figure 27B
